# Type I interferon/IRF7 axis instigates chemotherapy-induced immunological dormancy in breast cancer

**DOI:** 10.1038/s41388-018-0624-2

**Published:** 2018-12-13

**Authors:** Qiang Lan, Sanam Peyvandi, Nathalie Duffey, Yu-Ting Huang, David Barras, Werner Held, François Richard, Mauro Delorenzi, Christos Sotiriou, Christine Desmedt, Girieca Lorusso, Curzio Rüegg

**Affiliations:** 1 Pathology Unit, Department of Oncology, Microbiology and Immunology (OMI), Faculty of Science and Medicine, University of Fribourg, 1700 Fribourg, Switzerland; 2 National Center for Competence in Research (NCCR), Molecular Oncology, Swiss Institute for Experimental Cancer Research (ISREC)-Ecole Polytechnique Fédérale de Lausanne (EPFL), 1015 Lausanne, Switzerland; 3 SIB-Swiss Institute of Bioinformatics, 1015 Lausanne, Switzerland; 4 Ludwig Institute for Cancer Research, 1066 Epalinges, Switzerland; 5 Department of Fundamental Oncology, University of Lausanne, 1011 Lausanne, Switzerland; 6 Breast Cancer Translational Research Laboratory, Institut Jules Bordet, Université Libre de Bruxelles, 1000 Brussels, Belgium; 7 Swiss Integrative Center for Human Health, Fribourg, Switzerland; 8 Present Address: Developmental Biology Program, Institute of Biotechnology, University of Helsinki, Helsinki, Finland

**Keywords:** Immunology, Breast cancer

## Abstract

Neoadjuvant and adjuvant chemotherapies provide survival benefits to breast cancer patients, in particular in estrogen receptor negative (ER^−^) cancers, by reducing rates of recurrences. It is assumed that the benefits of (neo)adjuvant chemotherapy are due to the killing of disseminated, residual cancer cells, however, there is no formal evidence for it. Here, we provide experimental evidence that ER^−^ breast cancer cells that survived high-dose Doxorubicin and Methotrexate based chemotherapies elicit a state of immunological dormancy. Hallmark of this dormant phenotype is the sustained activation of the IRF7/IFN-β/IFNAR axis subsisting beyond chemotherapy treatment. Upregulation of IRF7 in treated cancer cells promoted resistance to chemotherapy, reduced cell growth and induced switching of the response from a myeloid derived suppressor cell-dominated immune response to a CD4^+^/CD8^+^ T cell-dependent anti-tumor response. IRF7 silencing in tumor cells or systemic blocking of IFNAR reversed the state of dormancy, while spontaneous escape from dormancy was associated with loss of IFN-β production. Presence of IFN-β in the circulation of ER^−^ breast cancer patients treated with neoadjuvant Epirubicin chemotherapy correlated with a significantly longer distant metastasis-free survival. These findings establish chemotherapy-induced immunological dormancy in ER^−^ breast cancer as a novel concept for (neo)adjuvant chemotherapy activity, and implicate sustained activation of the IRF7/IFN-β/IFNAR pathway in this effect. Further, IFN-β emerges as a potential predictive biomarker and therapeutic molecule to improve outcome of ER^−^ breast cancer patients treated with (neo)adjuvant chemotherapy.

## Introduction

Chemotherapy is widely used for the treatment of breast cancer. While estrogen receptor negative (ER^−^) or triple-negative breast cancer (TNBC) is generally associated with unfavorable prognosis, neoadjuvant and adjuvant chemotherapies provide significant survival benefits to about one third of these patients [1]. In case of progression, ER^−^ breast cancers relapse with a bimodal distribution, with peaks at 1–2 years and 4–5 years after surgery, followed by a tailed extension up to 10 years [2, 3]. This profile suggests that disseminated tumor cells (DTC) evolve with a discontinuous growth kinetics [3, 4]. Viable, dormant DTC and micro-metastases have been identified in animal models [5, 6] and breast cancer patients [7]. Hence, the concept of tumor dormancy was introduced, whereby surviving cancer cells remain quiescent and clinically silent for prolonged periods of time before resuming growth and causing clinically-manifest relapses. While long term dormancy has been typically associated with ER^+^ breast cancer due to their late relapses, the discontinuous growth kinetics of relapses in ER^−^ cancers suggests that a dormancy also applies to ER^−^ [4, 8, 9]. Three non-mutually exclusive forms of cancer dormancy have been described: cellular dormancy, whereby cancer cells enter a state of cell cycle arrest (i.e., G0-G1) and survive as disseminated single cells or small cell clusters; [9] angiogenic dormancy, a state where cancer cells proliferate but die of starvation due to lack angiogenesis; [10] immunological dormancy, in which disseminated cancer cells are kept under control by the immune system [9] in a process referred to as cancer immune-editing [11]. This consists of three phases: elimination, when cancer cells are recognized and killed by the immune system; equilibrium, when the immune system controls but does not completely eliminate malignant cells, and escape, when residual tumor cells avoid immune control and resume growth [11, 12]. Mechanisms underline immunological dormancy in solid tumors, including breast cancer, are not well characterized.

Recently, chemotherapy-induced anti-tumor immune responses have been reported [13]. Emerging evidence indicates that tumor infiltrating lymphocytes (TILs) actively contribute to the response to chemotherapy and clinical outcome in breast cancer [14, 15]. This is particularly relevant to TNBC as these cancers are infiltrated by TILs, particularly CD8^+^ T-cells [14, 16, 17], In TNBC, elevated TIL levels are associated with an improved pathological complete response following chemotherapy [18], decreased rates of recurrences and improved survival [14, 17, 19]. TILs infiltration in TNBC and HER2^+^ breast cancers is being considered as a potential biomarker with prognostic and predictive values [20].

Type I interferons (IFNs) are potent regulators of the immune response, including in cancer [21, 22]. High level of type I IFN-regulated MxA protein closely relates to TILs infiltration and is an independent prognostic factor for disease-free survival in TNBC [23]. Type I IFN signaling is up-regulated in tumors responding to chemotherapy, persisted in residual tumor cells in patient-derived xenografts (PDX) [24] and is necessary for the efficacy of some chemotherapies [25]. However, the expression of a type I IFN-related DNA-damage resistance signature (IRDS) was reported to correlate with resistance to chemotherapy and radiotherapy in multiple cancer types, including of the breast [26, 27].

Here we show that ER^−^ breast cancer cells surviving chemotherapy induced a strong T cell response mediating immunological tumor dormancy. Activation of the IRF7/IFN-β/IFNAR pathway is critical in this process.

## Results

### In vitro chemotherapy treatment durably alters in vitro and in vivo 4T1 cell growth

**Fig. 1:**
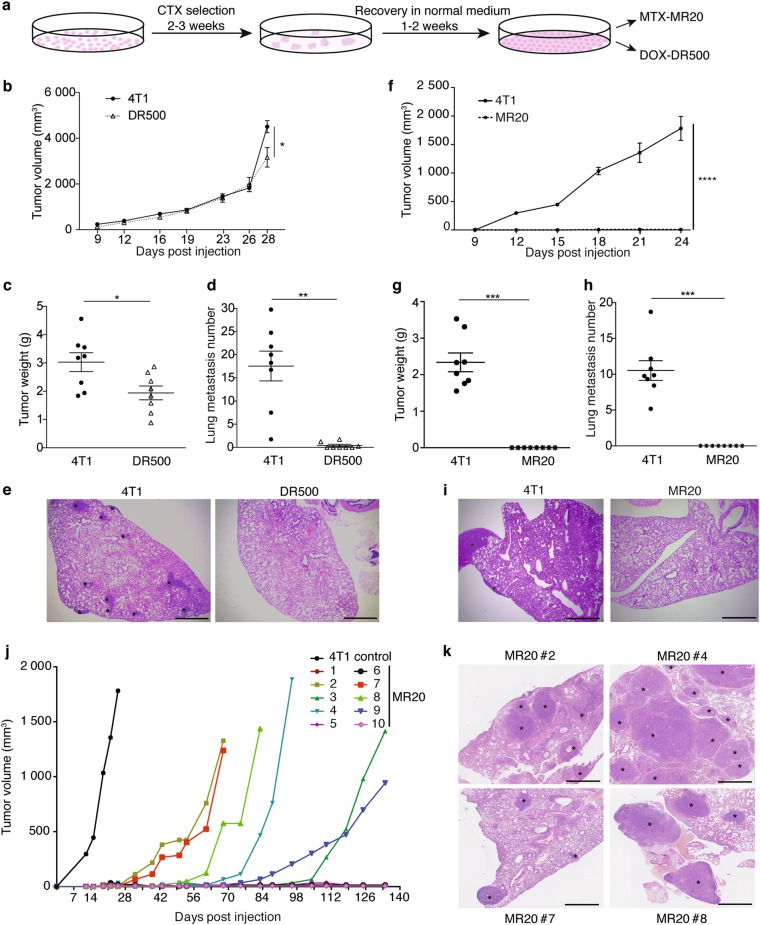
Breast cancer cell treatment with high-dose chemotherapy in vitro induces tumor dormancy in vivo. **a** Experimental design of chemotherapy treatment of tumor cells in vitro. **b** Primary tumor growth in BALB/c mice injected with 4T1 and DR500 tumor cells (*n* = 8/group). **c–d** Quantification of tumor weight **c** (*n* = 8) and lung metastatic nodule numbers **(d)** (*n* = 8) at day 28 post injection in the mice of panel **b. e** Illustrative sections of lungs from mice injected with 4T1 and MR20 tumor cells 25 days post injection (H&E staining). Asterisks denote presence of metastasis (scale bar: 1 mm). **f** Primary tumor growth in BALB/c mice injected with 4T1 and MR20 tumor cells (*n* = 8). **g–h** Quantification of tumor weight **(g)** and lung metastatic nodule numbers **(h)** at day 28 post injection in the mice of panel **(f)** (*n* = 8). **i** Illustrative sections of lungs from mice injected with 4T1 and MR20 tumor cells 28 days post injection (H&E staining). Asterisks denote presence of metastasis (scale bar: 1 mm). **j** Long-term monitoring of tumor development in BALB/c mice injected with MR20 cells. Each tumor growth curves correspond to one individual mouse as indicated (*n* = 10/group). 4T1 tumor growth (black curve) is shown as the average tumor volume of 8 mice. These mice are sacrificed at day 25 due to large tumor volume. **k** Representative sections of lung metastases detected in MR20-injected mice upon progression (H&E staining). Asterisks denote presence of metastasis (scale bar: 1 mm). Data are represented as mean ± SEM. *P* values: * < 0.05; *** < 0.0005, unpaired two-tailed Student’s *t* test

In order to expose tumor cells to chemotherapy under well controlled conditions, we treated the metastatic triple-negative murine mammary adenocarcinoma cell line 4T1 in vitro with two drugs used for ER^−^ breast cancer treatment: the antimetabolite Methotrexate (MTX), and the anthracycline Doxorubicin (DOX) [1, 28, 29]. To simulate maximum tolerated dose (MTD) chemotherapy, 4T1 cells were treated over 2–3 weeks’ time with doses killing over 85% of the cells (IC85) within 48 h (Supplementary Fig. 1a), until surviving colonies formed (Fig. 1a). Two of the obtained lines were named MR20 (MTX resistant at 20 ng/ml) and DR500 (DOX resistant at 500 ng/ml), respectively (Supplementary Fig. 1b). The in vitro growth of these cells was slower compared to parental 4T1 cells (Supplementary Fig. 1c). When implanted orthotopically into the 4^th^ mammary fat pad (MFP) of immune competent syngeneic BALB/c mice, 4T1 cells formed rapidly growing and metastatic tumors, DR500 cells formed growing tumors but with rare lung metastasis (Fig. 1b–e) and MR20 cells did not form any tumors or lung metastasis within the usual time frame (Fig. 1f–i).

### MR20 cell dormancy in immunocompetent mice

In MR20-injected mice, however, some tumors formed starting one month after implantation and at 3.5 months, 6 out of 10 mice (60%) had primary tumors (Fig. 1j) and lung metastases (Fig. 1k). The remaining mice (40%) remained tumor-free for over one-year without evidence of tumor cells in the MFP at sacrifice. These results are reminiscent of the discontinuous kinetics seen in patients after initial therapy and consistent with a state of dormancy [3, 4, 30].

Taken together, these results demonstrate that 4T1 cells that survived high-dose DOX or MTX chemotherapy in vitro formed latent, dormant tumors in vivo. While in MR20 cells dormancy occurred in primary tumor and metastasis, in DR500 cells dormancy was evident in metastasis only. To investigate the mechanism of dormancy, we focused mainly on MR20 cells since dormancy was already evident at the primary site.

### MR20 cells are cell cycle proficient but show increased apoptosis in vitro

To characterize the reduced MR20 cell growth in vitro we first analyzed expression of the proliferation marker Ki67. This was expressed in over 95% of MR20 and 4T1 cells (Supplementary Fig. 2a, b). Cell cycle analysis indicated no difference in the distribution of the cycle phases, including no increase in the G0/G1 fraction typical of cellular dormancy (Supplementary Fig. 2c, d). However, we observed a higher proportion of MR20 apoptotic cells by Annexin V and active Caspase 3 staining compared to 4T1 cells (9.95% vs. 4.8%) (Supplementary Fig. 2e, f). In addition 4T1 cells lose the CMFDA membrane labeling faster than MR20 cells (110 vs. 44 times diluted, respectively) (Supplementary Fig. 2g).

These results indicate that chemotherapy-resistant MR20 cells have no slower cell cycle progression but increased rate of apoptosis compared to 4T1 cells. While these alterations exclude cellular dormancy, they do not explain their latency and delayed growth in vivo.

### MR20 cells induce a T and B cell-prevalent immune response while 4T1 cells promote expansion of MDSCs

**Fig. 2:**
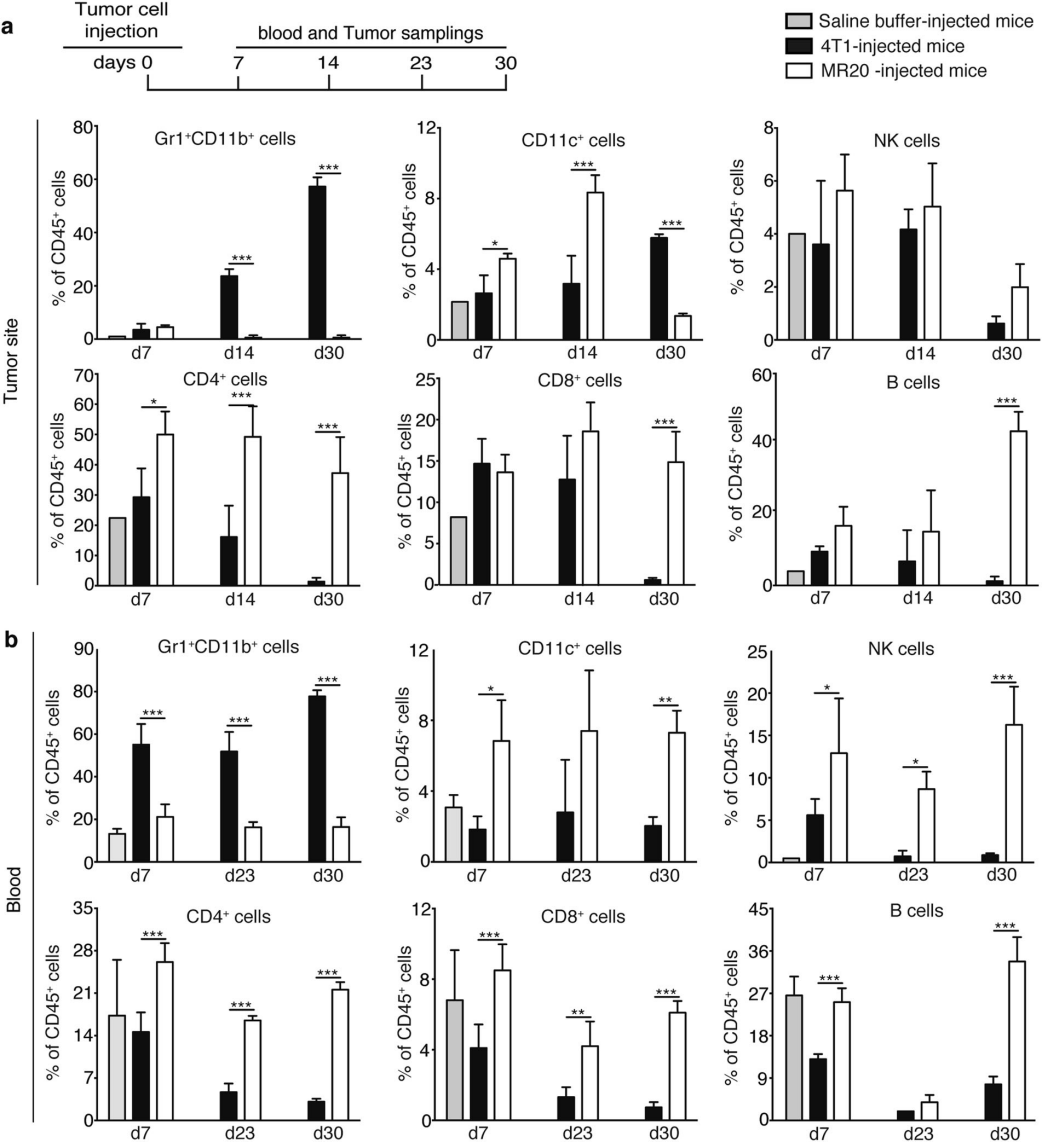
MR20 tumor cells in vivo suppress MDSC mobilization and promote T and B lymphocyte accumulation. **a** Top: Scheme of the protocol used for the analysis of the immune response upon orthotopic MR20 injection into BALB/c mice indicating days of blood collection and tumor removal for flow cytometry analysis. Graphs**:** Percentages of immune cells in the primary tumor site from saline buffer, 4T1-injected or MR20-injected BALB/c mice detected by flow cytometry analysis. **b** Percentages of immune cells in the peripheral blood from saline buffer, 4T1-injected or MR20-injected BALB/c mice detected by flow cytometry analysis. Time points of analysis (days after tumor cells’ injection) are indicated. Data are from one representative experiment. *n* = 5/group. Results are expressed as % of CD45 + cells. Data are represented as mean ± SEM. *P* values: * < 0.05; ** < 0.005; *** < 0.0005 by unpaired two-tailed Student’s *t* test

To characterize the in vivo tumor dormancy, we first considered the angiogenic potential of MR20 cells. However, as there was no detectable tumor mass in MR20-injected MFP within the first 25 days (Fig. 1f), we could not evaluate tumor angiogenesis [10]. Instead, we noticed a remarkable enlargement of the MFP-draining lymph node (LN) in MR20 cell-injected mice (Supplementary Fig. 3a). Histological analysis excluded LN metastatic colonization (Supplementary Fig. 3b). The total cell number in the MFP-draining LN increased from 1.1 × 10^7^ cells, in saline-injected mice, to 2.5 × 10^7^ cells in MR20-injected mice (Supplementary Fig. 3c). Next, we characterized the immune cells in the MFP and in the circulation of BALB/c mice injected with MR20 and 4T1 tumor cells. MR20-injected MFPs had very few MDSCs (Gr1^+^CD11b^+^ cells), similar to naive mice even after 30 days post injection, while 4T1-injected MFPs showed high MDSCs levels, increasing over time (Fig. 2a). Conversely, MR20-injected mice harbored significantly more dendritic cells (CD11b^+^CD11c^+^), CD4^+^, CD8^+^ T and B lymphocytes, particularly at later time points (Fig. 2a). A similar increase of these cells was detected in the blood (Fig. 2b). These results suggest that MR20 cells induce a profound alteration of the local and systemic immune response: from a MDSC-dominated response in 4T1-injected mice to a DC, T and B cell-prevalent response in MR20-injected mice.

### DR500 cells induce a weaker immune response but higher frequency of cytotoxic CD8^+^ T lymphocytes at the dormant metastatic site compared to MR20 cells

DR500 cells formed primary tumors but did not form lung metastases (Fig. 1b–h). To unravel possible differences between DR500 and MR20 tumor cells in their capacity to elicit an immune response in vivo, we characterized the immune cells in the primary tumor, in the circulation and in the lungs of BALB/c mice injected with DR500 vs. MR20 and 4T1 injected mice. At day 15, DR500 tumor cells induced changes similar to those induced by MR20 cells (decrease in Gr1^+^D11b^+^ cells, increased in CD4^+^, CD8^+^ T and B lymphocytes) at the three sites, but to a lesser extent. At day 24 the DR500 suppressive effect on Gr1^+^CD11b^+^ cells disappeared and the inductive effect on CD4^+^, CD8^+^ T and B lymphocytes was attenuated at all sites, particularly in the primary tumor, compared to MR20-injected mice (Supplementary Fig. 4a, b, c). As DR500 injected mice were nevertheless free of lung metastases, we monitored expression of the cytotoxic molecules FasL, GranzymeB and IFNγ in CD8^+^T cells in the lungs of these mice. We observed a higher frequency of cytotoxic molecules in both DR500 and MR20 models compared to 4T1-injected mice but with some differences. The lungs of DR500-injected mice had more FasL, GranzymeB and IFNγ - positive CD8^+^ T cells, particularly at day 24, while in MR20-injected mice the cytotoxic response was observed at earlier time point (Supplementary Fig. 5).

Altogether these results suggest that DR500 cells elicit similar immune response at MR20 cells but of lesser magnitude. At the metastatic site both models show similar cytotoxic differentiation of CD8^+^ T cells.

### CD4^+^ and CD8^+^ lymphocytes are required for MR20 dormancy

**Fig. 3:**
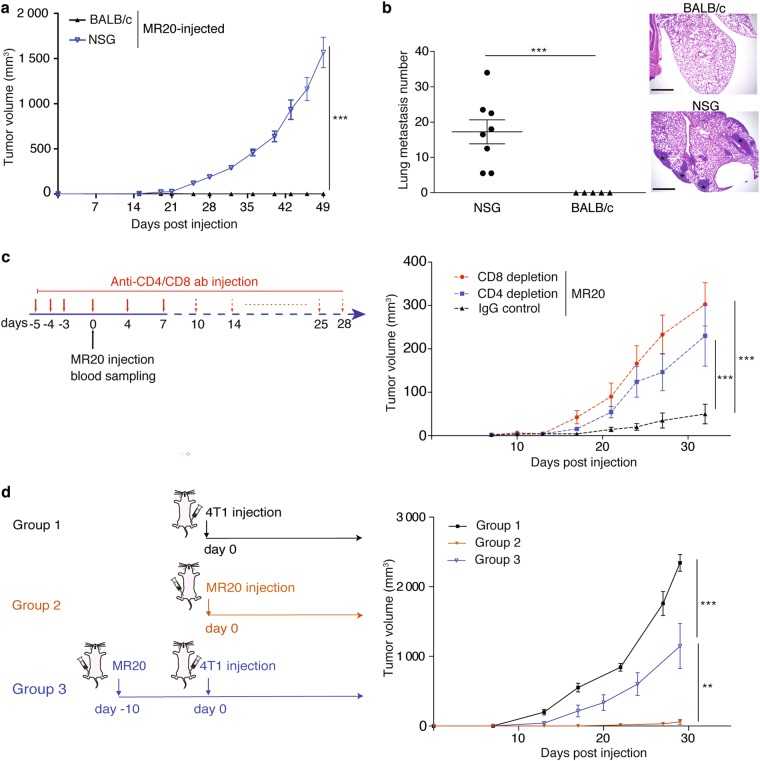
MR20 tumor cell dormancy in vivo requires CD4^+^ or CD8^+^ lymphocytes. **a** Growth curve of MR20-derived tumors injected in NSG and BALB/c mice. *n* = 5–8/group. **b** Quantification of lung metastasis nodules in the mice of experiment in panel a. Micrographs show representative histological sections of mice lungs from the three groups in panel a (H&E staining). Scale bar: 1 mm. **c** Antibody treatment protocol for CD4^+^, CD8^+^ T cell-depletion (left) and MR20 tumor growth curve in IgG isotype control BALB/c injected mice or CD4^+^-T Lymphocyte, CD8^+^-T Lymphocyte-depleted BALB/c mice (right). *n* = 6/group. **d** Graphical scheme of 4T1 and MR20 tumor cell injection in the three groups of BALB/c mice (left): Group 1, 4T1-injected mice; Group 2, MR20-injected mice; Group 3, MR20-injected mice 10 days before day 0 of 4T1 injection into contralateral 4^th^ MFP. *n* = 8/group. Data are represented as mean ± SEM. *P* values: ** < 0.005; *** < 0.0005 by unpaired two-tailed Student’s *t* test

We next asked the question whether the adaptive immune response and in particular T cells were indeed functionally involved in promoting MR20 dormancy. To this end, we injected MR20 cells into immune-compromised NOD-SCID common gamma 2 chain-deficient (NSG) mice that lack mature T, B and NK cells and display impaired DC and macrophage functions [31]. MR20 cells efficiently formed primary tumors and lung metastases in NSG mice (Fig. 3a, b). To demonstrate the involvement of CD4^+^ or CD8^+^ T lymphocytes in controlling MR20 dormancy, we injected MR20 cells into BALB/c mice depleted of CD4^+^ or CD8^+^ T lymphocytes by antibody treatment (Fig. 3c, left panel). Depletion of either CD4^+^ or CD8^+^ T lymphocytes (confirmed by flow cytometry, data not shown), resulted in effective tumor growth (Fig. 3c, right panel). Further we tested whether MR20 cells elicit a protective immune response by injecting MR20 cells in the 4^th^ MFP 10 days before injecting 4T1 parental cells into the contralateral MFP. Preconditioning of mice with MR20 cells reduced 4T1 tumor growth by approximately 50% (Fig. 3d).

These results indicate that CD4^+^ and CD8^+^ T lymphocytes contribute to enforce chemotherapy-induced MR20 tumor dormancy in vivo. The “vaccination effect” of immune preconditioning with MR20 cells suggests that dormancy is not due to the emergence of chemotherapy-induced neo-antigens.

### The transcriptomes of chemotherapy treated, dormant tumor cells are enriched for type I IFN response genes

**Fig. 4:**
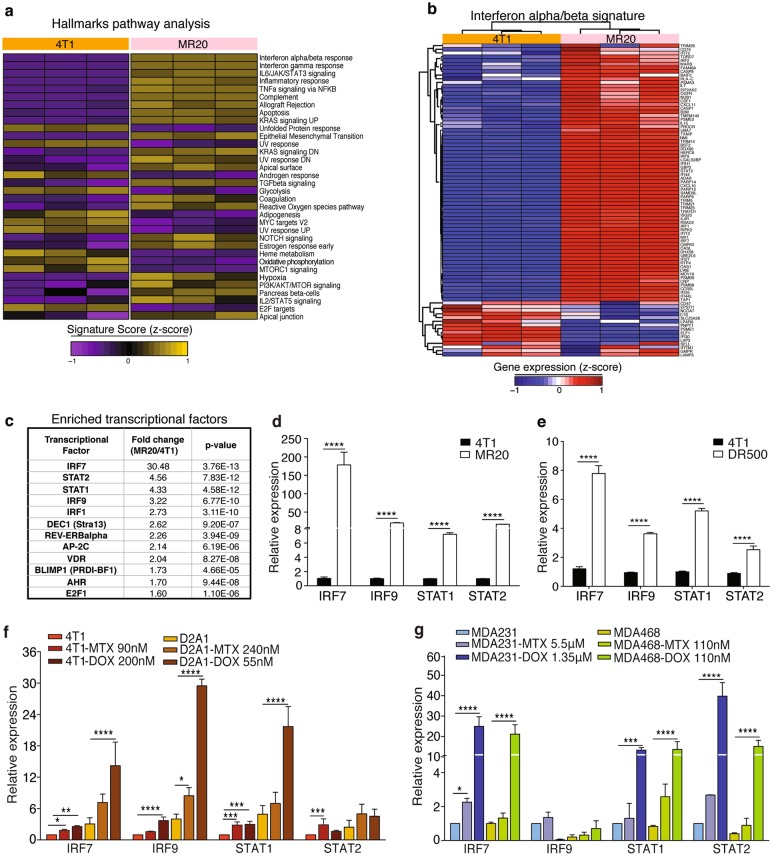
MR20 cells are enriched for type I IFN genes. **a** Heat map of the signature score of the hallmarks pathway analysis in 4T1 and MR20 cells color-coded based on expression levels relative to average (Violet, downregulated; yellow, upregulated). Results from biological triplicate are shown. **b** Heat-map of type I IFN signature-related gene expression in 4T1 cells and MR20 cells, color-coded based on expression levels relative to average (blue, down-regulated; red, up-regulated). The gene list was taken from MSigDB. **c** Top list of enriched transcription factors in MR20 vs. 4T1 cells. **d–e** Relative expression of IRF7, IRF9, STAT1, STAT2 transcripts in **d** 4T1 and MR20 cells, (**e**) 4T1 and DR500 cells. **f**–**g** Relative expression analysis by RT-qPCR of IRF7, IRF9, STAT1, STAT2 mRNAs in **f** 4T1 and D2A1 murine tumor cells or **g** MDA-MB-231(MDA-231) and MDA-MB-468(MDA-468) human tumor cells treated with MTX or DOX for 24 h at the indicated IC85 doses. Data are represented as mean ± SEM. *P* values: * < 0.05; ** < 0.005; *** < 0.0005; **** < 0.0001 by unpaired two-tailed Student’s *t* test

To unravel the molecular basis for the distinct behavior of chemo-treated cells, we performed transcriptome analyses of 4T1, MR20 and DR500 cells. Unsupervised clustering analysis revealed significant differences in the gene expression. Statistical analysis identified 324 and 535 (>2-fold) significantly up-regulated and 234 and 680 (<−2-fold) down-regulated genes in MR20 and DR500 cells compared to 4T1 cells, respectively (Supplementary Table). Both MR20 and DR500 cells were enriched for transcripts of innate and adaptive immune responses, specifically the type I IFN-related signature (Fig. 4a, b and Supplementary Fig. 6a, b). No changes in the expression of transcripts of the main angiogenic pathways were observed, further suggesting that MR20 tumor dormancy was unlikely due to deficient angiogenesis. Transcription factors analysis identified the interferon regulatory factor 7 (IRF7) as most significantly up-regulated in MR20 (Fig. 4c) and DR500 cells (Supplementary Fig. 6c). The upregulation of expression of *IRF7* itself, and of *IRF9*, the signal transducer and activator of transcription (*STAT*) 1 and 2 were confirmed by RT-qPCR in both MR20 and DR500 cells (Fig. 4d, e). To test whether chemotherapy treatment was able to directly induce IRF7 expression in different cell lines, we treated murine (4T1 and D2A1) and human (MDA-MB-231 and MDA-MB-468) breast cancer cell lines for 24 h with high doses (IC85) of MTX and DOX. Indeed, we observed up-regulation *IRF7, IRF9, STAT1 and STAT2* expressions (Fig. 4f, g).

We conclude that MTX and DOX chemotherapies activate type I IFN signaling, which, in surviving cells (MR20, DR500) persists beyond treatment. The expression of *IRF7, IRF9, STAT1, and STAT2* in DR500 cells is weaker compared to MR20 cells.

### Constitutive type I IFN pathway activation in spontaneously dormant tumor cells

The sustained upregulation of type I IFN signaling in dormant tumor cells raised the question of whether the same mechanism is associated with other models of dormancy. To address this question, we used D2A1 and D2.0R cells that were originally derived from spontaneous mammary tumors from a hyperplastic alveolar nodule line. They represent different stages of tumor progression: D2A1 cells are tumorigenic and metastatic while D2.0R are less tumorigenic and form metastases only after long latency times and are considered a model for breast cancer dormancy (Supplementary Fig. 7a). Despite their divergent behavior in vivo, these cells lines readily proliferate in vitro [5, 32, 33].

*IRF7, IRF9, STAT1*, and *STAT2* transcripts were up-regulated in dormant D2.0R cells relative to D2A1 cells (Supplementary Fig. 7b). In D2.0R-injected mice we observed a decrease in Gr1^+^CD11b^+^ cells and an accumulation of CD4^+^ and B lymphocytes or NK cells compared to D2A1 (Supplementary Fig. 6c).

From these data, we conclude that activation of type I IFN signaling pathway also occurs in the D2.0R model of spontaneous tumor dormancy.

### IRF7 silencing sensitizes tumor cells to chemotherapy in vitro and breaks dormancy in vivo

**Fig. 5:**
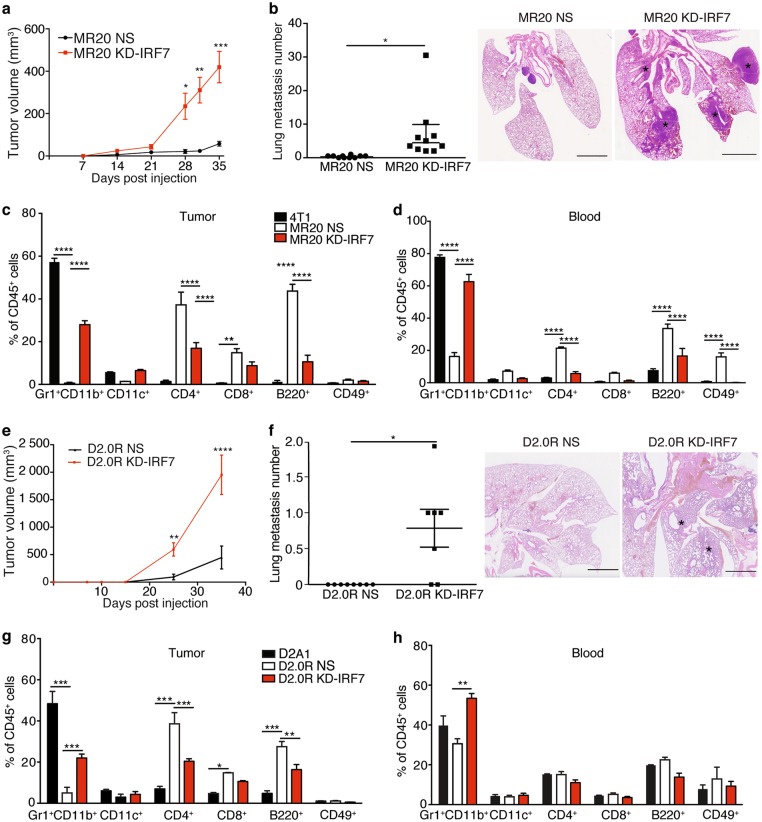
Silencing of IRF7 expression breaks tumor dormancy. **a** and **e** Growth curve of tumors in BALB/c mice injected with **a** MR20 cells silenced for IRF7 (KD-IRF7) or non-silenced cells (NS). *n* = 10/group. or **e** D2.0R cells silenced for IRF7 (KD-IRF7) or non-silenced cells (NS). *n* = 8/group. **b** and **f** Quantification of lung metastases in mice of experiment in panel **a** or **e** and representative images of histological lung sections (H&E staining). Asterisks denote presence of metastases. *n* = 10/group. Scale bar: 1 mm. **c** and **d** Frequency of immune cells in the primary tumor site (**c**) or peripheral blood (**d**) determined by flow cytometry analysis from BALB/c mice injected with 4T1, MR20 NS and IRF7-silenced MR20 (MR20 KD-IRF7) cells. *n* = 5/group. **g** and **h** Frequency of immune cells in the primary tumor site (**g**) or peripheral blood (**h**) determined by flow cytometry analysis from BALB/c mice injected with D2A1, D2.0R NS and IRF7-silenced D2.0R (D2.0R KD-IRF7) cells. *n* = 5/group. Results are expressed as % of CD45^+^ cells and represent mean values. Data are represented as mean ± SEM. *P* values: * < 0.05; ** < 0.005; *** < 0.0005; **** < 0.0001 by unpaired two-tailed Student’s *t* test and two-way ANOVA

To functionally validate the role of IRF7 in tumor dormancy, we stably silenced IRF7 expression in MR20 and D2.0 R tumor cells by lentiviral-mediated shRNA transduction (Supplementary Fig. 8a, b). IRF7 silencing also decreased the expression of *IRF9*, *STAT1* and *STAT2* transcripts (Supplementary Fig. 8a, b), consistent with published observations [34]. IRF7-silenced cells were more sensitive to MTX and proliferate faster compared to non-silenced (NS) MR20 and D2.0R controls (Supplementary Fig. 8c, d, e, f). Importantly, when injected into immune competent BALB/c mice, IRF7-silenced MR20 and D2.0R cells formed growing (Fig. 5a–e) and metastatic (Fig. 5b–f) tumors. Analysis of the immune cells at the tumor site and in peripheral blood 30 days post injection, revealed that IRF7 silencing caused an increase in Gr1^+^CD11b^+^ cells and a decrease in CD4^+^, CD8^+^ T and B lymphocytes in both models (Fig. 5c–h).

Taken together, these results demonstrate that elevated expression of IRF7 promotes tumor resistance against chemotherapy and switches the MDSCs-dominated response into a T and B cell-prevalent immune response maintaining dormancy.

### IFN-β mediates IRF7-dependent tumor dormancy

**Fig. 6:**
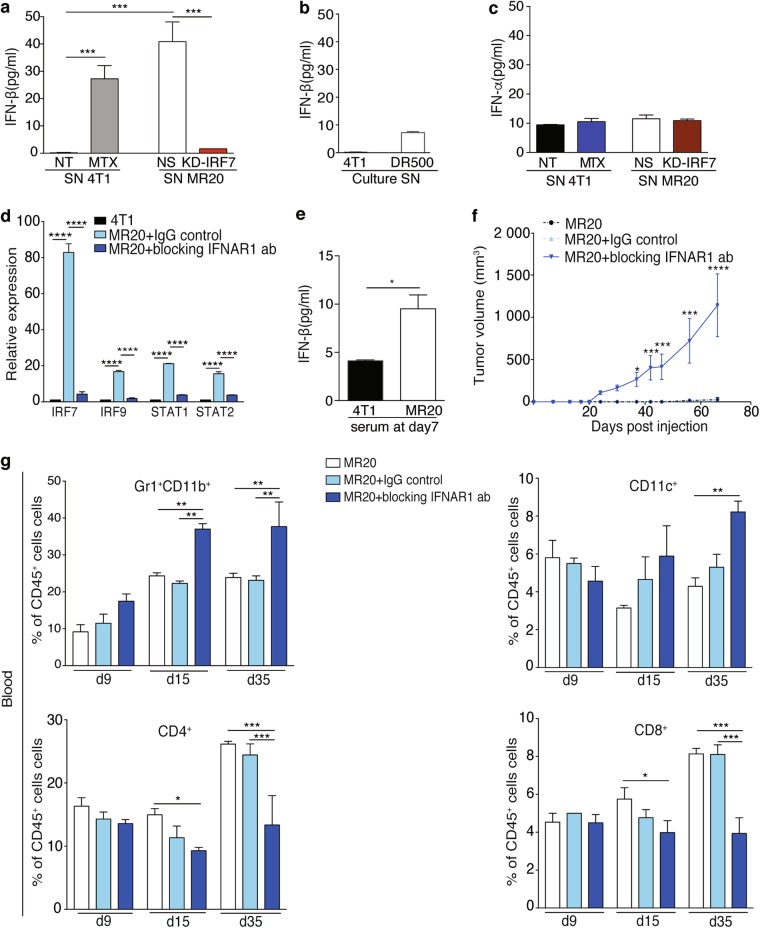
Interferon β/IFNAR axis mediates IRF7-dependent MR20 tumor dormancy. **a**–**b, a** IFN-β levels in the conditioned culture supernatant of 4T1 untreated (NT) and 4T1 MTX-treated tumor cells (90 nM for 24 h), MR20 NS non-silenced, IRF7-silenced MR20 (MR20 KD-IRF7) tumor cells and DR500 **(b)** tumor cells determined by ELISA. **c** IFN-α levels in the conditioned culture supernatant of 4T1 untreated (NT) and 4T1 MTX-treated tumor cells (90 nM for 24 h) or MR20 NS non-silenced and IRF7-silenced MR20 (MR20 KD-IRF7) tumor cells determined by ELISA. **d** Relative expression of IRF7, IRF9, STAT1 and STAT2 transcripts in 4T1 and MR20 tumor cells cultured for 24 h in the presence of a blocking-antibody against IFNAR1 subunit (20 ng/ml I.P.) or its IgG isotype control. **e** Levels of IFN-β in the serum of BALB/c mice 7 days after injection of 4T1 and MR20 tumor cells (into the MFP) determined by ELISA. **f** Growth curve of MR20-injected BALB/c mice systemically treated with anti-IFNAR1 blocking-antibody, its control IgG or no antibody, as indicated. *n* = 8/group. **g** Analysis of immune cells in the peripheral blood of MR20-injected BALB/c mice treated with anti-IFNAR1 blocking-antibody, its control IgG or no antibody, as indicated. Blood analysis performed by flow cytometry at days 9, 15 and 35 post tumor injection. Data are from one representative experiment. Results are expressed as % of CD45^+^ cells and represent mean values. *n* = 5/group. Data are represented as mean ± SEM. *P* values: * < 0.05; ** < 0.005; *** < 0.0005; **** < 0.0001 by unpaired two-tailed Student’s *t* test and one-way ANOVA

As IRF7 is a key transcriptional regulator of type I IFN expression [35], we considered type I IFNs as potential effectors of dormancy. To test this hypothesis, we first measured IFN-α and-β levels in culture supernatants of 4T1, DR500, MR20, MR20 KD-IRF7 and 4T1 cells treated for 24 h with MTX. IFN-β level was very low (<1 pg/ml) in the supernatant of 4T1 cells but high (over 40-fold increase) in the supernatants of MR20 cells and MTX-treated 4T1 cells, and IRF7 silencing in MR20 cells decreased IFN-β levels (Fig. 6a). DR500 cells secreted IFN-β at much lower levels compared to MR20 cells (7 vs. 40 pg/ml) (Fig. 6b). IFN-α levels were uniformly low (<10 pg/ml) in all tested conditions (Fig. 6c). Significantly higher levels of IFN-α and-β were detected in the supernatants of D2.0R cells compared to D2A1 cells (Supplementary Fig. 9a, b), and IRF7 silencing in D2.0R cells suppress IFN-β production (Supplementary Fig. 9a). These results suggest a potential correlation between type I IFN production and dormancy. Further, we monitored type I IFN receptor (IFNAR) expression and function. Flow cytometry analysis revealed that 4T1, D2A1, MR20, and D2.0R cells express IFNAR with a trend toward higher expression in dormant cells (Supplementary Fig. 9c). We then exposed 4T1 or D2A1 cells to exogenous IFN-β or IFN-α (50 ng/ml for 24 h) and observed increased expression of *IRF7, IRF9, STAT1*, and *STAT2* transcripts (Supplementary Fig. 9d, e). Conversely, culture of MR20 cells in the presence of a blocking antibody against the α subunit of IFNAR (i.e., IFNAR1) reduced *IRF7, IRF9, STAT1* and *STAT2* expression (Fig. 6d). These results demonstrate functional and autocrine activation of the IFN-β/IFNAR/IRF7 pathway in 4T1 and MR20 cells. Consistent with these results, the IFN-β levels in the serum of MR20 cell-injected mice, 7 days post-injection, was higher compared to levels in 4T1 cell-injected mice (Fig. 6e). To investigate the direct effect of IFN-β on dormancy in vivo, we treated MR20-bearing mice with the anti-IFNAR1 blocking antibody. This resulted in the formation of MR20-derived tumors (Fig. 6f) associated with increased MDSCs and reduced CD4^+^ and CD8^+^ T lymphocyte infiltration (Fig. 6g). Interestingly, MR20 cells (A1, A3 and B2) that escaped dormancy in vivo (See Fig. 1j) no longer secrete IFN-β, further supporting a direct role for IFN-β expression in maintaining dormancy (Supplementary Fig. 8f).

Altogether, these results indicate that an active IFN-β/IFNAR/IRF7 pathway is critical to maintain dormancy in vivo.

### Presence of IFN-β in the serum of ER^−^ breast cancer patients during Epirubicin neoadjuvant chemotherapy is associated with longer distant metastasis-free survival

**Fig. 7:**
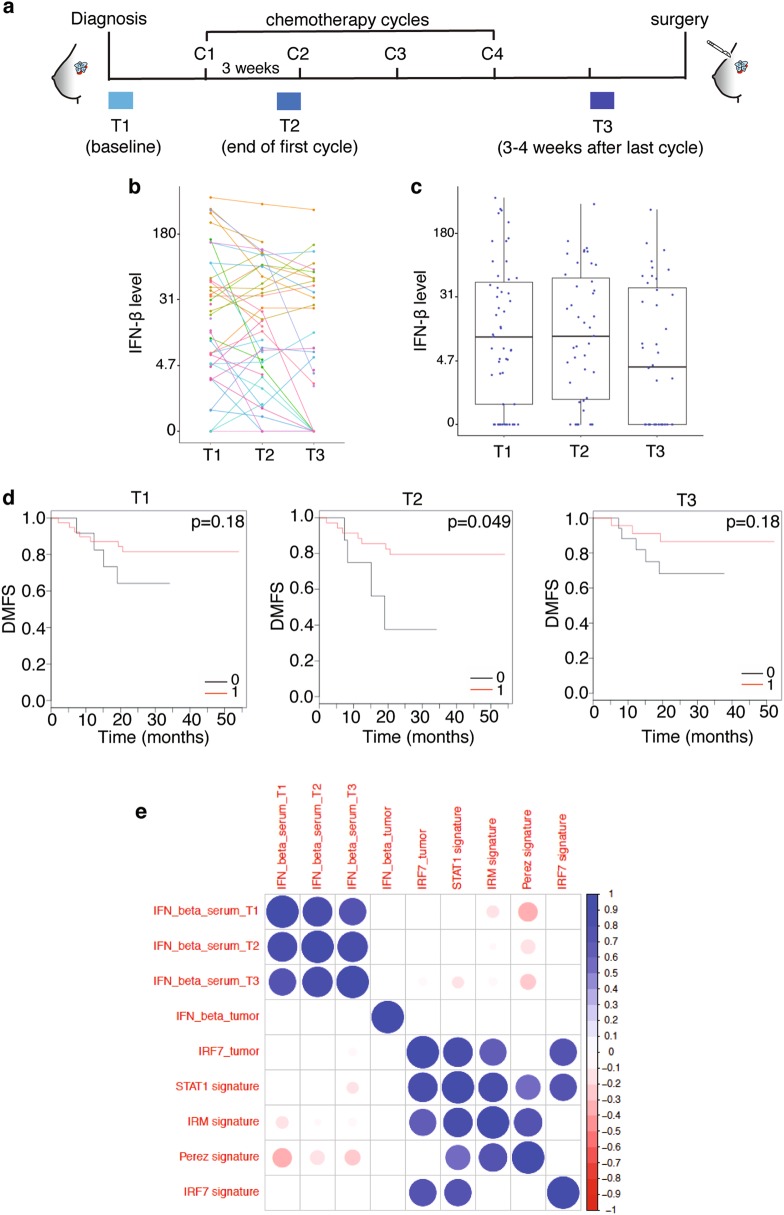
Absence of circulating IFN-β in breast cancer patients at time of neoadjuvant chemotherapy correlates with a shorter DMFS. **a** Scheme of the experimental design for the analysis of IFN-β in breast cancer patients’ sera. Time points are: T1, baseline before chemotherapy at time of tumor diagnosis; T2, after the first cycle of chemotherapy; T3, after the last cycle of chemotherapy and before tumor surgery. C1-C4 represent therapy cycles (3 weeks interval). **b** IFN-β levels measured in breast cancer patients of the TOP study at T1, T2 and T3 time points. **c** Comparison of median IFN-β levels at the different time points. IFN-β levels at T3 are lower compared to levels at T1 and T2. Results represent median values (bar), quantile 0.25 and quantile 0.75 (box) and extremes (lines). **d** Kaplan-Meier curves of probability of distant metastasis-free survival (DMFS) in treated breast cancer patients stratified for absence (0, black curve) or presence (1, red curve) of systematic IFN-β at T1, T2, and T3 time points, respectively. **e** Matrix analyzing potential correlations between IFN-β levels in patients’ sera (at T1, T2, T3 time points), *IFNB1* and *IRF7* expression in the tumor and four published immune/IRF7/STAT1 gene expression signatures derived from tumors at time of diagnosis

To test whether activation, or lack thereof, of the IFN-β/IFNAR1/IRF7 pathway during chemotherapy might correlate with a better, or worse outcome, respectively, we determined IFN levels in the serum of 51 ER^−^ breast cancer patients of the trial of principle (TOP) study [36] treated with neoadjuvant Epirubicin chemotherapy. Measurements were performed at time of diagnosis (T1), after the first cycle (T2), and 3–4 weeks after the last cycle (T3) of chemotherapy, before surgery (Fig. 7a). IFN-α was undetectable in most patients (data not shown). IFN-β levels were heterogeneous (from undetectable to high) across patients and within individual patients at the three time points (Fig. 7b), although average IFN-β levels at T3 were lower compared to levels detected at T1 and T2 (Fig. 7c). Patients were stratified for the presence or absence of detectable IFN-β and then analyzed for distant metastasis-free survival (DMFS). Lack of detectable IFN-β correlated with shorter DMFS at all time points with a statistically significant difference at T2 (Hazard ratio = 0.28, 95% confidence interval: 0.08–0.99, *p* = 0.049) (Fig. 7d). No significant correlations were observed between IFN-β levels at the three time points with *IRF7* and *IFNB1* gene expression and published immune gene expression signatures in the primary tumor before therapy including the IRF7 signature by Bidwell *et al*. predicting longer bone metastasis-free survival [34, 37–39]. This suggests that the evidence of the immune response, as captured by the presence of IFN-β in serum during chemotherapy, cannot be inferred from gene expression, including *IFNB1*, in the treatment-naïve primary tumors (Fig. 7e).

From these data, we conclude that the presence of circulating IFN-β in patients during neoadjuvant chemotherapy correlates with a longer DMFS, independently of *IFNB1* expression levels and immune signatures in the tumor before therapy.

## Discussion

**Fig. 8:**
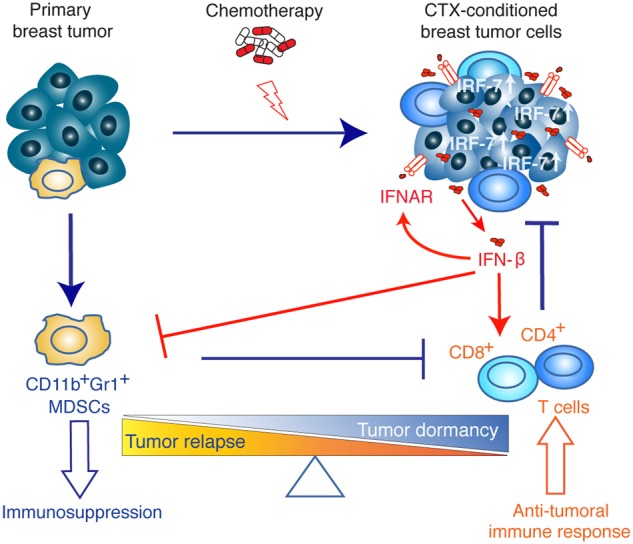
Illustrative scheme of the proposed model of chemotherapy-induced dormancy. Primary breast tumor cells escape immune elimination by inducing the expansion of immunosuppressive MDSC cells. Chemotherapy treatment of primary breast tumor induces a type I IFN response in tumor cells resulting in an autocrine and self-sustained increased IRF7 expression, IFN-β secretion triggering IFNAR signaling. IRF7/IFN-β stimulate the expansion of CD4^+^ and CD8^+^ T lymphocytes and prevent the mobilization of MDSC cells thereby switching the immune response from immunosuppressive (leading to tumor relapse) to anti-tumoral (leading to tumor dormancy)

In this work, we demonstrate that sustained activation of the IFN-β/IFNAR/IRF7 signaling axis in chemotherapy-treated ER^−^ breast cancer cells instigates immunological dormancy. Upregulated IRF7 expression in treated cancer cells is responsible for reduced cell growth, suppressed mobilization of CD11b^+^Gr1^+^ MDSCs, increased expansion of DCs, T and B lymphocytes and chemoresistance. This immune-phenotype translates into an effective anti-tumor immune response, which keeps treated cells dormant (MR20) at the primary injection site and/or decreases metastasis formation (DR500). Conversely, the inactivation of this pathway breaks tumor dormancy, engendering relapses and metastatic progression (Fig. 8). We could corroborate these experimental findings in ER^−^ breast cancer patients by showing that lack of detectable IFN-β in serum during treated Epirubicin neoadjuvant chemotherapy correlates with a shorter DMFS.

The treatment of tumor cells in vitro and the choice of drugs was guided by the following reasons: *Firstly*, to have full control of dose and timing of drug exposure*; Secondly*, to allows for complete recovery of treated and surviving cells; *Thirdly*, to limit therapy-induced mutagenesis and appearance of neo-epitopes we included Methotrexate, an antimetabolite standard of care in combination treatments in TNBC in the past, and still in used today [29, 40]; *Fourthly*, we included Doxorubicin to test a class of drug (anthracycline) widely used in TNBC also as single therapy [28, 40].

Some chemotherapies have been shown to induce the anti-tumor immune response in cancer patients [13, 41]. For example, Ma et al., reported that anthracycline-based chemotherapy induces the release of ATP by dying tumor cells, which promotes the recruitment and differentiation of antigen presenting cells in the tumor microenvironment [42]. The role of Type I IFN in promoting response to chemotherapy in tumor cells has been recently reported [24, 25]. Furthermore, expression of MHC class II antigen presentation pathways in tumor tissue is associated with better outcome in TNBC patients, consistent with a protective antitumor immune response [43]. Conversely, IFN-α deficient dendritic cells (DC) accumulating in aggressive breast cancers favor the expansion of Tregs, suggesting that IFN-α deficiency may contribute to tumor immune tolerance and poor clinical outcome [44]. These improved immediate anti-tumor effects of chemotherapy by the immune response may be explained by enhanced tumor cell death, senescence and cytotoxicity trigged by the rapid activation of type I IFN response during chemotherapy [25, 41, 45]. However, the long-term protective effects of chemotherapy as observed in (neo)adjuvant chemotherapy, and the latency (dormancy) before relapses, could not be explained by immediate anti-tumor effects but only when considering the non-linear kinetics of relapses, including in ER^−^ cancers [4, 8, 9].

Our work has two important implications. *Firstly*, it establishes chemotherapy-induced immunological dormancy in (ER^-^) breast cancer as a novel concept for how (neo)adjuvant chemotherapy may act to provide long-term survival benefits. Strikingly, in spite of the fact that immunological dormancy is a widely recognized form of dormancy [46], there is paucity of data in the literature characterizing the mechanisms involved. This may be due to the limited availability of syngeneic dormant cell lines of solid cancer and by the fact that grafting human cell lines or patients-derived xenografts into immune deficient mice excludes the possibility to study the role of the adaptive immune system. Experimental models of dormancy, however, exist in hematologic malignancies such as lymphoma and leukemia [47]. *Secondly*, it implicates type I IFN response in this effect. Previous studies based on tumor-derived IFN signatures indicate that IFN-regulated genes may correlate with favorable outcomes. We have previously reported in patients that a pretreatment STAT1 signature was associated with better prognosis in TNBC and HER2^+^ breast cancers [37] and with better response to neoadjuvant chemotherapy [48]. High IRF7 pathway activity in primary breast cancer predicted bone relapse-free survival in patients and protected against bone metastasis in mice, and treatment with IFN-α improved bone metastasis-free survival [34]. High level of IFN-β activates STAT1, STAT2 and STAT3 to facilitate cellular dormancy in tumor-repopulating melanoma cells [49]. Our work significantly extends these observations by demonstrating that cells (i.e., MR20 and DR500) that survived chemotherapy treatment maintained high expression levels of *IRF7, IRF9, STAT1* and *STAT2* even in the absence of the drugs. Consistently with these observations, in vivo IFNAR1 blockade restored a tumor-promoting immune response and broke dormancy of MR20 cells. These results extend the implication of the role of type I IFN in immunoediting in cancer [21, 22].

Although both MR20 and DR500 show similar increased immune response, they also show different in vivo phenotype. DR500 cells have a weaker activation of the IRF7/IFN response, which translates into a lesser effect on the immune response compared to MR20 tumor-bearing mice. The stronger immune response against MR20 in vivo suppressed the outgrowth both in primary site and distant organs, while the weaker activation of IRF7/IFN response in DR500 cell was only able to suppress the metastatic growth in lung consistent with the observation by Bidwell et al. [34]. In line with this notion, we observed that CD8^+^ T cells in lung of DR500-injected and 4T1-injected mice express a higher levels of cytotoxicity markers.

Although activation of the IRF7/IFN-β/IFNAR pathway contributes to cancer cell resistance against chemotherapy, the fraction of cancer cells surviving chemotherapy is small (15% after 48 h and much less at later time points) in spite of rapid (i.e., within 24 h) activation of the pathway. This implies that most cells die initially and only a few survived to develop a phenotype eliciting dormancy. The study by Weichselbaum et al. has shown that expression of IRDS is a predictive marker for chemotherapy and radiation resistance for breast cancer [26]. The author propose that the resistant cells are selected for the failure to transmit cytotoxic signal and instead results in a pro-survival signals. Based on these considerations we propose that activation of the IRF7/IFN-β/IFNAR pathway contributes to the selection of cells resistance to chemotherapy (and eliciting an immune response) but is not sufficient to protect the bulk of the cancer cells initially exposed to chemotherapy. The link between IRF7/IFN pathway and tumor resistance remains elusive at this point, which will be further studied.

In accordance with preclinical observations, we found that the absence of detectable IFN-β in the serum of TNBC patients during (i.e., T2) neoadjuvant chemotherapy treatment significantly correlated with a shorter DMFS. This also suggests that measuring immune-related parameters during (neo)adjuvant therapy may represent an additional method to obtain valuable predictive/prognostic information complementing pretreatment information. Altogether these data demonstrate that MR20-derived IFN-β promotes chemotherapy-induced dormancy. Interestingly, we observed that the spontaneously dormant D2.0R cells have an active IRF7/IFN-β axis and elicit an immune response similar to the one observed in chemotherapy-treated MR20 and DR500 cells. This raises the intriguing possibility that some forms of dormancy occurring during tumor evolution may be due to spontaneous IRF7/IFN-β axis activation.

Cancer stem cell (CSC) has been proposed to be responsible for therapy-resistance and relapse [50]. Recent study by Liu et al. [51]. showed that chemotherapy triggered tumor-independent expression of CCR2 by monocytes induces cancer stemness in both HER2^+^ and TNBC. Along with the studies by others [25, 42], the work of Liu et al. confirms that different immune cells may exerts different roles during chemotherapy, including tumor suppressive and tumor promoting effects, some of which may involve induction of stemness. Furthermore, IFN-I has recently been recently shown to control stemness in the Neu/T mouse tumor model [52]. The population of ALDH^+^ cancer stem cells was increased upon IFNAR mutation or blocking of IFN-1 [52]. From the transcriptomic data, we found that the stem cell markers Abcg2, Sca-1, and CD61 were upregulated both in MR20 and DR500, while Aldh1 and additional CSC-related markers (i.e., CD24, CD90, CD29, CD133, CD166) were not. Further studies will be necessary to better characterize MR20 and DR500 cells for functional CSC-like properties.

In conclusion, we report a novel, valuable preclinical model of chemotherapy-induced dormancy in ER^-^ breast cancer. With this model, we demonstrate the contribution of the immune system in chemotherapy-induced dormancy and identified activation of the IRF7/IFN-β/IFNAR axis as critical mechanism involved. These results further support the critical role of type I IFN response in immunoediting in cancer [21, 22]. They also identify IFN-β as potential predictive biomarker and therapeutic molecule to improve outcome of ER^-^ breast cancer patients treated with (neo)adjuvant chemotherapy.

## Material and methods

### Reagents and chemicals

Growth factor reduced Matrigel Matrix (MG) was obtained from Becton Dickinson (BD Biosciences, Basel, Switzerland). Collagenase I was purchased from Worthington (Lakewood, United Kingdom). Bovine serum albumin (BSA), crystal violet (CV) and paraformaldehyde (PFA) were obtained from Sigma-Aldrich (Buchs, Switzerland). Anti-CD4, anti-CD8 and anti-IFNAR antibodies and IgG control antibody were purchased from Bio X cell (New Hampshire, USA). Chemotherapeutic drugs Doxorubicin and Methotrexate, were generously provided by the Centre Pluridisciplinaire d’Oncologie (now Department of Oncology), University Hospital, University of Lausanne, Lausanne, Switzerland.

### Generating chemo-resistant tumor cells in vitro

10^6^ tumor cells were plated in 10 cm cell culture dish overnight before treatment. For Methotrexate selection, cells were treated with the dosage around IC85 in culture medium. The medium with Methotrexate were replaced in 2nd and 3rd day and then every 2–3 days. The treatment was continued for up to 2 weeks until single colonies of surviving cells were visible. For Doxorubicin selection, no cells survived after treatment with the dosage of IC85. The selection was performed with the dosage IC50 (0.05 µM) for one week. The surviving cells were further cultured with 0.2 µM, 0.75 µM, and 0.93 µM (500 ng/ml) consecutively for 1 week in each dosage. After selection cells were then cultured in normal medium for additional 2 weeks for recovery.

### Tumor models

4T1, MR20, DR500, D2A1, and D2.0R tumor cells (5 × 10^4^ cells for 4T1, MR20 and DR500 and 10^5^ for D2A1, D2.0R in 50 μl PBS/10% Matrigel mixture per mouse) were injected in the fourth right inguinal mammary gland of 6–7 week-old BALB/c, nude (*nu/nu)* (Harlan Laboratories, Gannat, France; Charles River Laboratories, L’Arbresle, France) and NSG (NOD SCID common gamma 2 chain deficient) female mice (University of Lausanne, Lausanne, Switzerland). Blocking IFNAR antibody was injected intraperitoneally (i.p.). We injected 10 mg/kg of antibody on days 0, 3, 8, and 15 post tumor cell injection. Prior to surgery, animals were anesthetized by an intra-peritoneal injection of ketamine (1.5 mg/kg) and xylazine (150 mg/kg) (both from Graeub, Bern, Switzerland). Tumor growth was measured twice a week with caliper and tumor volume was calculated by the equation: volume = (length × width^2^) × π/6. At the endpoint mice were sacrificed according to defined ethical criteria. Mice were killed by CO_2_ inhalation followed by neck dislocation. Tumors and lungs were resected, incubated over-night in 4% PFA and paraffin-embedded for sectioning [53]. All animal procedures were performed in accordance with the Swiss and French legislations on animal experimentation and approved by the Cantonal Veterinary Service of the Cantons Vaud and Fribourg for experiments in Lausanne and Fribourg.

### Statistical analyses (except for microarray-based gene expression analyses)

Data are presented as mean ± SEM from at least 3 independent experiments, unless otherwise indicated. Statistical comparisons were performed by an unpaired Student’s t test with a two-tailed distribution or one-way ANOVA analysis of variance with Bonferroni post-test, using Prism 7.0 GraphPad Software, Inc.

## Supplementary information

Supplementary information
