# Supplementary material for: Type I interferon/IRF7 axis instigates chemotherapy-induced immunological dormancy in breast cancer
Source: Oncogene. 2018 Dec 13;38(15):2814–29. doi: 10.1038/s41388-018-0624-2 (PMC6477891; doi:10.1038/s41388-018-0624-2)
Supplement: Supplementary file 1 — Supplementary information [file 41388_2018_624_MOESM1_ESM.pdf]

Supplementary information for

Type I interferon/IRF7 axis instigates chemotherapy-induced immunological dormancy in breast cancer

- Supplementary figures (1-9)
- Supplementary figure legends
- Supplementary table
- Supplementary methods

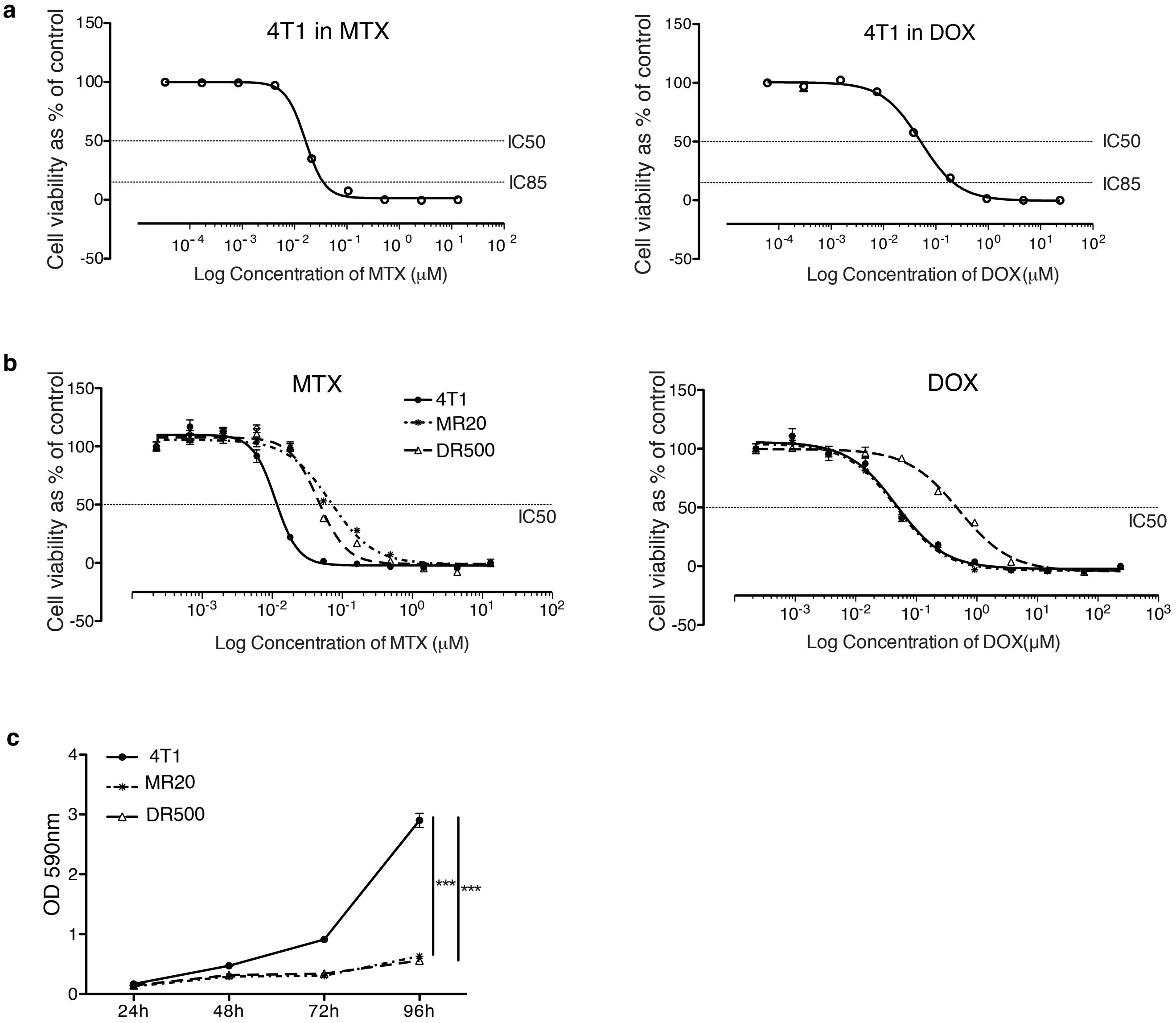

Supplementary Figure 1

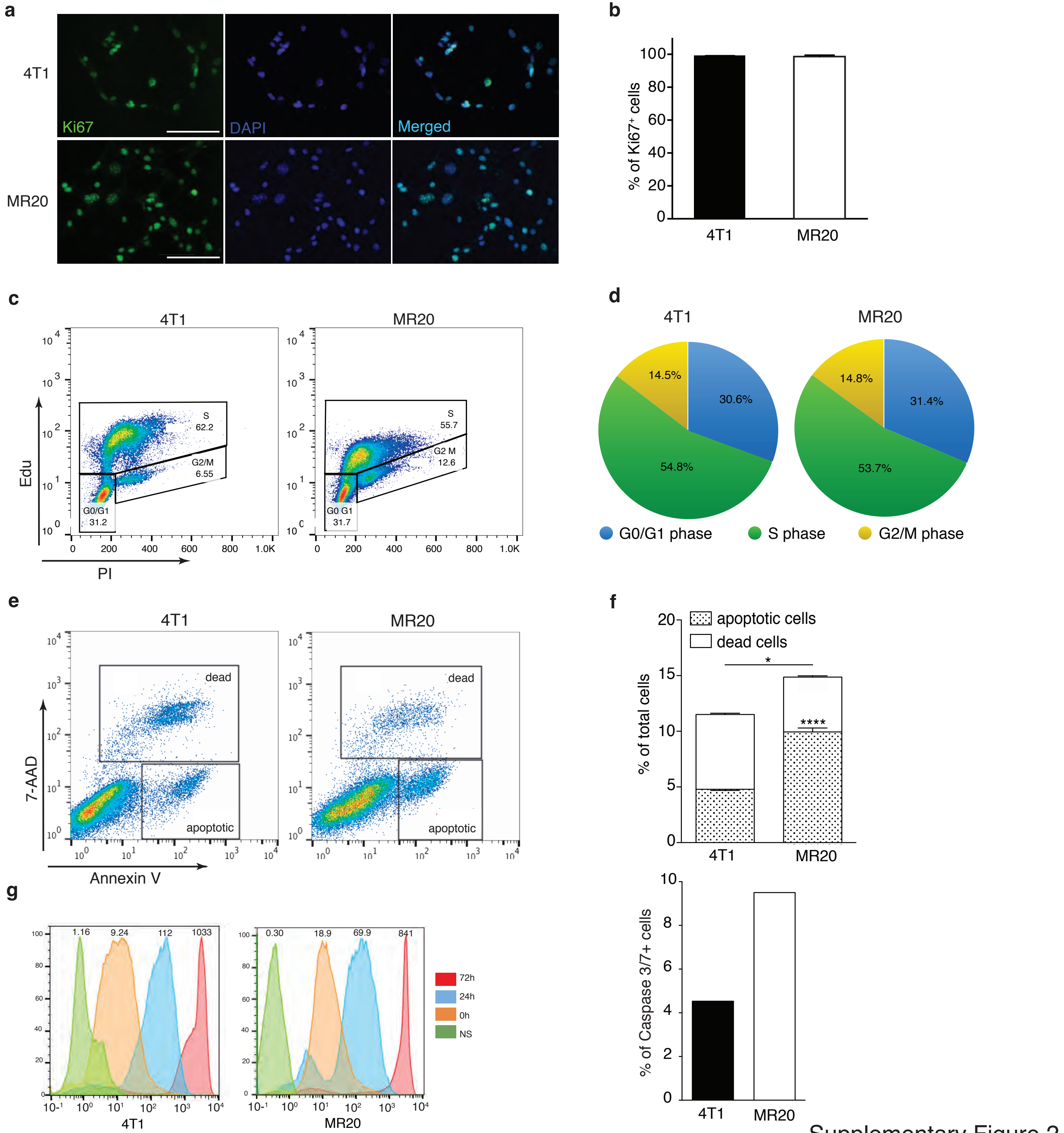

Supplementary Figure 2

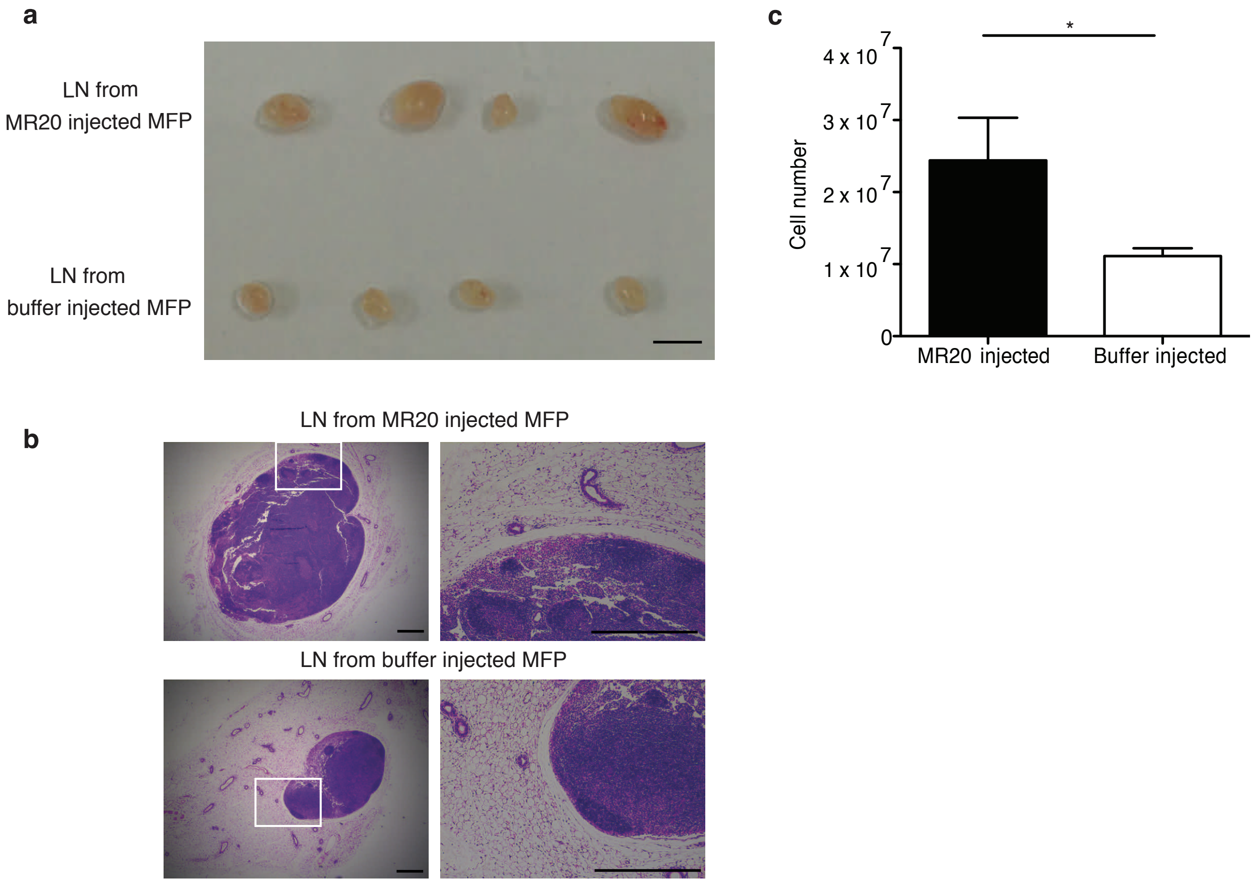

Supplementary Figure 3

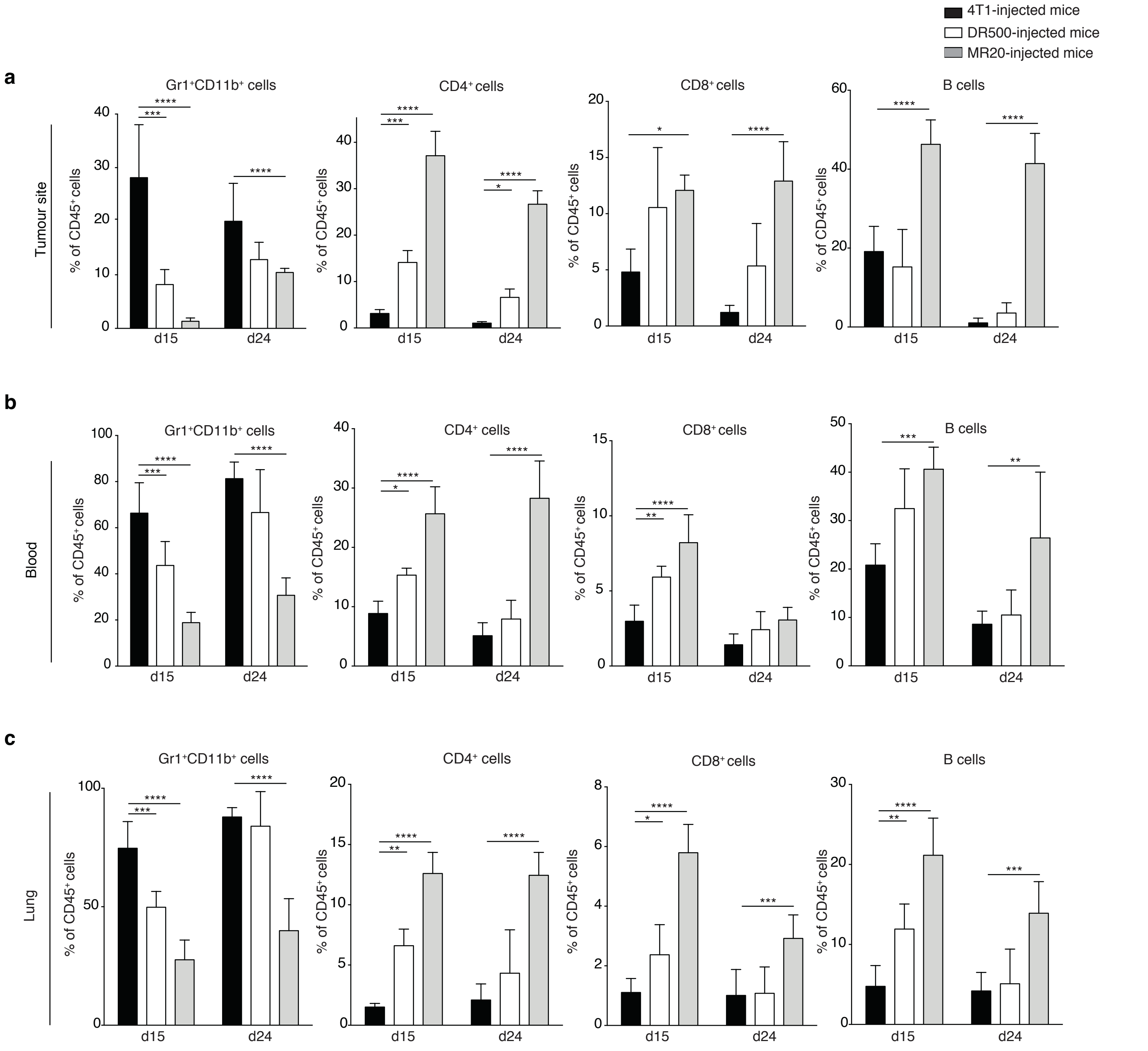

Supplementary Figure 4

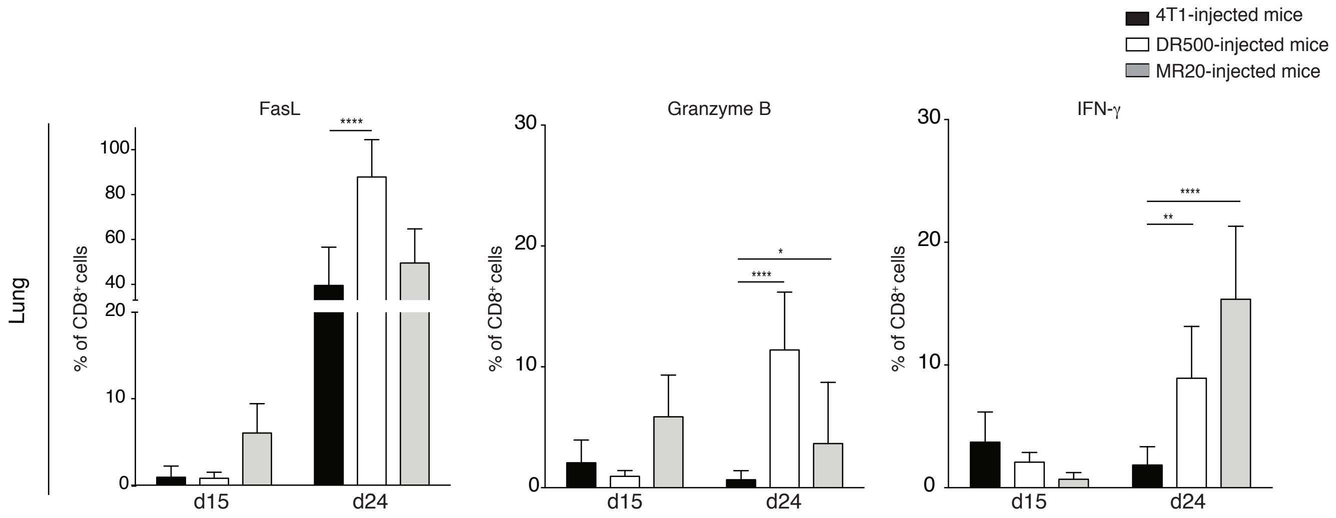

Supplementary Figure 5

a

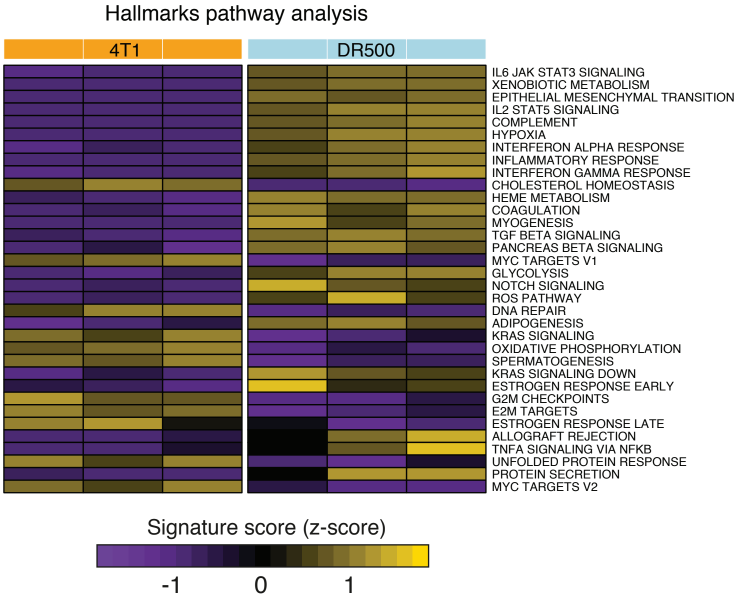

b

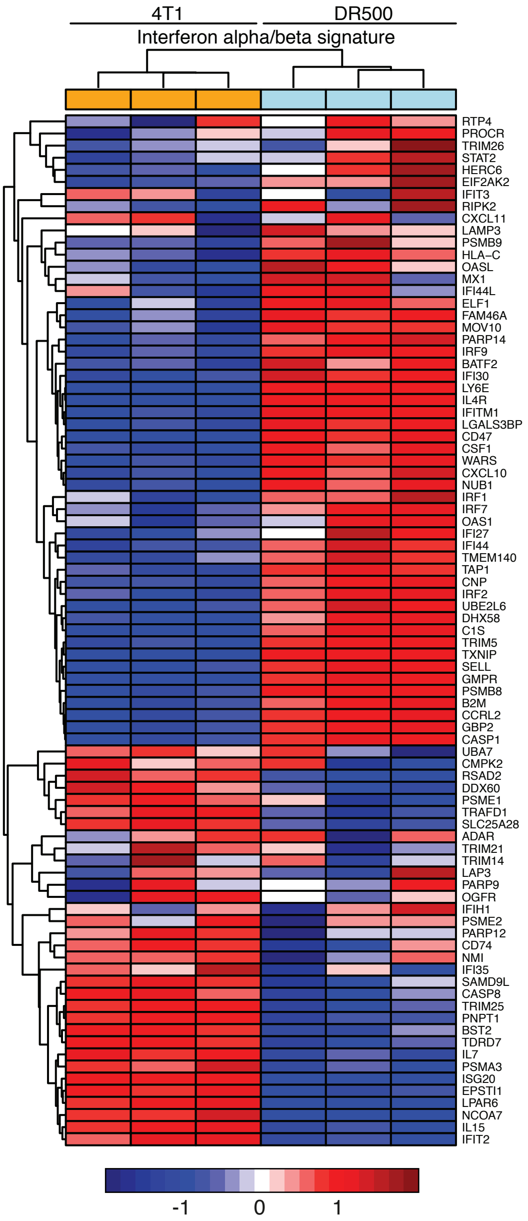

c

Enriched Transcriptional Factors

Transcriptional Factor	Fold change (DR500/4T1)	p-value
**STAT3**	3.34	1.68E-11
**AHR**	2.45	1.33E-10
**C/EBPbeta**	2.22	6.10E-09
**SMAD3**	2.21	9.87E-10
**c-Fos**	2.05	1.17E-08
**Bcl-6**	1.92	2.46E-07
**EPAS1**	1.88	9.27E-07
**NRF2**	1.81	3.86E-09
**STAT1**	1.77	6.28E-08
**MafB**	1.74	1.29E-05
**IRF7**	1.72	6.95E-05
**AP-2A**	1.69	1.84E-07
**TEF-1**	1.58	5.79E-07
**EGR1**	1.53	1.25E-05
**Fra-1**	1.52	1.25E-07

Supplementary Figure 6

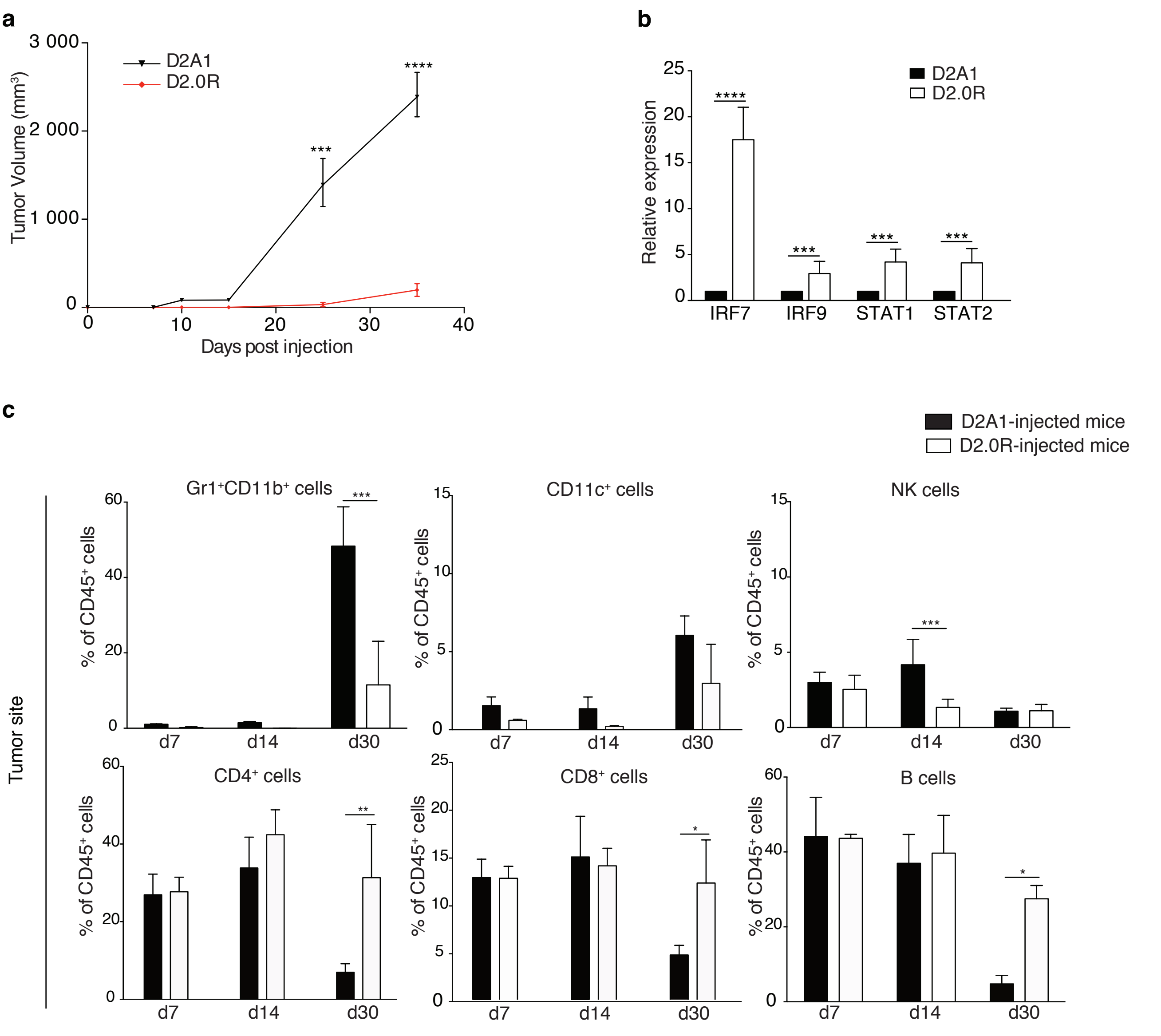

Supplementary Figure 7

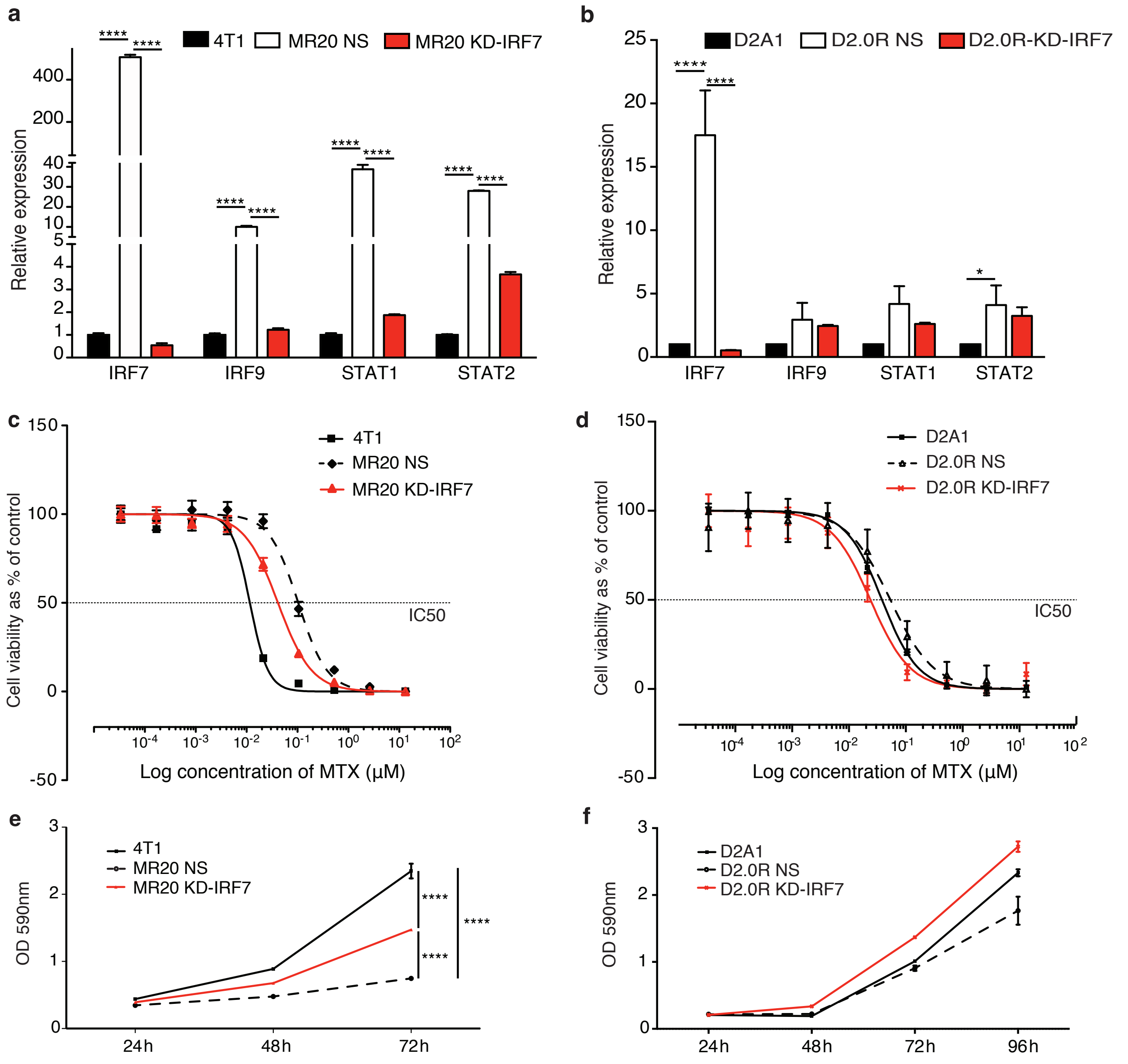

Supplementary Figure 8

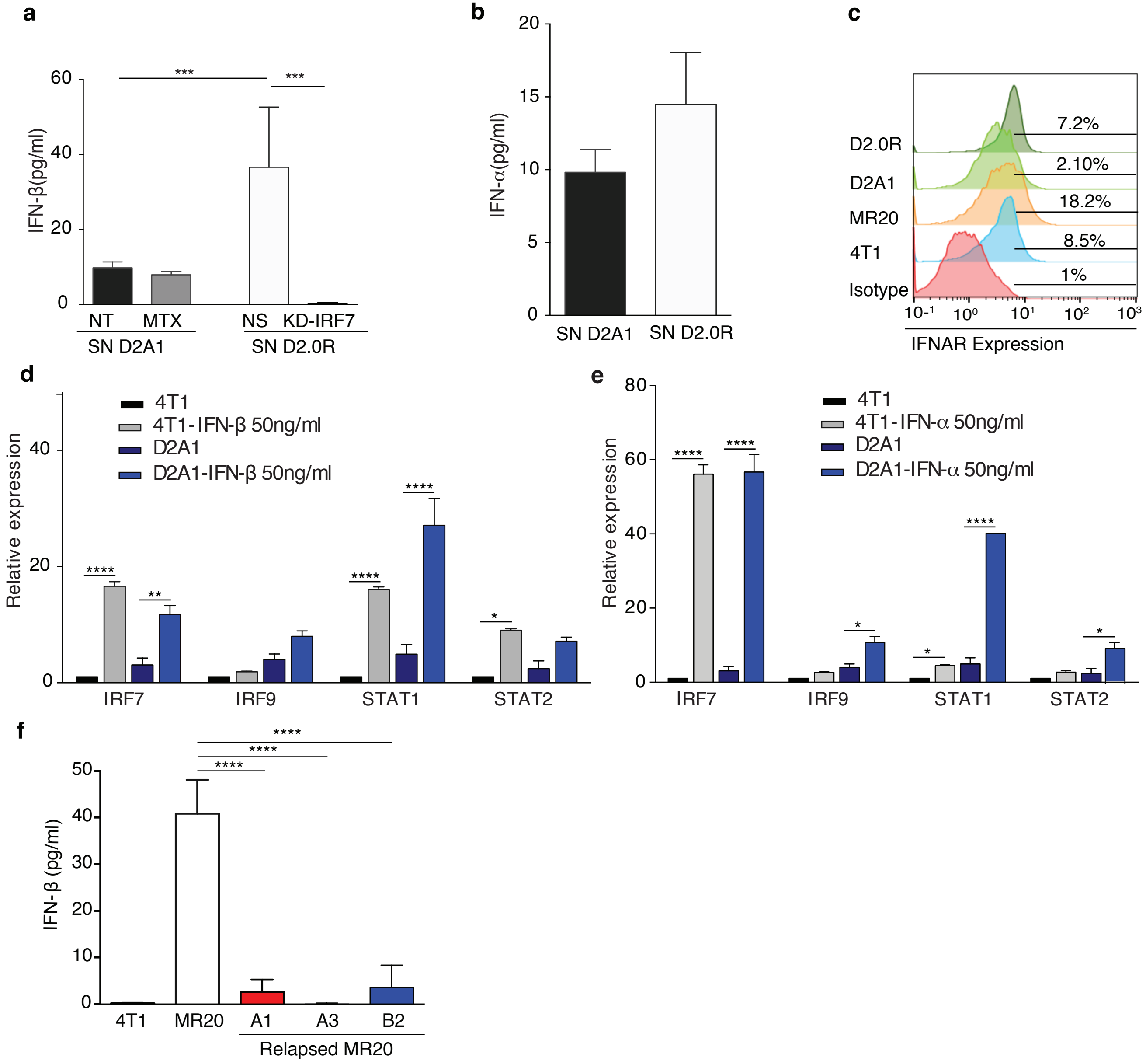

Supplementary Figure 9

Supplementary Figures legends

Supplementary Figure 1. **Chemotherapy-treated cells are more resistant to chemotherapy and form tumors growing at different rates**. **(a)** Dose dependent effect of 48 h treatment with MTX (left) or DOX (right) on 4T1 cell viability. IC50 of MTX is $1.61x10^{-2}$μM, IC50 of DOX is $5.14x10^{-2}$μM. **(b)** Dose dependent effect of 48 h treatment with MTX (left) and DOX (right) on 4T1 and drug resistant MR20 and DR500 cells' viability. MTX IC50 for MR20 and DR500 against MTX are $6.83x10^{-2}$ μM and $4.57x10^{-2}$ μM, respectively. IC50 DOX for MR20, DR500 are $4.78x10^{-2}$μM and $5.07x10^{-2}$μM, respectively. **(c)** *In vitro* growth rate of 4T1, MR20 and DR500 cells measured by crystal violet staining. Results are expressed as mean ± SEM, ***p<0.001.

Supplementary Figure 2. MR20 cells show slower proliferation and have an increased rate of apoptosis *in vitro.* **(a)** Cultured 4T1 and MR20 cells stained with the proliferation marker Ki67 (green) and the DNA staining DAPI (blue) (scale bars: 100 μm). **(b)** Quantification of the percentage of Ki67 positive cells in experiment of panel A. Both cell lines are proliferating. **(c)** Cell cycle analysis of 4T1 and MR20 by Edu/PI labelling and flow cytometry analysis. **(d)** Quantification of the percentage of 4T1 and MR20 in the G0/G1, S and G2/M phases of the cell cycle. Data are representative of three independent experiments. No significant changes in the cell cycle phases are observed. **(e)** Measurement of 4T1 and MR20 cell apoptosis by FITC-Annexin V and 7-ADD staining. **(f)** Quantification of dead and apoptotic cells as average from three independent experiments (upper panel), and quantification of cells with active Caspase 3/7 by flow cytometry analysis (lower panel). **(g)** Measurement of cell proliferation rate in 4T1 and MR20 tumor cells *in vitro* by CMFDA staining. Results are presented as histograms and MFI for t=0 h, t=24 h

and t=72 h. **(b, f)** Results are expressed as mean ± SEM of apoptotic cells, dead cells, and combination thereof. P values: *<0.05; **<0.005; ***< 0.0005, unpaired two-tailed Student's t test

Supplementary Figure 3. Enlarged draining LN in the MFP of MR20-injected mice. **(a)** Size comparison of MFP lymph nodes of MR20 cell- or saline buffer-injected BALB/c mice. **(b)** Representative images of sections of MFP draining lymph nodes from BALB/c mice injected with MR20 cells- or from saline-injected, contralateral MFP of the same mice 20 days after tumor implantation (H&E stained). **(c)** Quantification of total cell number in MFP lymph nodes of MR20 cells- or saline buffer-injected BALB/c mice. n=6 per group. *p < 0.05 (unpaired Student's *t* test).

Supplementary Figure 4. DR500 tumor cells induce a reduced mobilization of Gr1+CD11b+ cells (MDSC) and an increased accumulation of $CD4^+$, $CD8^+$ T cells compared to MR20 cells. Analysis of the immune response upon orthotopic injection of DR500, 4T1 and MR20 into BALB/c mice. Percentages of indicated immune cells in **(a)** the primary tumor/injection site, **(b)** the peripheral blood and **(c)** the lungs detected by flow cytometry analysis. Time points of analysis after tumor cells' injection (day 15 and 24) are indicated. Data are from one representative experiment. n=8/group. Results are expressed as % of $CD45^+$ cells. Data are represented as mean ± SEM. P values: * < 0.05; ** < 0.005; *** < 0.0005 by two-way ANOVA test.

Supplementary Figure 5. $CD8^+$ T cells in eth lungs of DR500- injected mice show a high level of expression of cytotoxic molecules. Analysis of cytotoxic molecule's expression in CD8+ T cells in the lung upon orthotopic DR500, 4T1 and MR20 injection into

BALB/c mice. Percentages of positive cells detected by flow cytometry analysis. Time points of analysis after tumor cells' injection (15 and 24 days) are indicated. Data are from one representative experiment. n=8/group. Results are expressed as % of $CD45^+$ cells. Data are represented as mean ± SEM. P values: * < 0.05; ** < 0.005; *** < 0.0005 by two-way ANOVA test.

Supplementary Figure 6. Gene expression analysis in 4T1 and DR500 cells. (a-b) Heat map of gene expression (a) and type I IFN signature-related gene expression (b) in 4T1 and DR500 cells color-coded based on expression levels relative to average. Blue, downregulated; red, upregulated. Results from three biological triplicates are shown.
(c) List of enriched transcription factors in DR500 vs 4T1 cells.

Supplementary Figure 7. Spontaneous dormant D2.0R cells show an up-regulation of type I IFN genes and an altered immune response. (a) Primary volume of D2A1 and D2.0R-derived tumors in the MFP of BALB/c mice. Results are expressed as mean ± SEM. n=8 per group. ***p < 0.0005. **(b)** Relative expression of IRF7, IRF9, STAT1, STAT2 transcripts in D2A1 and D2.0R tumor cells**. (c)** Percentages of indicated immune cells in the primary tumor site from D2A1- or D2.0R injected BALB/c mice detected by flow cytometry analysis. Time points of analysis (days after tumor cells' injection) are indicated. Data are from one representative experiment. n=5/group. Results are expressed as % of $CD45^+$ cells. Data are represented as mean ± SEM. P values: * < 0.05; ** < 0.005; *** < 0.0005 by two-way ANOVA test.

Supplementary Figure 8. IRF7 silencing suppresses IRF7, IRF9, STAT1 expression and reverses drug resistance and growth inhibition. (a, b) Relative expression of IRF7,

IRF9, STAT1 and STAT2 mRNA in **(a)** 4T1, MR20 non-silenced (MR20-NS), MR20 IRF7 silenced (MR20 KD-IRF7) cells and **(b)** D2A1, D2.0R non-silenced (D2.0R NS) and D2.0R IRF7 silenced (D2.0R KD-IRF7) cells determined by RT-qPCR (error bars represent SEM; p values: *<0.05; *** < 0.0005 by two-way ANOVA test). **(c, d)** Dose dependent effect of 48 h treatment with MTX on viability of **(c)** 4T1, MR20 non-silenced (MR20 NS), MR20 IRF7 silenced (MR20 KD-IRF7) cells and **(d)** D2A1, D2.0R non-silenced (D2.0R NS) and D2.0R IRF7 silenced (D2.0R KD-IRF7) cells. IC50 thresholds are indicated. **(e, f)** *In vitro* growth rate of **(e)** 4T1, MR20 non-silenced (MR20 NS), MR20 IRF7 silenced (MR20 KD-IRF7) cells and **(f)** D2A1, D2.0R non-silenced (D2.0R NS) and D2.0R IRF7 silenced (D2.0R KD-IRF7) detected by crystal violet staining. Results are expressed as mean ± SEM. n=8, p values: *** < 0.0005 by two ways ANOVA test.

Supplementary Figure 9. Type I IFN secretion, IFNAR expression and response to exogenous Type I IFN. (a) IFN-β levels in the conditioned culture supernatant of D2A1 untreated and MTX-treated tumor cells (240 nM for 24 hours) or D2.0R NS non-silenced and IRF7-silenced D2.0R (D2.0R KD-IRF7) tumor cells determined by ELISA. **(b)** IFN-α levels in the conditioned culture supernatant of D2A1 and D2.0R tumor cells determined by ELISA. **(c)** Surface expression of IFNAR1 protein in 4T1, MR20, D2A1 and D2.0R cells measured by flow cytometry, relative to isotype control staining of 4T1 cells as reference. Numbers indicate the percentage of positive cells. **(d, e).** Relative expression of *IRF7, IRF9, STAT1* and *STAT2* mRNA in 4T1 and D2A1 cells treated with (d) IFN-β (50ng/ml) or **(e)** IFN-α (50ng/ml) for 24 hours, measured by RT-qPCR. **(f)** Measurements of IFN-β levels in the supernatant of 4T1, MR20 and three MR20-derived variants (A1, A3, B2) that escaped dormancy *in vivo* by ELISA. Results are expressed as mean ± SEM. n=3.

Supplementary Table. Transcriptional profile of MR20 and DR500 cells. Gene list was generated by a class comparison between MR20 (or DR500) and 4T1 cells with False Discovery Rate (FDR)<0.05, fold change >2 (or <-2) based on microarray data analysis.

Table 1. Transcriptional profile of MR20 cells. Gene list was generated by a class comparison between MR20 and 4T1 cells with False Discovery Rate (FDR)<0.05, fold change >2 (or <-2) based on microarray data analysis.

Gene Accession	Gene Symbol	Gene Description	Fold change (MR20 vs. 4T1)	FDR
Upregulated				
NM_133871	***Ifi44***	interferon-induced protein 44	54.18	2.06E-14
NM_010501	***Ifit3***	interferon-induced protein with tetratricopeptide repeats 3	37.55	1.03E-14
NM_010927	***Nos2***	nitric oxide synthase 2, inducible	34.33	5.84E-15
NM_016850	***Irf7***	interferon regulatory factor 7	30.48	3.76E-13
NM_020557	***Cmpk2***	cytidine monophosphate (UMP-CMP) kinase 2, mitochondrial	30.17	2.56E-12
NM_001081746	***Gm7609***	predicted pseudogene 7609	29.81	1.61E-13
NM_011579	***Tgtp1***	T-cell specific GTPase 1	28.20	1.68E-14
NM_023386	***Rtp4***	receptor transporter protein 4	21.45	5.84E-15
NM_134066	***Akr1c18***	aldo-keto reductase family 1, member C18	19.94	1.09E-11
NM_001135115	***Gm12250***	predicted gene 12250	19.24	5.39E-13
NM_007695	***Chi3l1***	chitinase 3-like 1	18.80	1.32E-12
NM_021394	***Zbp1***	Z-DNA binding protein 1	18.33	5.84E-15
NM_011909	***Usp18***	ubiquitin specific peptidase 18	18.09	2.79E-14
NM_001005858	***I830012O16Rik***	RIKEN cDNA I830012O16 gene	17.71	2.65E-11
NM_033616	***Csprs***	component of Sp100-rs	16.52	2.46E-12
NM_145227	***Oas2***	2'-5' oligoadenylate synthetase 2	15.77	7.10E-12
NM_001081215	***Ddx60***	DEAD (Asp-Glu-Ala-Asp) box polypeptide 60	15.03	7.23E-14
NM_011852	***Oas1g***	2'-5' oligoadenylate synthetase 1G	14.91	9.23E-14
NM_011118	***Prl2c3***	prolactin family 2, subfamily c, member 3	14.33	4.81E-11
NM_021301	***Slc15a2***	solute carrier family 15 (H+/peptide transporter), member 2	13.59	3.39E-11
NM_145226	***Oas3***	2'-5' oligoadenylate synthetase 3	13.57	2.61E-13
NM_008620	***Gbp4***	guanylate binding protein 4	13.49	1.98E-12
NM_021384	***Rsad2***	radical S-adenosyl methionine domain containing 2	13.39	3.99E-13
NM_181852	***Prl2c5***	prolactin family 2, subfamily c, member 5	13.33	2.76E-10
NM_008522	***Ltf***	lactotransferrin	13.07	1.15E-13
NM_001174170	***Serpinb2***	serine (or cysteine) peptidase inhibitor, clade B, member 2	12.85	1.16E-11
NM_018734	***Gbp3***	guanylate binding protein 3	12.50	5.16E-13
NM_019440	***Irgm2***	immunity-related GTPase family M member 2	12.48	9.23E-14
NM_172641	***9930023K05Rik***	RIKEN cDNA 9930023K05 gene	12.09	1.64E-10
NM_011854	***Oasl2***	2'-5' oligoadenylate synthetase-like 2	11.73	1.03E-14
NM_008331	***Ifit1***	interferon-induced protein with tetratricopeptide repeats 1	11.13	1.86E-12
NM_011576	***Tfpi***	tissue factor pathway inhibitor	10.80	5.73E-13
NM_199015	***D14Ertd668e***	DNA segment, Chr 14, ERATO Doi 668, expressed	10.71	1.25E-10
NM_145211	***Oas1a***	2'-5' oligoadenylate synthetase 1A	10.35	2.61E-13
NM_009099	***Trim30a***	tripartite motif-containing 30A	10.32	9.06E-14
NM_194336	***Mpa2l***	macrophage activation 2 like	10.20	1.61E-13
BC023105	***BC023105***	cDNA sequence BC023105	10.06	5.02E-11
NM_010738	***Ly6a***	lymphocyte antigen 6 complex, locus A	9.99	2.79E-14
NM_011452	***Serpinb9b***	serine (or cysteine) peptidase inhibitor, clade B, member 9b	9.90	6.35E-12
NM_001037713	***Xaf1***	XIAP associated factor 1	9.79	2.56E-12
NM_030150	***Dhx58***	DEXH (Asp-Glu-X-His) box polypeptide 58	9.12	1.77E-12
NM_010741	***Ly6c1***	lymphocyte antigen 6 complex, locus C1	9.01	7.23E-14
NM_001039530	***Parp14***	poly (ADP-ribose) polymerase family, member 14	8.59	2.61E-13
NM_178594	***Vtcn1***	V-set domain containing T cell activation inhibitor 1	8.50	4.96E-11
NM_001177351	***AW112010***	expressed sequence AW112010	8.13	2.07E-10
NM_001045540	***Gm12185***	predicted gene 12185	8.06	3.34E-11
NM_001033767	***Gm4951***	predicted gene 4951	7.99	2.97E-10
NM_001146275	***Iigp1***	interferon inducible GTPase 1	7.98	2.76E-10
NM_015783	***Isg15***	ISG15 ubiquitin-like modifier	7.96	1.16E-12
NM_054098	***Steap4***	STEAP family member 4	7.84	9.23E-14
NM_010708	***Lgals9***	lectin, galactose binding, soluble 9	7.67	1.91E-14
NM_001099217	***Ly6c2***	lymphocyte antigen 6 complex, locus C2	7.65	9.44E-14
NM_011920	***Abcg2***	ATP-binding cassette, sub-family G (WHITE), member 2	7.17	4.48E-11
NM_017466	***Ccrl2***	chemokine (C-C motif) receptor-like 2	7.12	1.09E-11
NM_031367	***H28***	histocompatibility 28	7.02	4.43E-10
NM_001042592	***Arrdc4***	arrestin domain containing 4	6.91	1.63E-11
NM_008330	***Ifi47***	interferon gamma inducible protein 47	6.86	4.96E-11
NM_021274	***Cxcl10***	chemokine (C-X-C motif) ligand 10	6.73	1.41E-11
NM_019450	***Il1f6***	interleukin 1 family, member 6	6.66	2.57E-08
NM_011607	***Tnc***	tenascin C	6.54	8.60E-11
NM_010260	***Gbp2***	guanylate binding protein 2	6.51	3.85E-13
NM_001142959	***Bcl2l15***	BCLl2-like 15	6.50	2.18E-11
NM_010156	***Samd9l***	sterile alpha motif domain containing 9-like	6.44	1.54E-09
NM_008326	***Irgm1***	immunity-related GTPase family M member 1	6.34	1.09E-11
NM_010259	***Gbp1***	guanylate binding protein 1	6.12	2.42E-13
NM_173786	***Apol9a***	apolipoprotein L 9a	6.00	9.94E-14
NM_173743	***Apol9b***	apolipoprotein L 9b	5.81	5.07E-14
NM_145545	***Gbp6***	guanylate binding protein 6	5.76	4.73E-08
NM_028924	***Tc2n***	tandem C2 domains, nuclear	5.68	3.10E-09
NM_033217	***Ngfr***	nerve growth factor receptor (TNFR superfamily, member 16)	5.67	8.17E-10
NM_172777	***Gbp9***	guanylate-binding protein 9	5.62	3.11E-10

NM_028784	***F13a1***	coagulation factor XIII, A1 subunit	5.48	1.67E-11
NM_019949	***Ube2l6***	ubiquitin-conjugating enzyme E2L 6	5.44	7.25E-12
NM_026125	***Fam132a***	family with sequence similarity 132, member A	5.43	1.61E-13
NM_001163640	***Chn2***	chimerin (chimaerin) 2	5.38	3.56E-11
NM_026335	***Lce1h***	late cornified envelope 1H	5.33	3.34E-08
ENSMUST00000111864	***Gm5930***	predicted gene 5930	5.30	2.06E-07
NM_001004174	***AA467197***	expressed sequence AA467197	5.24	1.08E-09
NM_172689	***Ddx58***	DEAD (Asp-Glu-Ala-Asp) box polypeptide 58	5.20	1.98E-13
NM_001033122	***Cd69***	CD69 antigen	5.13	6.02E-09
NM_198095	***Bst2***	bone marrow stromal cell antigen 2	5.11	7.23E-11
NM_013653	***Ccl5***	chemokine (C-C motif) ligand 5	5.10	9.57E-13
NM_009969	***Csf2***	colony stimulating factor 2 (granulocyte-macrophage)	5.09	1.54E-09
NM_013673	***Sp100***	nuclear antigen Sp100	5.02	2.70E-11
NM_013599	***Mmp9***	matrix metallopeptidase 9	4.91	2.77E-13
NM_027835	***Ifih1***	interferon induced with helicase C domain 1	4.83	3.59E-12
NM_197944	***Hsh2d***	hematopoietic SH2 domain containing	4.78	5.33E-11
NM_001164036	***Ly6e***	lymphocyte antigen 6 complex, locus E	4.78	2.61E-13
NM_016958	***Krt14***	keratin 14	4.73	8.81E-11
NM_009291	***Stra6***	stimulated by retinoic acid gene 6	4.70	1.13E-09
NM_053141	***Pcdhb16***	protocadherin beta 16	4.65	2.44E-08
NM_019963	***Stat2***	signal transducer and activator of transcription 2	4.56	7.83E-12
NR_003508	***Mx2***	myxovirus (influenza virus) resistance 2	4.54	4.00E-10
NM_145209	***Oasl1***	2'-5' oligoadenylate synthetase-like 1	4.53	4.43E-10
NM_178767	***Tmem195***	transmembrane protein 195	4.46	2.78E-08
BC025446	***BC025446***	cDNA sequence BC025446	4.46	3.10E-08
NM_011408	***Slfn2***	schlafen 2	4.39	2.49E-11
NM_172603	***Phf11***	PHD finger protein 11	4.38	9.82E-08
NR_003507	***Oas1b***	2'-5' oligoadenylate synthetase 1B	4.38	7.55E-09
NM_009283	***Stat1***	signal transducer and activator of transcription 1	4.33	4.58E-12
NM_009256	***Serpinb9***	serine (or cysteine) peptidase inhibitor, clade B, member 9	4.28	3.52E-09
NM_013563	***Il2rg***	interleukin 2 receptor, gamma chain	4.23	1.72E-09
NM_173434	***9930111J21Rik2***	RIKEN cDNA 9930111J21 gene 2	4.20	9.71E-08
NM_181545	***Slfn8***	schlafen 8	4.16	3.11E-10
NM_001013816	***Gm5622***	predicted gene 5622	4.13	7.15E-08
NM_001009935	***Txnip***	thioredoxin interacting protein	4.12	1.56E-11
NM_175397	***Sp110***	Sp110 nuclear body protein	4.10	3.72E-10
NM_001146007	***9230105E10Rik***	RIKEN cDNA 9230105E10 gene	4.09	5.58E-06
NM_008726	***Nppb***	natriuretic peptide type B	4.07	1.01E-08
NM_007914	***Ehf***	ets homologous factor	4.06	2.94E-07
NM_022026	***Aqp9***	aquaporin 9	4.03	3.60E-09
NM_025992	***Herc6***	hect domain and RLD 6	4.01	1.98E-10
NM_177820	***Apol10b***	apolipoprotein L 10b	4.00	1.95E-07
NM_008207	***H2-T24***	histocompatibility 2, T region locus 24	3.94	9.12E-10
NM_175291	***Dock10***	dedicator of cytokinesis 10	3.92	1.85E-10
NM_013822	***Jag1***	jagged 1	3.88	2.70E-11
NM_130448	***Pcdh18***	protocadherin 18	3.85	1.47E-09
NM_029667	***Lce1i***	late cornified envelope 1I	3.80	3.72E-07
NM_009259	***Spn***	sialophorin	3.78	4.93E-10
NM_011150	***Lgals3bp***	lectin, galactoside-binding, soluble, 3 binding protein	3.78	3.10E-11
NM_019978	***Dclk1***	doublecortin-like kinase 1	3.70	5.60E-11
NM_008871	***Serpine1***	serine (or cysteine) peptidase inhibitor, clade E, member 1	3.69	1.09E-10
NM_016780	***Itgb3***	integrin beta 3	3.68	2.77E-11
AK081744	***C130073F10Rik***	RIKEN cDNA C130073F10 gene	3.67	5.77E-06
NM_172845	***Adamts4***	a disintegrin-like and metallopeptidase (reprolysin type) with thrombospondin type 1 motif, 4	3.62	4.00E-08
NM_010332	***Ednra***	endothelin receptor type A	3.61	2.19E-07
NM_172648	***Ifi205***	interferon activated gene 205	3.57	1.96E-05
NR_033483	***U90926***	cDNA sequence U90926	3.56	6.08E-08
NM_001033325	***A630095E13Rik***	RIKEN cDNA A630095E13 gene	3.52	7.90E-08
NM_008599	***Cxcl9***	chemokine (C-X-C motif) ligand 9	3.49	4.25E-08
NM_023043	***Prnd***	prion protein dublet	3.48	1.09E-11
NM_138649	***Syt17***	synaptotagmin XVII	3.48	1.03E-10
NM_139198	***Plac8***	placenta-specific 8	3.43	1.83E-08
NM_175649	***Tnfrsf26***	tumor necrosis factor receptor superfamily, member 26	3.38	3.61E-09
NM_023113	***Aspa***	aspartoacylase	3.37	3.88E-08
ENSMUST00000096862	***A530040E14Rik***	RIKEN cDNA A530040E14 gene	3.37	1.17E-07
NM_007449	***Ang2***	angiogenin, ribonuclease A family, member 2	3.37	3.07E-07
NM_001145162	***Ube2ql1***	ubiquitin-conjugating enzyme E2Q family-like 1	3.34	2.21E-09
NM_026790	***Ifi27l1***	interferon, alpha-inducible protein 27 like 1	3.33	3.39E-11
NM_013560	***Hspb1***	heat shock protein 1	3.33	5.89E-09
NM_011459	***Serpinb8***	serine (or cysteine) peptidase inhibitor, clade B, member 8	3.32	7.65E-08
NM_172659	***Slc2a6***	solute carrier family 2 (facilitated glucose transporter), member 6	3.31	1.10E-08
NM_053117	***Pard6g***	par-6 partitioning defective 6 homolog gamma (C. elegans)	3.27	5.50E-09
ENSMUST00000131035	***Rnf213***	ring finger protein 213	3.26	5.44E-09
NM_001159417	***Irf9***	interferon regulatory factor 9	3.22	6.77E-10

NM_178745	***Tmem229b***	transmembrane protein 229B	3.14	4.83E-10
NM_181547	***Nostrin***	nitric oxide synthase trafficker	3.13	3.49E-07
NM_030253	***Parp9***	poly (ADP-ribose) polymerase family, member 9	3.10	1.46E-11
NM_001033632	***Ifitm6***	interferon induced transmembrane protein 6	3.09	9.56E-09
NM_027320	***Ifi35***	interferon-induced protein 35	3.05	6.36E-09
NM_001013371	***Dtx3l***	deltex 3-like (Drosophila)	3.00	4.83E-10
NM_028019	***Rnf135***	ring finger protein 135	2.99	1.76E-08
NM_199146	***Trim30d***	tripartite motif-containing 30D	2.95	4.03E-06
NM_170599	***Igsf11***	immunoglobulin superfamily, member 11	2.95	2.56E-07
NM_019391	***Lsp1***	lymphocyte specific 1	2.95	5.69E-11
NM_001004366	***Scube3***	signal peptide, CUB domain, EGF-like 3	2.93	3.29E-08
NM_181402	***Parp11***	poly (ADP-ribose) polymerase family, member 11	2.92	1.17E-08
NM_029131	***4930503E14Rik***	RIKEN cDNA 4930503E14 gene	2.92	2.64E-06
NM_175219	***C130026I21Rik***	RIKEN cDNA C130026I21 gene	2.91	7.83E-08
NM_178804	***Slit2***	slit homolog 2 (Drosophila)	2.90	9.67E-09
NM_009140	***Cxcl2***	chemokine (C-X-C motif) ligand 2	2.89	5.03E-06
NM_009373	***Tgm2***	transglutaminase 2, C polypeptide	2.89	6.39E-09
NM_172893	***Parp12***	poly (ADP-ribose) polymerase family, member 12	2.88	1.12E-11
NM_001039647	***Gbp11***	guanylate binding protein 11	2.82	4.37E-06
NM_032399	***Gpr87***	G protein-coupled receptor 87	2.82	1.37E-08
NM_028235	***Ttc30b***	tetratricopeptide repeat domain 30B	2.80	9.42E-05
NM_019494	***Cxcl11***	chemokine (C-X-C motif) ligand 11	2.78	4.01E-06
NM_008885	***Pmp22***	peripheral myelin protein 22	2.78	4.91E-09
NM_018744	***Sema6a***	sema domain, transmembrane domain (TM), and cytoplasmic domain, (semaphorin) 6A	2.77	8.73E-09
NM_007969	***Expi***	extracellular proteinase inhibitor	2.76	3.18E-06
NM_001001495	***Tnip3***	TNFAIP3 interacting protein 3	2.75	4.03E-09
NM_008390	***Irf1***	interferon regulatory factor 1	2.73	3.11E-10
NM_001081052	***Nhs***	Nance-Horan syndrome (human)	2.71	3.05E-09
NM_010104	***Edn1***	endothelin 1	2.70	3.58E-07
NM_009841	***Cd14***	CD14 antigen	2.67	5.76E-09
NM_027158	***2310043J07Rik***	RIKEN cDNA 2310043J07 gene	2.66	2.56E-07
ENSMUST00000095985	***1700049E17Rik1***	RIKEN cDNA 1700049E17 gene, gene 1	2.64	9.88E-06
NM_001081665	***Ccdc129***	coiled-coil domain containing 129	2.64	8.85E-06
NM_024290	***Tnfrsf23***	tumor necrosis factor receptor superfamily, member 23	2.64	2.02E-09
NM_133209	***Pilrb1***	paired immunoglobin-like type 2 receptor beta 1	2.63	1.59E-08
NM_019538	***Plac1***	placental specific protein 1	2.63	1.17E-07
NM_145826	***Il17re***	interleukin 17 receptor E	2.63	5.66E-06
NM_018798	***Ubqln2***	ubiquilin 2	2.63	6.12E-09
NM_011498	***Bhlhe40***	basic helix-loop-helix family, member e40	2.62	9.20E-07
NM_009277	***Trim21***	tripartite motif-containing 21	2.62	4.79E-10
NM_011454	***Serpinb6b***	serine (or cysteine) peptidase inhibitor, clade B, member 6b	2.61	1.05E-08
BC100303	***A530040E14Rik***	RIKEN cDNA A530040E14 gene	2.61	4.37E-06
NM_026159	***Retsat***	retinol saturase (all trans retinol 13,14 reductase)	2.60	7.25E-09
NM_183162	***BC006779***	cDNA sequence BC006779	2.59	3.20E-10
NM_145562	***Parm1***	prostate androgen-regulated mucin-like protein 1	2.58	4.29E-08
NM_023716	***Tubb2b***	tubulin, beta 2B	2.57	2.24E-07
NM_029000	***Gvin1***	GTPase, very large interferon inducible 1	2.57	1.64E-05
NM_175401	***Fbxw17***	F-box and WD-40 domain protein 17	2.54	1.80E-09
NM_207237	***Man1c1***	mannosidase, alpha, class 1C, member 1	2.54	2.82E-10
NM_015784	***Postn***	periostin, osteoblast specific factor	2.53	7.30E-07
NM_178257	***Il22ra1***	interleukin 22 receptor, alpha 1	2.53	1.77E-06
NM_021515	***Ak1***	adenylate kinase 1	2.53	3.24E-09
NM_007486	***Arhgdib***	Rho, GDP dissociation inhibitor (GDI) beta	2.51	5.65E-09
NM_008489	***Lbp***	lipopolysaccharide binding protein	2.51	7.00E-05
NM_023485	***Sync***	syncoilin	2.49	1.27E-08
NM_023716	***Tubb2b***	tubulin, beta 2B	2.48	2.07E-07
NM_175366	***Mex3b***	mex3 homolog B (C. elegans)	2.47	1.41E-08
NM_019946	***Mgst1***	microsomal glutathione S-transferase 1	2.47	4.24E-09
NM_011409	***Slfn3***	schlafen 3	2.46	3.60E-09
NM_001042503	***Trim71***	tripartite motif-containing 71	2.45	8.51E-08
NM_172156	***Prl3d3***	prolactin family 3, subfamily d, member 3	2.45	1.76E-07
NM_009425	***Tnfsf10***	tumor necrosis factor (ligand) superfamily, member 10	2.45	1.08E-06
NM_013492	***Clu***	clusterin	2.44	2.76E-10
NM_029419	***Apol7a***	apolipoprotein L 7a	2.42	3.90E-06
NM_019959	***C1qtnf1***	C1q and tumor necrosis factor related protein 1	2.42	5.77E-09
NM_133720	***Cysltr2***	cysteinyl leukotriene receptor 2	2.41	5.44E-08
NM_001037987	***Edil3***	EGF-like repeats and discoidin I-like domains 3	2.41	9.32E-06
NM_153119	***Plekho2***	pleckstrin homology domain containing, family O member 2	2.41	6.51E-08
NM_001033196	***Znfx1***	zinc finger, NFX1-type containing 1	2.40	1.54E-07
NM_008470	***Krt16***	keratin 16	2.38	3.27E-07
NM_001081228	***Ttc30a2***	tetratricopeptide repeat domain 30A2	2.38	1.07E-05
NM_028071	***Cotl1***	coactosin-like 1 (Dictyostelium)	2.37	4.56E-09
NM_007899	***Ecm1***	extracellular matrix protein 1	2.36	8.07E-10
NM_013472	***Anxa6***	annexin A6	2.35	5.01E-09

NM_009853	***Cd68***	CD68 antigen	2.35	5.55E-09
NM_001013779	***Aim2***	absent in melanoma 2	2.35	0.0001574
NM_008392	***Irg1***	immunoresponsive gene 1	2.34	8.64E-07
NM_010724	***Psmb8***	proteasome (prosome, macropain) subunit, beta type 8 (large multifunctional peptidase 7)	2.34	2.27E-08
NM_145839	***Rasgef1b***	RasGEF domain family, member 1B	2.33	1.02E-05
NM_033541	***Oas1c***	2'-5' oligoadenylate synthetase 1C	2.33	5.71E-07
NM_013770	***Slc25a10***	solute carrier family 25 (mitochondrial carrier, dicarboxylate transporter), member 10	2.31	1.14E-09
NM_023044	***Slc15a3***	solute carrier family 15, member 3	2.31	9.08E-08
NM_023422	***Hist1h2bc***	histone cluster 1, H2bc	2.31	3.32E-08
NM_008557	***Fxyd3***	FXYD domain-containing ion transport regulator 3	2.30	1.25E-06
NM_183264	***5830405N20Rik***	RIKEN cDNA 5830405N20 gene	2.30	6.13E-07
NM_019737	***B4galt6***	UDP-Gal:betaGlcNAc beta 1,4-galactosyltransferase, polypeptide 6	2.29	6.41E-06
NM_001033217	***Prickle1***	prickle homolog 1 (Drosophila)	2.28	2.46E-08
NM_007616	***Cav1***	caveolin 1, caveolae protein	2.28	2.17E-06
NM_177469	***Gpr123***	G protein-coupled receptor 123	2.28	6.18E-06
NM_018884	***Pdzrn3***	PDZ domain containing RING finger 3	2.27	2.26E-09
NM_145434	***Nr1d1***	nuclear receptor subfamily 1, group D, member 1	2.26	3.94E-09
NM_030711	***Erap1***	endoplasmic reticulum aminopeptidase 1	2.26	1.14E-09
NM_001136076	***P4ha2***	procollagen-proline, 2-oxoglutarate 4-dioxygenase (proline 4-hydroxylase), alpha II polypeptid	2.26	2.54E-08
NM_001033040	***Gm166***	predicted gene 166	2.25	4.59E-07
NM_013670	***Snrpn***	small nuclear ribonucleoprotein N	2.25	1.05E-07
NM_001034859	***Gm4841***	predicted gene 4841	2.24	0.0001419
NM_013585	***Psmb9***	proteasome (prosome, macropain) subunit, beta type 9 (large multifunctional peptidase 2)	2.24	1.21E-06
NM_029509	***Gbp8***	guanylate-binding protein 8	2.23	1.31E-05
NM_011390	***Slc12a7***	solute carrier family 12, member 7	2.23	2.93E-08
NM_026619	***Gsto2***	glutathione S-transferase omega 2	2.22	1.61E-06
NM_008864	***Prl3d1***	prolactin family 3, subfamily d, member 1	2.22	1.30E-08
NM_013683	***Tap1***	transporter 1, ATP-binding cassette, sub-family B (MDR/TAP)	2.22	7.75E-08
NM_027897	***Rhpn2***	rhophilin, Rho GTPase binding protein 2	2.22	1.76E-07
NM_013640	***Psmb10***	proteasome (prosome, macropain) subunit, beta type 10	2.22	3.79E-09
NM_213615	***A530032D15Rik***	RIKEN cDNA A530032D15Rik gene	2.21	3.78E-06
NM_001033167	***Slc22a23***	solute carrier family 22, member 23	2.21	7.55E-09
NM_008969	***Ptgs1***	prostaglandin-endoperoxide synthase 1	2.21	7.00E-10
NR_030719	***Gm8979***	very large inducible GTPase 1 pseudogene	2.21	0.0001852
NM_009807	***Casp1***	caspase 1	2.20	1.80E-06
NM_153510	***Pilra***	paired immunoglobin-like type 2 receptor alpha	2.20	3.37E-05
NM_011252	***Rbmx***	RNA binding motif protein, X chromosome	2.19	7.81E-08
NM_138753	***Hexim1***	hexamethylene bis-acetamide inducible 1	2.19	3.41E-08
NM_001083628	***Greb1l***	growth regulation by estrogen in breast cancer-like	2.19	6.64E-05
NM_028665	***Ankrd42***	ankyrin repeat domain 42	2.19	1.03E-06
NM_027249	***Tlcd2***	TLC domain containing 2	2.18	8.61E-08
NM_009712	***Arsb***	arylsulfatase B	2.18	2.09E-08
NM_153560	***Fam102a***	family with sequence similarity 102, member A	2.18	1.76E-07
NM_008046	***Fst***	follistatin	2.18	3.34E-07
NM_080451	***Synpo2***	synaptopodin 2	2.18	9.03E-07
NM_018754	***Sfn***	stratifin	2.17	6.20E-05
NM_130869	***Nobox***	NOBOX oogenesis homeobox	2.17	1.35E-05
NM_177388	***Slc41a2***	solute carrier family 41, member 2	2.16	4.00E-08
NM_015759	***Fgd3***	FYVE, RhoGEF and PH domain containing 3	2.16	5.52E-08
NM_144830	***Tmem106a***	transmembrane protein 106A	2.16	1.17E-07
NM_010495	***Id1***	inhibitor of DNA binding 1	2.16	5.29E-09
NM_008634	***Mtap1b***	microtubule-associated protein 1B	2.15	4.45E-08
NM_001141949	***Nmi***	N-myc (and STAT) interactor	2.15	9.99E-08
NM_001001326	***St5***	suppression of tumorigenicity 5	2.15	1.76E-08
NM_027853	***Mettl7b***	methyltransferase like 7B	2.15	1.64E-06
NM_011973	***Stk30***	serine/threonine kinase 30	2.15	6.21E-07
NM_001168504	***Pla2g4c***	phospholipase A2, group IVC (cytosolic, calcium-independent)	2.15	1.99E-05
NM_015760	***Nox4***	NADPH oxidase 4	2.15	8.80E-07
NM_009335	***Tcfap2c***	transcription factor AP-2, gamma	2.14	6.19E-06
NM_011332	***Ccl17***	chemokine (C-C motif) ligand 17	2.14	1.24E-07
NM_172796	***Slfn9***	schlafen 9	2.14	6.49E-07
NM_152804	***Plk2***	polo-like kinase 2 (Drosophila)	2.14	4.14E-08
NM_009142	***Cx3cl1***	chemokine (C-X3-C motif) ligand 1	2.14	1.86E-08
NM_177390	***Myo1d***	myosin ID	2.13	5.65E-09
NM_177368	***Tmtc2***	transmembrane and tetratricopeptide repeat containing 2	2.13	9.62E-05
NM_145825	***Cetn4***	centrin 4	2.13	0.0003665
NM_001024932	***Pilrb2***	paired immunoglobin-like type 2 receptor beta 2	2.13	9.65E-07
NM_027144	***Arhgef12***	Rho guanine nucleotide exchange factor (GEF) 12	2.12	8.61E-05
NM_007436	***Aldh3a1***	aldehyde dehydrogenase family 3, subfamily A1	2.12	4.34E-08
NM_173379	***Leprel1***	leprecan-like 1	2.12	4.00E-08
NM_001038587	***Adar***	adenosine deaminase, RNA-specific	2.11	6.84E-09
NM_001081185	***Flnc***	filamin C, gamma	2.11	4.06E-08
NM_010493	***Icam1***	intercellular adhesion molecule 1	2.09	1.40E-05
NM_008327	***Ifi202b***	interferon activated gene 202B	2.09	8.48E-06

NM_011337	***Ccl3***	chemokine (C-C motif) ligand 3	2.09	3.65E-05
NM_018827	***Crlf1***	cytokine receptor-like factor 1	2.09	7.70E-07
NM_011163	***Eif2ak2***	eukaryotic translation initiation factor 2-alpha kinase 2	2.09	3.84E-07
NM_025413	***Lce1g***	late cornified envelope 1G	2.07	1.40E-05
NM_001034858	***Armc2***	armadillo repeat containing 2	2.07	0.0007705
NM_009964	***Cryab***	crystallin, alpha B	2.07	1.76E-08
NM_025385	***Prr13***	proline rich 13	2.06	1.23E-08
NM_001013817	***Sp140***	Sp140 nuclear body protein	2.06	9.03E-06
NM_001143848	***Pde2a***	phosphodiesterase 2A, cGMP-stimulated	2.06	1.28E-05
NM_172477	***Dennd2a***	DENN/MADD domain containing 2A	2.06	2.46E-06
ENSMUST00000090166	***Adh6b***	alcohol dehydrogenase 6B (class V)	2.06	7.57E-06
NM_153412	***Phldb2***	pleckstrin homology-like domain, family B, member 2	2.05	1.83E-08
NM_007930	***Enc1***	ectodermal-neural cortex 1	2.05	4.25E-08
NM_019835	***B4galt5***	UDP-Gal:betaGlcNAc beta 1,4-galactosyltransferase, polypeptide 5	2.04	1.90E-09
NM_009504	***Vdr***	vitamin D receptor	2.04	8.27E-08
NM_021491	***Smpd3***	sphingomyelin phosphodiesterase 3, neutral	2.04	8.68E-06
NM_001033433	***Tmem102***	transmembrane protein 102	2.03	1.36E-07
NM_177905	***Piwil4***	piwi-like homolog 4 (Drosophila)	2.03	3.25E-08
NM_011375	***St3gal5***	ST3 beta-galactoside alpha-2,3-sialyltransferase 5	2.03	3.11E-07
NM_026837	***Tmem53***	transmembrane protein 53	2.03	1.36E-05
NM_001163470	***Trafd1***	TRAF type zinc finger domain containing 1	2.02	3.32E-08
NM_019807	***Acpp***	acid phosphatase, prostate	2.02	5.17E-07
NM_009264	***Sprr1a***	small proline-rich protein 1A	2.02	2.55E-05
NM_008827	***Pgf***	placental growth factor	2.02	2.27E-05
NM_181585	***Pik3r3***	phosphatidylinositol 3 kinase, regulatory subunit, polypeptide 3 (p55)	2.01	3.56E-07
NM_029341	***Capsl***	calcyphosine-like	2.01	9.80E-07
NM_144807	***Chpt1***	choline phosphotransferase 1	2.01	2.02E-09
NM_172414	***Fam164c***	family with sequence similarity 164, member C	2.00	5.80E-05
NM_133754	***Fblim1***	filamin binding LIM protein 1	2.00	1.13E-08
NM_134250	***Havcr2***	hepatitis A virus cellular receptor 2	2.00	8.80E-07
NM_001164612	***Atp13a4***	ATPase type 13A4	2.00	1.19E-06
Downregulated				
NR_028572	***Snora43***	small nucleolar RNA, H/ACA box 43	-2.00	4.51E-06
NM_025462	***Ece2***	endothelin converting enzyme 2	-2.00	8.64E-07
NM_146054	***Fermt2***	fermitin family homolog 2 (Drosophila)	-2.00	1.02E-08
NM_001172475	***Sphk1***	sphingosine kinase 1	-2.01	2.03E-08
NM_013602	***Mt1***	metallothionein 1	-2.01	1.33E-08
NM_001033711	***Evi2a***	ecotropic viral integration site 2a	-2.01	2.21E-07
NR_028079	***Snora3***	small nucleolar RNA, H/ACA box 3	-2.01	5.71E-05
NM_029777	***Rhbdd1***	rhomboid domain containing 1	-2.01	2.03E-08
NM_028789	***Unkl***	unkempt-like (Drosophila)	-2.02	7.27E-09
NM_028460	***Pear1***	platelet endothelial aggregation receptor 1	-2.02	2.82E-07
NM_018760	***Slc4a4***	solute carrier family 4 (anion exchanger), member 4	-2.02	2.96E-07
NM_026066	***Cmtm5***	CKLF-like MARVEL transmembrane domain containing 5	-2.03	8.26E-07
NM_172709	***Otop1***	otopetrin 1	-2.03	4.59E-06
NM_144515	***Zfp52***	zinc finger protein 52	-2.03	2.25E-05
NM_025366	***Chchd1***	coiled-coil-helix-coiled-coil-helix domain containing 1	-2.03	8.11E-06
NM_023065	***Ifi30***	interferon gamma inducible protein 30	-2.03	7.95E-08
NR_029412	***Snora16a***	small nucleolar RNA, H/ACA box 16A	-2.03	0.0001792
NM_022890	***Cldn12***	claudin 12	-2.03	2.71E-09
NM_078478	***Ghitm***	growth hormone inducible transmembrane protein	-2.04	1.32E-09
NM_029578	***Tgds***	TDP-glucose 4,6-dehydratase	-2.04	9.41E-07
NM_016782	***Cntnap1***	contactin associated protein-like 1	-2.04	3.23E-06
NM_019969	***Plag1***	pleiomorphic adenoma gene 1	-2.04	1.80E-05
NR_029736	***Mir18***	microRNA 18	-2.05	0.0004182
NR_028095	***Rabggtb***	RAB geranylgeranyl transferase, b subunit	-2.05	8.55E-08
BC032970	***2810026P18Rik***	RIKEN cDNA 2810026P18 gene	-2.05	1.11E-06
NM_027617	***Spata1***	spermatogenesis associated 1	-2.05	8.09E-06
NM_001146012	***R3hcc1***	R3H domain and coiled-coil containing 1	-2.05	9.55E-08
NM_008926	***Prkg2***	protein kinase, cGMP-dependent, type II	-2.06	0.0004159
NM_001042611	***Cp***	ceruloplasmin	-2.06	7.01E-09
NM_030889	***Sorcs2***	sortilin-related VPS10 domain containing receptor 2	-2.07	3.65E-05
NM_001025586	***Nr2c2ap***	nuclear receptor 2C2-associated protein	-2.07	1.29E-06
NM_007786	***Csn3***	casein kappa	-2.07	4.76E-05
NM_001162977	***Megf6***	multiple EGF-like-domains 6	-2.07	4.23E-06
NM_023292	***Pus3***	pseudouridine synthase 3	-2.07	1.41E-08
NM_009128	***Scd2***	stearoyl-Coenzyme A desaturase 2	-2.07	8.92E-10
NM_015728	***Slc33a1***	solute carrier family 33 (acetyl-CoA transporter), member 1	-2.07	3.40E-07
NM_027289	***Nt5dc2***	5'-nucleotidase domain containing 2	-2.08	6.58E-09
NM_013737	***Pla2g7***	phospholipase A2, group VII (platelet-activating factor acetylhydrolase, plasma)	-2.08	3.52E-09
NM_001081396	***Wdr67***	WD repeat domain 67	-2.08	1.08E-09
NM_026331	***Slc25a37***	solute carrier family 25, member 37	-2.08	1.32E-09
NM_178688	***Ablim1***	actin-binding LIM protein 1	-2.08	8.55E-08
NM_024250	***Phf10***	PHD finger protein 10	-2.08	9.78E-08

NM_001033171	***Klrg2***	killer cell lectin-like receptor subfamily G, member 2	-2.08	3.59E-06
NM_008837	***Pick1***	protein interacting with C kinase 1	-2.08	5.00E-08
NM_008675	***Nbl1***	neuroblastoma, suppression of tumorigenicity 1	-2.08	2.54E-07
NM_133992	***Pan2***	PAN2 polyA specific ribonuclease subunit homolog (S. cerevisiae)	-2.09	3.19E-09
NM_001013384	***Podnl1***	podocan-like 1	-2.09	4.10E-07
NM_001099314	***Msmp***	microseminoprotein, prostate associated	-2.10	1.21E-06
NM_025697	***6330409N04Rik***	RIKEN cDNA 6330409N04 gene	-2.10	1.96E-08
NM_029020	***Abcb8***	ATP-binding cassette, sub-family B (MDR/TAP), member 8	-2.11	1.33E-08
NM_009713	***Arsa***	arylsulfatase A	-2.11	1.37E-07
NR_004418	***Rnu73b***	U73B small nuclear RNA	-2.11	0.0001475
NM_030110	***Efha2***	EF-hand domain family, member A2	-2.11	0.0003914
NM_019979	***Selk***	selenoprotein K	-2.11	5.08E-08
NM_028567	***1700094D03Rik***	RIKEN cDNA 1700094D03 gene	-2.11	5.96E-07
NM_028759	***Dcaf6***	DDB1 and CUL4 associated factor 6	-2.11	3.72E-07
NM_025512	***Zfand1***	zinc finger, AN1-type domain 1	-2.11	1.85E-06
NM_001081332	***Slc9a5***	solute carrier family 9 (sodium/hydrogen exchanger), member 5	-2.12	4.14E-08
NM_017479	***Myst4***	MYST histone acetyltransferase monocytic leukemia 4	-2.12	8.20E-06
NM_001039514	***Dhps***	deoxyhypusine synthase	-2.13	2.23E-07
NM_010231	***Fmo1***	flavin containing monooxygenase 1	-2.13	2.88E-05
NM_027077	***1700016C15Rik***	RIKEN cDNA 1700016C15 gene	-2.13	1.10E-05
BC145212	***3110057O12Rik***	RIKEN cDNA 3110057O12 gene	-2.13	5.03E-06
NM_030693	***Atf5***	activating transcription factor 5	-2.14	5.17E-08
NM_177586	***Eif5a2***	eukaryotic translation initiation factor 5A2	-2.14	1.86E-07
NM_027982	***Ppm1j***	protein phosphatase 1J	-2.14	1.54E-07
NM_009621	***Adamts1***	a disintegrin-like and metallopeptidase (reprolysin type) with thrombospondin type 1 motif, 1	-2.14	3.73E-08
NR_028513	***Snora73b***	small nucleolar RNA, H/ACA box 73b	-2.14	0.0019134
NM_172779	***Ddx26b***	DEAD/H (Asp-Glu-Ala-Asp/His) box polypeptide 26B	-2.15	1.73E-07
NM_028000	***Ppapdc1b***	phosphatidic acid phosphatase type 2 domain containing 1B	-2.15	8.39E-10
NM_054048	***Rcor2***	REST corepressor 2	-2.16	2.36E-06
NM_001162909	***Ncrna00085***	non-protein coding RNA 85	-2.16	2.77E-07
NM_009996	***Cyp24a1***	cytochrome P450, family 24, subfamily a, polypeptide 1	-2.17	1.96E-06
NM_001033210	***Pls1***	plastin 1 (I-isoform)	-2.17	4.70E-08
NM_144844	***Pcca***	propionyl-Coenzyme A carboxylase, alpha polypeptide	-2.17	5.17E-07
NM_026840	***Pdgfrl***	platelet-derived growth factor receptor-like	-2.17	1.84E-08
NM_018857	***Msln***	mesothelin	-2.18	6.39E-10
NM_145953	***Cth***	cystathionase (cystathionine gamma-lyase)	-2.18	1.42E-09
NM_133900	***Psph***	phosphoserine phosphatase	-2.19	7.83E-08
BC062906	***A330021E22Rik***	RIKEN cDNA A330021E22 gene	-2.20	5.38E-05
NM_145394	***Slc44a3***	solute carrier family 44, member 3	-2.20	6.53E-06
NM_177420	***Psat1***	phosphoserine aminotransferase 1	-2.20	5.01E-10
NM_001099632	***Rnf39***	ring finger protein 39	-2.20	3.96E-09
NM_007472	***Aqp1***	aquaporin 1	-2.21	5.14E-07
NR_029382	***Mir17hg***	MIR17 host gene 1 (non-protein coding)	-2.21	7.74E-06
NM_153807	***Acsf2***	acyl-CoA synthetase family member 2	-2.21	1.52E-06
BC056964	***Taf1d***	TATA box binding protein (Tbp)-associated factor, RNA polymerase I, D	-2.21	0.0007946
NM_022018	***Fam129a***	family with sequence similarity 129, member A	-2.22	1.37E-05
BC027506	***2010012O05Rik***	RIKEN cDNA 2010012O05 gene	-2.22	5.15E-08
NR_003368	***Pvt1***	plasmacytoma variant translocation 1	-2.22	1.39E-06
NM_001013745	***Zfp57***	zinc finger protein 57	-2.23	1.08E-07
NM_133943	***Hsd3b7***	hydroxy-delta-5-steroid dehydrogenase, 3 beta- and steroid delta-isomerase 7	-2.23	9.10E-09
NM_009051	***Rex2***	reduced expression 2	-2.24	2.23E-08
NM_001008501	***Zfp760***	zinc finger protein 760	-2.24	2.43E-06
NM_001038703	***Gpr146***	G protein-coupled receptor 146	-2.25	3.20E-07
NM_001163471	***Hectd2***	HECT domain containing 2	-2.26	3.71E-05
NM_001146180	***Mtss1***	metastasis suppressor 1	-2.26	7.15E-09
NM_207213	***Snx25***	sorting nexin 25	-2.26	1.13E-06
NM_012009	***Sh2d1b1***	SH2 domain protein 1B1	-2.27	2.94E-05
NM_019425	***Gnpnat1***	glucosamine-phosphate N-acetyltransferase 1	-2.29	1.23E-07
NM_028048	***Slc25a35***	solute carrier family 25, member 35	-2.29	7.15E-08
NM_013490	***Chka***	choline kinase alpha	-2.29	2.25E-10
NM_019581	***Gtpbp2***	GTP binding protein 2	-2.29	7.09E-08
NM_019637	***Styx***	serine/threonine/tyrosine interaction protein	-2.30	3.29E-08
NM_011020	***Hspa4l***	heat shock protein 4 like	-2.30	1.31E-07
NM_011950	***Mapk13***	mitogen-activated protein kinase 13	-2.30	4.43E-10
NM_134079	***Adk***	adenosine kinase	-2.30	1.84E-08
NM_024188	***Oxct1***	3-oxoacid CoA transferase 1	-2.30	9.02E-11
NM_008730	***Nptx1***	neuronal pentraxin 1	-2.30	3.78E-09
NM_178920	***Mal2***	mal, T-cell differentiation protein 2	-2.31	2.37E-08
NM_172753	***Csgalnact1***	chondroitin sulfate N-acetylgalactosaminyltransferase 1	-2.31	7.35E-07
NM_008681	***Ndrg1***	N-myc downstream regulated gene 1	-2.32	3.10E-09
NM_001163288	***Susd1***	sushi domain containing 1	-2.32	6.63E-08
NM_146106	***Lyplal1***	lysophospholipase-like 1	-2.32	4.99E-06
NM_010363	***Gstz1***	glutathione transferase zeta 1 (maleylacetoacetate isomerase)	-2.32	2.14E-06
NM_011309	***S100a1***	S100 calcium binding protein A1	-2.33	5.54E-08

NM_033564	***Mpv17l***	Mpv17 transgene, kidney disease mutant-like	-2.34	3.10E-08
NM_021509	***Moxd1***	monooxygenase, DBH-like 1	-2.37	5.61E-08
NM_027777	***Pex1***	peroxisomal biogenesis factor 1	-2.37	3.98E-05
NM_001163741	***1110021J02Rik***	RIKEN cDNA 1110021J02 gene	-2.38	8.19E-08
NM_026819	***Dhrs1***	dehydrogenase/reductase (SDR family) member 1	-2.38	1.77E-10
NM_022424	***Fndc4***	fibronectin type III domain containing 4	-2.39	8.99E-09
NM_008209	***Mr1***	major histocompatibility complex, class I-related	-2.39	3.62E-10
NM_001002897	***Atg9b***	ATG9 autophagy related 9 homolog B (S. cerevisiae)	-2.40	4.10E-06
NM_001033258	***D10Bwg1379e***	DNA segment, Chr 10, Brigham & Women's Genetics 1379 expressed	-2.41	1.16E-07
NM_175236	***Adhfe1***	alcohol dehydrogenase, iron containing, 1	-2.42	5.39E-07
NM_009994	***Cyp1b1***	cytochrome P450, family 1, subfamily b, polypeptide 1	-2.42	7.92E-10
NM_009657	***Aldoc***	aldolase C, fructose-bisphosphate	-2.44	5.51E-07
NM_001170694	***Rcbtb2***	regulator of chromosome condensation (RCC1) and BTB (POZ) domain containing protein 2	-2.45	2.34E-08
NM_008018	***Sh3pxd2a***	SH3 and PX domains 2A	-2.45	1.94E-07
NM_010324	***Got1***	glutamate oxaloacetate transaminase 1, soluble	-2.45	4.43E-10
NM_007963	***Mecom***	MDS1 and EVI1 complex locus	-2.45	7.70E-08
NM_008113	***Arhgdig***	Rho GDP dissociation inhibitor (GDI) gamma	-2.45	1.31E-07
NM_013473	***Anxa8***	annexin A8	-2.45	7.48E-09
NM_007557	***Bmp7***	bone morphogenetic protein 7	-2.46	5.13E-09
NM_001033598	***Acsl6***	acyl-CoA synthetase long-chain family member 6	-2.46	1.79E-09
BC138272	***1700106N22Rik***	RIKEN cDNA 1700106N22 gene	-2.46	2.24E-05
NM_173763	***Ccbl2***	cysteine conjugate-beta lyase 2	-2.47	1.56E-09
NM_001081191	***Eml5***	echinoderm microtubule associated protein like 5	-2.48	5.20E-09
NM_015744	***Enpp2***	ectonucleotide pyrophosphatase/phosphodiesterase 2	-2.49	1.54E-08
AF357393	***Rps12***	ribosomal protein S12	-2.49	3.42E-06
NM_147155	***Tagap1***	T-cell activation GTPase activating protein 1	-2.50	8.59E-08
NM_053195	***Slc24a3***	solute carrier family 24 (sodium/potassium/calcium exchanger), member 3	-2.50	1.33E-08
NR_028574	***Snhg8***	small nucleolar RNA host gene 8	-2.50	7.33E-05
NM_133679	***Cryzl1***	crystallin, zeta (quinone reductase)-like 1	-2.51	4.20E-09
NR_002840	***Gas5***	growth arrest specific 5	-2.51	8.78E-10
NM_026931	***1810011O10Rik***	RIKEN cDNA 1810011O10 gene	-2.52	7.24E-07
NM_181409	***Mtmr11***	myotubularin related protein 11	-2.53	2.33E-07
NM_145466	***A2ld1***	AIG2-like domain 1	-2.54	2.76E-10
NM_019454	***Dll4***	delta-like 4 (Drosophila)	-2.55	2.23E-07
NM_023645	***Kdelc1***	KDEL (Lys-Asp-Glu-Leu) containing 1	-2.56	7.36E-08
NM_172524	***Nipal4***	NIPA-like domain containing 4	-2.60	8.52E-09
NR_004445	***Snord22***	small nucleolar RNA, C/D box 22	-2.61	1.95E-06
NM_008439	***Khk***	ketohexokinase	-2.63	1.71E-08
NM_130878	***Cdhr1***	cadherin-related family member 1	-2.63	3.90E-08
NM_177235	***Bend6***	BEN domain containing 6	-2.64	8.46E-08
NM_009829	***Ccnd2***	cyclin D2	-2.68	2.39E-08
NM_010797	***Mid1***	midline 1	-2.68	4.10E-07
NM_019698	***Aldh18a1***	aldehyde dehydrogenase 18 family, member A1	-2.69	1.98E-10
NM_177260	***Tmem154***	transmembrane protein 154	-2.71	5.58E-07
NM_011075	***Abcb1b***	ATP-binding cassette, sub-family B (MDR/TAP), member 1B	-2.73	3.34E-08
NM_145133	***Tifa***	TRAF-interacting protein with forkhead-associated domain	-2.75	1.06E-06
NM_146074	***Tfb1m***	transcription factor B1, mitochondrial	-2.76	3.61E-09
NM_153543	***Aldh1l2***	aldehyde dehydrogenase 1 family, member L2	-2.79	2.86E-08
NM_011896	***Spry1***	sprouty homolog 1 (Drosophila)	-2.81	3.60E-09
NM_175358	***Zdhhc15***	zinc finger, DHHC domain containing 15	-2.81	1.16E-09
NM_198657	***Gm5148***	predicted gene 5148	-2.82	1.05E-06
NM_134092	***Mtbp***	Mdm2, transformed 3T3 cell double minute p53 binding protein	-2.83	1.17E-10
NR_028527	***Snord52***	small nucleolar RNA, C/D box 52	-2.85	2.53E-07
NM_146236	***Tceal1***	transcription elongation factor A (SII)-like 1	-2.86	2.91E-05
NM_139292	***Reep6***	receptor accessory protein 6	-2.87	1.67E-07
NM_172514	***Tmem71***	transmembrane protein 71	-2.88	2.08E-08
BC132471	***Gm129***	predicted gene 129	-2.91	7.76E-07
ENSMUST00000084298	***LOC100038746***	hypothetical LOC100038746	-2.91	1.54E-07
NM_153526	***Insig1***	insulin induced gene 1	-2.91	6.96E-10
AK051045	***Snhg1***	small nucleolar RNA host gene (non-protein coding) 1	-2.93	2.45E-05
NM_009705	***Arg2***	arginase type II	-2.93	8.34E-11
ENSMUST00000056686	***2210011C24Rik***	RIKEN cDNA 2210011C24 gene	-2.94	2.63E-08
NM_019730	***Nme3***	non-metastatic cells 3, protein expressed in	-2.95	1.18E-09
NM_178381	***Ano9***	anoctamin 9	-2.96	6.17E-08
NM_175329	***Chchd10***	coiled-coil-helix-coiled-coil-helix domain containing 10	-2.96	2.02E-09
NM_144795	***Pycr1***	pyrroline-5-carboxylate reductase 1	-3.03	1.26E-08
NM_001081005	***1500012F01Rik***	RIKEN cDNA 1500012F01 gene	-3.06	4.83E-10
NM_001081413	***Unc13b***	unc-13 homolog B (C. elegans)	-3.08	4.57E-08
NM_013847	***Gcat***	glycine C-acetyltransferase (2-amino-3-ketobutyrate-coenzyme A ligase)	-3.09	4.31E-10
NM_199449	***Zhx2***	zinc fingers and homeoboxes 2	-3.09	5.61E-09
NM_029840	***2610029I01Rik***	RIKEN cDNA 2610029I01 gene	-3.09	1.21E-06
NM_011173	***Pros1***	protein S (alpha)	-3.09	1.33E-07
NM_026770	***Cgref1***	cell growth regulator with EF hand domain 1	-3.11	2.66E-09
NM_028994	***Pck2***	phosphoenolpyruvate carboxykinase 2 (mitochondrial)	-3.12	1.09E-10

NM_013543	***H2-Ke6***	H2-K region expressed gene 6	-3.13	9.59E-10
NM_029293	***Phpt1***	phosphohistidine phosphatase 1	-3.15	8.36E-10
NM_031843	***Dpp7***	dipeptidylpeptidase 7	-3.18	1.71E-09
NM_173866	***Gpt2***	glutamic pyruvate transaminase (alanine aminotransferase) 2	-3.20	5.14E-07
NM_026517	***Rpl22l1***	ribosomal protein L22 like 1	-3.22	4.25E-06
NM_027032	***Pacrg***	PARK2 co-regulated	-3.27	4.36E-08
NM_027294	***Cmtm8***	CKLF-like MARVEL transmembrane domain containing 8	-3.29	3.67E-07
NM_010136	***Eomes***	eomesodermin homolog (Xenopus laevis)	-3.29	5.08E-09
NM_175151	***Tatdn1***	TatD DNase domain containing 1	-3.31	1.85E-10
NM_178396	***Car12***	carbonic anyhydrase 12	-3.33	1.34E-10
NM_011725	***Xlr***	X-linked lymphocyte-regulated complex	-3.40	3.29E-05
NM_010022	***Dbt***	dihydrolipoamide branched chain transacylase E2	-3.43	4.43E-10
NM_001001488	***Atp8b1***	ATPase, class I, type 8B, member 1	-3.58	2.31E-08
ENSMUST00000029268	***1810062G17Rik***	RIKEN cDNA 1810062G17 gene	-3.75	3.74E-08
NM_001164201	***Lass3***	LAG1 homolog, ceramide synthase 3	-3.81	3.11E-10
NM_011851	***Nt5e***	5' nucleotidase, ecto	-4.00	1.09E-11
NM_008862	***Pkia***	protein kinase inhibitor, alpha	-4.02	2.03E-07
NM_030004	***Cryl1***	crystallin, lambda 1	-4.13	3.59E-12
NM_008492	***Ldhb***	lactate dehydrogenase B	-4.15	4.45E-09
NM_172880	***Tmprss11e***	transmembrane protease, serine 11e	-4.16	3.11E-10
NM_009721	***Atp1b1***	ATPase, Na+/K+ transporting, beta 1 polypeptide	-4.17	9.51E-11
NM_008144	***Bscl2***	Bernardinelli-Seip congenital lipodystrophy 2 homolog (human)	-4.29	2.73E-11
NM_198620	***Rundc3b***	RUN domain containing 3B	-4.37	1.53E-08
NM_020293	***Cldn9***	claudin 9	-4.42	6.69E-09
NM_134090	***Kdelr3***	KDEL (Lys-Asp-Glu-Leu) endoplasmic reticulum protein retention receptor 3	-4.54	7.83E-12
NM_016899	***Rab25***	RAB25, member RAS oncogene family	-4.56	5.61E-10
NM_001164311	***Loxl4***	lysyl oxidase-like 4	-4.62	1.54E-07
NM_001111119	***Ccnb1ip1***	cyclin B1 interacting protein 1	-4.91	6.87E-11
NM_024263	***Mxra8***	matrix-remodelling associated 8	-4.94	1.56E-09
NM_138653	***Bspry***	B-box and SPRY domain containing	-5.27	2.10E-10
NM_008009	***Fgfbp1***	fibroblast growth factor binding protein 1	-5.53	1.01E-08
NM_008813	***Enpp1***	ectonucleotide pyrophosphatase/phosphodiesterase 1	-5.61	1.79E-11
NM_026416	***S100a16***	S100 calcium binding protein A16	-5.77	2.31E-08
NM_020577	***As3mt***	arsenic (+3 oxidation state) methyltransferase	-6.10	2.80E-09
NM_178936	***Tmem56***	transmembrane protein 56	-6.18	7.23E-11
NM_009127	***Scd1***	stearoyl-Coenzyme A desaturase 1	-6.25	1.12E-12
NM_025961	***Gatm***	glycine amidinotransferase (L-arginine:glycine amidinotransferase)	-6.44	2.07E-13
NM_016707	***Bcl11a***	B-cell CLL/lymphoma 11A (zinc finger protein)	-6.74	9.14E-11
NM_010421	***Hexa***	hexosaminidase A	-6.82	1.09E-11
NM_139305	***Car9***	carbonic anhydrase 9	-7.88	1.12E-11
NM_029556	***Clybl***	citrate lyase beta like	-7.94	9.02E-11
NM_018755	***Pgcp***	plasma glutamate carboxypeptidase	-9.64	2.38E-09
NM_181588	***Cmbl***	carboxymethylenebutenolidase-like (Pseudomonas)	-10.90	2.49E-11
NM_145078	***2610305D13Rik***	RIKEN cDNA 2610305D13 gene	-10.93	4.78E-10

Table 2. Transcriptional profile of DR500 cells. Gene list was generated by a class comparison between DR500 and 4T1 cells with False Discovery Rate (FDR)<0.05, fold change >2 (or <-2) based on microarray data analysis.

Gene Accession	Gene Symbol	Gene Description	Fold change (DR500 vs. 4T1)	FDR
Upregulated				
NM_007976	***F5***	coagulation factor V	67.31	1.50E-13
NM_011347	***Selp***	selectin, platelet	50.50	5.06E-14
NM_011315	***Saa3***	serum amyloid A 3	49.33	2.59E-12
NM_029796	***Lrg1***	leucine-rich alpha-2-glycoprotein 1	37.42	2.61E-13
NM_054098	***Steap4***	STEAP family member 4	35.00	7.16E-16
NM_011314	***Saa2***	serum amyloid A 2	34.33	2.87E-11
NM_013584	***Lifr***	leukemia inhibitory factor receptor	27.27	6.50E-13
NM_011118	***Prl2c3***	prolactin family 2, subfamily c, member 3	26.49	2.40E-12
NM_181390	***Mustn1***	musculoskeletal, embryonic nuclear protein 1	23.43	2.61E-13
NM_181852	***Prl2c5***	prolactin family 2, subfamily c, member 5	22.78	1.53E-11
NM_010741	***Ly6c1***	lymphocyte antigen 6 complex, locus C1	20.81	2.02E-15
NM_007651	***Cd53***	CD53 antigen	20.33	1.55E-12
NR_033556	***A630010A05Rik***	RIKEN cDNA A630010A05 gene	18.13	2.36E-06
NM_009888	***Cfh***	complement component factor h	17.41	3.51E-14
NM_008489	***Lbp***	lipopolysaccharide binding protein	17.33	3.03E-10
BC025446	***BC025446***	cDNA sequence BC025446	16.42	1.43E-11
NM_001099217	***Ly6c2***	lymphocyte antigen 6 complex, locus C2	16.26	3.45E-15
NM_133859	***Olfml3***	olfactomedin-like 3	15.69	1.63E-14
NM_001033632	***Ifitm6***	interferon induced transmembrane protein 6	15.35	3.31E-13
NM_010738	***Ly6a***	lymphocyte antigen 6 complex, locus A	14.90	4.13E-15
NM_013532	***Lilrb4***	leukocyte immunoglobulin-like receptor, subfamily B, member 4	14.90	1.41E-10
NM_001081656	***Neurl1B***	neuralized homolog 1B (Drosophila)	14.15	4.90E-13
NM_028784	***F13a1***	coagulation factor XIII, A1 subunit	13.75	1.03E-13
NM_021412	***Mmp19***	matrix metallopeptidase 19	13.19	1.06E-11
NM_010180	***Fbln1***	fibulin 1	13.04	5.41E-13
NM_134066	***Akr1c18***	aldo-keto reductase family 1, member C18	12.92	2.82E-11
NM_028903	***Scara5***	scavenger receptor class A, member 5 (putative)	12.66	3.50E-12
NM_001009935	***Txnip***	thioredoxin interacting protein	12.51	2.70E-14
NM_009640	***Angpt1***	angiopoietin 1	11.39	3.64E-11
NM_018782	***Calcrl***	calcitonin receptor-like	11.37	1.06E-11
NM_010332	***Ednra***	endothelin receptor type A	11.31	9.85E-11
NM_145509	***5430435G22Rik***	RIKEN cDNA 5430435G22 gene	10.91	1.89E-12
NM_009416	***Tpm2***	tropomyosin 2, beta	10.57	9.62E-14
NM_007449	***Ang2***	angiogenin, ribonuclease A family, member 2	10.37	1.17E-10
NM_007472	***Aqp1***	aquaporin 1	10.00	2.40E-12
NM_153422	***Pde5a***	phosphodiesterase 5A, cGMP-specific	9.86	8.08E-14
NM_015749	***Tcn2***	transcobalamin 2	9.66	1.93E-11
NM_028072	***Sulf2***	sulfatase 2	9.32	3.98E-12
NM_007436	***Aldh3a1***	aldehyde dehydrogenase family 3, subfamily A1	9.14	1.94E-13
NM_011019	***Osmr***	oncostatin M receptor	8.90	7.63E-13
NM_021515	***Ak1***	adenylate kinase 1	8.88	1.61E-13
NM_011435	***Sod3***	superoxide dismutase 3, extracellular	8.80	2.02E-10
NM_001122899	***Lepr***	leptin receptor	8.05	1.39E-09
NM_011340	***Serpinf1***	serine (or cysteine) peptidase inhibitor, clade F, member 1	7.92	5.56E-13
NM_008177	***Grpr***	gastrin releasing peptide receptor	7.81	1.32E-09
NM_011612	***Tnfrsf9***	tumor necrosis factor receptor superfamily, member 9	7.78	2.53E-13
NM_181588	***Cmbl***	carboxymethylenebutenolidase-like (Pseudomonas)	7.73	6.23E-11
NM_025622	***Lgals2***	lectin, galactose-binding, soluble 2	7.64	1.79E-12
NM_008555	***Masp1***	mannan-binding lectin serine peptidase 1	7.63	3.38E-13
NM_011723	***Xdh***	xanthine dehydrogenase	7.59	9.64E-14
NM_019634	***Tspan7***	tetraspanin 7	7.48	1.09E-09
NM_008344	***Igfbp6***	insulin-like growth factor binding protein 6	7.39	1.48E-12
NM_173379	***Leprel1***	leprecan-like 1	7.26	5.01E-13
NM_007901	***S1pr1***	sphingosine-1-phosphate receptor 1	7.26	4.80E-10
NM_011452	***Serpinb9b***	serine (or cysteine) peptidase inhibitor, clade B, member 9b	7.18	1.54E-11
NM_001033344	***Dusp27***	dual specificity phosphatase 27 (putative)	7.01	1.27E-09
NM_013566	***Itgb7***	integrin beta 7	6.75	4.05E-11
NM_016873	***Wisp2***	WNT1 inducible signaling pathway protein 2	6.71	2.18E-09
NM_013691	***Thbs3***	thrombospondin 3	6.53	7.03E-12
NM_013869	***Tnfrsf19***	tumor necrosis factor receptor superfamily, member 19	6.45	7.77E-13
NM_011520	***Sdc3***	syndecan 3	6.42	9.62E-14
NM_016846	***Rgl1***	ral guanine nucleotide dissociation stimulator,-like 1	6.31	4.64E-12
NM_019733	***Rbpms***	RNA binding protein gene with multiple splicing	6.30	1.00E-12
NM_026907	***Sectm1b***	secreted and transmembrane 1B	6.23	5.56E-11
NM_001163640	***Chn2***	chimerin (chimaerin) 2	6.17	7.49E-12
NM_009116	***Prrx2***	paired related homeobox 2	6.13	6.88E-10
NM_010189	***Fcgrt***	Fc receptor, IgG, alpha chain transporter	6.10	2.60E-11
NM_008147	***Gp49a***	glycoprotein 49 A	6.01	1.06E-09
NM_010358	***Gstm1***	glutathione S-transferase, mu 1	5.97	5.55E-12
NM_011346	***Sell***	selectin, lymphocyte	5.97	1.17E-09
NM_010222	***Fkbp7***	FK506 binding protein 7	5.90	1.00E-12

NM_183168	***P2ry6***	pyrimidinergic receptor P2Y, G-protein coupled, 6	5.87	3.63E-10
ENSMUST00000023246	***Ly6g***	lymphocyte antigen 6 complex, locus G	5.69	9.33E-09
NM_013605	***Muc1***	mucin 1, transmembrane	5.67	3.98E-12
NM_178907	***Mapkapk3***	mitogen-activated protein kinase-activated protein kinase 3	5.62	6.19E-13
NM_008239	***Foxq1***	forkhead box Q1	5.62	4.42E-11
NM_007899	***Ecm1***	extracellular matrix protein 1	5.61	2.36E-13
NM_146126	***Sord***	sorbitol dehydrogenase	5.59	4.16E-12
NM_009255	***Serpine2***	serine (or cysteine) peptidase inhibitor, clade E, member 2	5.55	1.23E-12
NM_027455	***Qpct***	glutaminyl-peptide cyclotransferase (glutaminyl cyclase)	5.50	1.54E-08
NM_007707	***Socs3***	suppressor of cytokine signaling 3	5.45	1.73E-12
NM_025288	***Stfa3***	stefin A3	5.42	6.28E-08
NM_021507	***Sqrdl***	sulfide quinone reductase-like (yeast)	5.40	3.45E-09
NM_021564	***Fetub***	fetuin beta	5.33	2.88E-08
NM_016780	***Itgb3***	integrin beta 3	5.19	1.05E-12
NM_178111	***Trp53inp2***	transformation related protein 53 inducible nuclear protein 2	5.15	1.27E-11
NM_011173	***Pros1***	protein S (alpha)	5.13	1.09E-09
NM_027711	***Iqgap2***	IQ motif containing GTPase activating protein 2	5.04	1.70E-11
NM_010225	***Foxf2***	forkhead box F2	5.02	1.04E-09
NM_177231	***Arrb1***	arrestin, beta 1	5.00	1.03E-10
NM_008587	***Mertk***	c-mer proto-oncogene tyrosine kinase	4.99	1.50E-10
NM_010927	***Nos2***	nitric oxide synthase 2, inducible	4.98	3.98E-12
NM_009853	***Cd68***	CD68 antigen	4.93	2.55E-12
NM_010216	***Figf***	c-fos induced growth factor	4.90	8.27E-11
NM_008486	***Anpep***	alanyl (membrane) aminopeptidase	4.86	7.52E-10
NM_022420	***Gprc5b***	G protein-coupled receptor, family C, group 5, member B	4.82	2.17E-10
NM_010930	***Nov***	nephroblastoma overexpressed gene	4.82	1.92E-11
NM_001008700	***Il4ra***	interleukin 4 receptor, alpha	4.78	6.77E-13
NM_177343	***Camk1d***	calcium/calmodulin-dependent protein kinase ID	4.76	1.69E-11
NM_008714	***Notch1***	Notch gene homolog 1 (Drosophila)	4.72	2.36E-10
NM_175367	***Ston2***	stonin 2	4.70	1.27E-11
NM_033602	***Peli2***	pellino 2	4.63	2.73E-10
NM_001012401	***Hspb6***	heat shock protein, alpha-crystallin-related, B6	4.59	7.63E-13
NM_197996	***Tspan15***	tetraspanin 15	4.57	2.16E-10
NM_007431	***Alpl***	alkaline phosphatase, liver/bone/kidney	4.57	1.10E-11
NM_008181	***Gsta1***	glutathione S-transferase, alpha 1 (Ya)	4.55	2.13E-09
NM_007730	***Col12a1***	collagen, type XII, alpha 1	4.54	2.37E-12
NM_029306	***1700012B09Rik***	RIKEN cDNA 1700012B09 gene	4.49	2.81E-10
NM_026376	***Plxnd1***	plexin D1	4.49	2.95E-11
NM_054041	***Antxr1***	anthrax toxin receptor 1	4.49	1.61E-13
NM_175263	***Notum***	notum pectinacetylesterase homolog (Drosophila)	4.49	2.08E-10
NM_009373	***Tgm2***	transglutaminase 2, C polypeptide	4.49	5.89E-11
NM_001081178	***Gpr116***	G protein-coupled receptor 116	4.46	8.87E-11
NM_001042592	***Arrdc4***	arrestin domain containing 4	4.46	1.30E-10
NM_008597	***Mgp***	matrix Gla protein	4.43	3.13E-11
NM_199241	***Sema6d***	sema domain, transmembrane domain (TM), and cytoplasmic domain, (semaphorin) 6D	4.43	2.14E-11
NM_026743	***Tspan11***	tetraspanin 11	4.42	1.94E-09
NM_176963	***Galm***	galactose mutarotase	4.41	5.63E-09
NM_019789	***Kcnip3***	Kv channel interacting protein 3, calsenilin	4.41	9.40E-11
NM_019753	***Cdh17***	cadherin 17	4.38	1.02E-10
NM_008706	***Nqo1***	NAD(P)H dehydrogenase, quinone 1	4.38	5.66E-11
NM_010276	***Gem***	GTP binding protein (gene overexpressed in skeletal muscle)	4.35	1.01E-11
NM_019441	***Ppt2***	palmitoyl-protein thioesterase 2	4.35	2.77E-11
NM_029537	***Tmem98***	transmembrane protein 98	4.32	2.62E-10
NM_011925	***Cd97***	CD97 antigen	4.23	8.75E-11
NM_013454	***Abca1***	ATP-binding cassette, sub-family A (ABC1), member 1	4.23	2.09E-10
NM_029837	***Mpped2***	metallophosphoesterase domain containing 2	4.22	6.70E-08
NM_009285	***Stc1***	stanniocalcin 1	4.22	9.18E-11
NM_144938	***C1s***	complement component 1, s subcomponent	4.21	7.03E-08
NM_025508	***Gmpr***	guanosine monophosphate reductase	4.17	2.18E-09
NM_010708	***Lgals9***	lectin, galactose binding, soluble 9	4.16	4.28E-13
NM_028351	***Rspo3***	R-spondin 3 homolog (Xenopus laevis)	4.13	1.26E-08
NM_008969	***Ptgs1***	prostaglandin-endoperoxide synthase 1	4.10	6.45E-13
NM_134250	***Havcr2***	hepatitis A virus cellular receptor 2	4.09	2.04E-10
NM_009610	***Actg2***	actin, gamma 2, smooth muscle, enteric	4.06	1.90E-10
NM_007646	***Cd38***	CD38 antigen	4.04	3.07E-12
NM_011122	***Plod1***	procollagen-lysine, 2-oxoglutarate 5-dioxygenase 1	4.03	2.49E-11
NM_007887	***Dub1***	deubiquitinating enzyme 1	3.99	2.28E-07
NM_009807	***Casp1***	caspase 1	3.98	2.24E-09
NM_001013779	***Aim2***	absent in melanoma 2	3.97	8.73E-07
NM_148941	***Elovl4***	elongation of very long chain fatty acids (FEN1/Elo2, SUR4/Elo3, yeast)-like 4	3.93	4.53E-07
NM_010145	***Ephx1***	epoxide hydrolase 1, microsomal	3.91	6.38E-10
NM_017370	***Hp***	haptoglobin	3.85	3.27E-08
NM_032393	***Mtap1a***	microtubule-associated protein 1 A	3.85	1.39E-08
NM_013737	***Pla2g7***	phospholipase A2, group VII (platelet-activating factor acetylhydrolase, plasma)	3.84	1.89E-12

NM_026820	***Ifitm1***	interferon induced transmembrane protein 1	3.82	2.21E-07
NM_007539	***Bdkrb1***	bradykinin receptor, beta 1	3.80	1.31E-08
NM_011345	***Sele***	selectin, endothelial cell	3.79	2.14E-09
NM_008856	***Prkch***	protein kinase C, eta	3.78	7.53E-12
NM_053110	***Gpnmb***	glycoprotein (transmembrane) nmb	3.78	6.71E-12
NM_007602	***Capn5***	calpain 5	3.76	6.33E-11
NM_019946	***Mgst1***	microsomal glutathione S-transferase 1	3.75	2.66E-11
NM_001099298	***Scn2a1***	sodium channel, voltage-gated, type II, alpha 1	3.74	3.97E-07
NM_010231	***Fmo1***	flavin containing monooxygenase 1	3.72	5.72E-08
NM_029771	***Gpr30***	G protein-coupled receptor 30	3.72	7.34E-11
NM_031843	***Dpp7***	dipeptidylpeptidase 7	3.71	1.81E-10
NM_010359	***Gstm3***	glutathione S-transferase, mu 3	3.66	2.76E-10
NM_153573	***Fkbp14***	FK506 binding protein 14	3.63	3.24E-08
NM_023118	***Dab2***	disabled homolog 2 (Drosophila)	3.62	3.68E-11
NM_023422	***Hist1h2bc***	histone cluster 1, H2bc	3.61	1.31E-10
NM_011116	***Pld3***	phospholipase D family, member 3	3.61	1.19E-11
NM_007786	***Csn3***	casein kappa	3.60	9.03E-08
NM_007759	***Crabp2***	cellular retinoic acid binding protein II	3.57	1.22E-07
NM_008350	***Il11***	interleukin 11	3.55	4.00E-09
NM_008437	***Napsa***	napsin A aspartic peptidase	3.52	9.18E-11
NM_010600	***Kcnh1***	potassium voltage-gated channel, subfamily H (eag-related), member 1	3.51	6.06E-10
NM_173047	***Cbr3***	carbonyl reductase 3	3.50	2.87E-08
NM_001033228	***Itga1***	integrin alpha 1	3.48	5.36E-11
NM_024223	***Crip2***	cysteine rich protein 2	3.45	2.08E-10
NM_009099	***Trim30a***	tripartite motif-containing 30A	3.43	4.05E-11
NM_134116	***Gpsm3***	G-protein signalling modulator 3 (AGS3-like, C. elegans)	3.43	1.25E-10
NM_023320	***Plekho1***	pleckstrin homology domain containing, family O member 1	3.37	2.49E-11
NM_019471	***Mmp10***	matrix metallopeptidase 10	3.36	1.20E-10
NM_175291	***Dock10***	dedicator of cytokinesis 10	3.36	3.03E-10
NM_213659	***Stat3***	signal transducer and activator of transcription 3	3.34	1.68E-11
NM_011578	***Tgfbr3***	transforming growth factor, beta receptor III	3.32	3.19E-10
NM_023485	***Sync***	syncoilin	3.31	2.78E-10
NR_033483	***U90926***	cDNA sequence U90926	3.30	5.15E-08
NM_001161665	***Kif26b***	kinesin family member 26B	3.29	2.56E-08
NM_023608	***Gdpd2***	glycerophosphodiester phosphodiesterase domain containing 2	3.29	7.17E-09
AK133873	***5730471H19Rik***	RIKEN cDNA 5730471H19 gene	3.29	2.37E-09
NM_001136076	***P4ha2***	procollagen-proline, 2-oxoglutarate 4-dioxygenase (proline 4-hydroxylase), alpha II polypeptid	3.28	1.75E-10
NM_007621	***Cbr2***	carbonyl reductase 2	3.28	3.50E-12
NM_026316	***Aldh3b1***	aldehyde dehydrogenase 3 family, member B1	3.28	1.06E-09
NM_027450	***Glipr2***	GLI pathogenesis-related 2	3.27	6.25E-10
NM_013778	***Akr1c13***	aldo-keto reductase family 1, member C13	3.27	5.06E-08
NM_009780	***C4b***	complement component 4B (Childo blood group)	3.27	9.86E-10
NM_007669	***Cdkn1a***	cyclin-dependent kinase inhibitor 1A (P21)	3.26	2.92E-11
NM_177782	***Prex1***	phosphatidylinositol-3,4,5-trisphosphate-dependent Rac exchange factor 1	3.26	9.12E-10
NM_172964	***Arhgap28***	Rho GTPase activating protein 28	3.25	2.13E-09
NM_011058	***Pdgfra***	platelet derived growth factor receptor, alpha polypeptide	3.25	6.20E-10
NM_173864	***Gm5077***	predicted gene 5077	3.22	3.48E-09
NM_008362	***Il1r1***	interleukin 1 receptor, type I	3.22	1.96E-10
NM_008987	***Ptx3***	pentraxin related gene	3.22	8.76E-09
NM_013642	***Dusp1***	dual specificity phosphatase 1	3.22	1.79E-10
NM_144923	***Blvrb***	biliverdin reductase B (flavin reductase (NADPH))	3.21	2.99E-09
NM_009177	***St3gal1***	ST3 beta-galactoside alpha-2,3-sialyltransferase 1	3.21	1.91E-11
NM_007392	***Acta2***	actin, alpha 2, smooth muscle, aorta	3.21	4.90E-08
NM_019835	***B4galt5***	UDP-Gal:betaGlcNAc beta 1,4-galactosyltransferase, polypeptide 5	3.20	3.98E-12
ENSMUST00000099550	***Gm10786***	predicted gene 10786	3.18	5.82E-06
NM_175358	***Zdhhc15***	zinc finger, DHHC domain containing 15	3.18	1.50E-10
NM_008318	***Ibsp***	integrin binding sialoprotein	3.17	1.03E-08
NM_018760	***Slc4a4***	solute carrier family 4 (anion exchanger), member 4	3.17	6.84E-10
NM_181344	***C1rl***	complement component 1, r subcomponent-like	3.16	8.34E-10
NM_008982	***Ptprj***	protein tyrosine phosphatase, receptor type, J	3.15	1.87E-11
NM_023732	***Abcb6***	ATP-binding cassette, sub-family B (MDR/TAP), member 6	3.15	5.01E-11
NM_029983	***Sla2***	Src-like-adaptor 2	3.12	4.78E-09
NM_008279	***Map4k1***	mitogen-activated protein kinase kinase kinase kinase 1	3.11	7.95E-10
NM_001164612	***Atp13a4***	ATPase type 13A4	3.10	3.15E-09
BC137870	***D630023F18Rik***	RIKEN cDNA D630023F18 gene	3.10	1.14E-09
NM_011414	***Slpi***	secretory leukocyte peptidase inhibitor	3.09	1.00E-12
NM_009393	***Tnnc1***	troponin C, cardiac/slow skeletal	3.08	6.75E-08
NM_010357	***Gsta4***	glutathione S-transferase, alpha 4	3.08	7.76E-11
NM_177157	***Gchfr***	GTP cyclohydrolase I feedback regulator	3.07	1.80E-09
NM_001113356	***C1rb***	complement component 1, r subcomponent B	3.06	3.26E-08
NM_001042779	***Sema3b***	sema domain, immunoglobulin domain (Ig), short basic domain, secreted, (semaphorin) 3B	3.05	2.58E-11
NM_033601	***Bcl3***	B-cell leukemia/lymphoma 3	3.05	1.27E-11
NM_001170851	***Klra2***	killer cell lectin-like receptor, subfamily A, member 2	3.05	4.51E-06
BC040401	***4933426M11Rik***	RIKEN cDNA 4933426M11 gene	3.05	1.99E-11

NM_009369	***Tgfbi***	transforming growth factor, beta induced	3.05	2.74E-10
NM_013864	***Ndrg2***	N-myc downstream regulated gene 2	3.04	2.64E-09
NM_053273	***Ttyh2***	tweety homolog 2 (Drosophila)	3.03	3.73E-10
NM_146136	***Slc16a4***	solute carrier family 16 (monocarboxylic acid transporters), member 4	3.02	3.46E-09
NM_178699	***B930041F14Rik***	RIKEN cDNA B930041F14 gene	3.02	3.30E-08
NM_010729	***Loxl1***	lysyl oxidase-like 1	3.01	5.52E-09
NM_010495	***Id1***	inhibitor of DNA binding 1	2.99	4.35E-11
NM_011633	***Traf5***	TNF receptor-associated factor 5	2.98	1.33E-10
NM_016973	***St6galnac6***	ST6 (alpha-N-acetyl-neuraminyl-2,3-beta-galactosyl-1,3)-N-acetylgalactosaminide alpha-2,6-s	2.97	6.76E-10
NM_009124	***Atxn1***	ataxin 1	2.97	1.68E-09
NM_013623	***Orm3***	orosomucoid 3	2.95	1.90E-08
NM_008496	***Lgals7***	lectin, galactose binding, soluble 7	2.95	6.83E-08
NM_009808	***Casp12***	caspase 12	2.95	1.21E-09
NM_022018	***Fam129a***	family with sequence similarity 129, member A	2.94	3.34E-07
NM_153507	***Cpne2***	copine II	2.92	2.61E-11
NM_153119	***Plekho2***	pleckstrin homology domain containing, family O member 2	2.91	3.55E-09
NM_001033238	***Cblb***	Casitas B-lineage lymphoma b	2.91	4.10E-11
NM_130861	***Slco1a5***	solute carrier organic anion transporter family, member 1a5	2.90	5.68E-07
NM_007780	***Csf2rb***	colony stimulating factor 2 receptor, beta, low-affinity (granulocyte-macrophage)	2.88	3.28E-06
NM_172648	***Ifi205***	interferon activated gene 205	2.88	6.64E-05
NM_022415	***Ptges***	prostaglandin E synthase	2.87	7.77E-11
NM_178406	***Gpr153***	G protein-coupled receptor 153	2.87	1.70E-08
NM_172729	***Nod1***	nucleotide-binding oligomerization domain containing 1	2.87	6.43E-09
NM_021460	***Lipa***	lysosomal acid lipase A	2.86	1.13E-09
NM_026672	***Gstm7***	glutathione S-transferase, mu 7	2.84	6.84E-08
NM_030127	***Htra3***	HtrA serine peptidase 3	2.83	2.76E-08
NM_008183	***Gstm2***	glutathione S-transferase, mu 2	2.83	1.55E-09
NM_027307	***Golm1***	golgi membrane protein 1	2.83	1.89E-08
NM_178114	***Amigo2***	adhesion molecule with Ig like domain 2	2.82	3.11E-11
NM_010493	***Icam1***	intercellular adhesion molecule 1	2.81	2.45E-07
NM_015753	***Zeb2***	zinc finger E-box binding homeobox 2	2.81	1.25E-09
NM_207237	***Man1c1***	mannosidase, alpha, class 1C, member 1	2.80	4.02E-11
NM_011157	***Srgn***	serglycin	2.80	3.51E-06
NM_001001326	***St5***	suppression of tumorigenicity 5	2.79	3.06E-10
NM_030725	***Syt13***	synaptotagmin XIII	2.78	3.73E-10
NM_027340	***Lipn***	lipase, family member N	2.77	1.07E-06
NM_001164036	***Ly6e***	lymphocyte antigen 6 complex, locus E	2.77	1.76E-11
NM_153510	***Pilra***	paired immunoglobin-like type 2 receptor alpha	2.76	1.46E-06
NM_007403	***Adam8***	a disintegrin and metallopeptidase domain 8	2.75	1.25E-11
NM_026837	***Tmem53***	transmembrane protein 53	2.74	1.96E-07
NM_001081160	***Mdga1***	MAM domain containing glycosylphosphatidylinositol anchor 1	2.73	2.21E-09
NM_010279	***Gfra1***	glial cell line derived neurotrophic factor family receptor alpha 1	2.73	4.20E-08
NM_029688	***Srxn1***	sulfiredoxin 1 homolog (S. cerevisiae)	2.72	7.64E-11
NM_001122660	***Gm10639***	predicted gene 10639	2.72	3.00E-08
NM_011581	***Thbs2***	thrombospondin 2	2.72	1.24E-07
NM_145475	***Cerk***	ceramide kinase	2.71	2.72E-09
BC049685	***2610528A11Rik***	RIKEN cDNA 2610528A11 gene	2.70	2.63E-09
NM_021273	***Ckb***	creatine kinase, brain	2.70	5.29E-09
NM_175502	***Tmem74***	transmembrane protein 74	2.70	2.32E-07
NM_030728	***9930013L23Rik***	RIKEN cDNA 9930013L23 gene	2.70	2.07E-08
NM_010308	***Gnao1***	guanine nucleotide binding protein, alpha O	2.69	5.11E-09
NM_011150	***Lgals3bp***	lectin, galactoside-binding, soluble, 3 binding protein	2.69	3.73E-10
NM_139307	***Vasn***	vasorin	2.68	9.20E-09
NM_172409	***Fmnl2***	formin-like 2	2.67	7.85E-11
NM_007642	***Cd28***	CD28 antigen	2.67	2.09E-07
NM_020259	***Hhip***	Hedgehog-interacting protein	2.66	1.51E-11
NM_017466	***Ccrl2***	chemokine (C-C motif) receptor-like 2	2.66	1.04E-08
NM_175256	***Heg1***	HEG homolog 1 (zebrafish)	2.66	5.11E-10
NM_010872	***Naip2***	NLR family, apoptosis inhibitory protein 2	2.65	6.93E-06
NM_008872	***Plat***	plasminogen activator, tissue	2.65	1.06E-07
NM_133667	***Pdk2***	pyruvate dehydrogenase kinase, isoenzyme 2	2.65	4.57E-09
NM_009197	***Slc16a2***	solute carrier family 16 (monocarboxylic acid transporters), member 2	2.65	7.17E-10
NM_175105	***Aqp11***	aquaporin 11	2.64	5.63E-06
NM_172463	***Sned1***	sushi, nidogen and EGF-like domains 1	2.63	1.43E-09
NM_013492	***Clu***	clusterin	2.63	4.83E-11
NM_009259	***Spn***	sialophorin	2.63	7.33E-09
NM_011610	***Tnfrsf1b***	tumor necrosis factor receptor superfamily, member 1b	2.63	5.86E-11
NM_013495	***Cpt1a***	carnitine palmitoyltransferase 1a, liver	2.62	1.97E-10
NM_027426	***2610109H07Rik***	RIKEN cDNA 2610109H07 gene	2.61	5.62E-08
NM_025972	***Naaa***	N-acylethanolamine acid amidase	2.61	1.78E-09
NM_153417	***Trpm6***	transient receptor potential cation channel, subfamily M, member 6	2.60	2.25E-08
NM_001033141	***Ecscr***	endothelial cell-specific chemotaxis regulator	2.59	2.19E-09
ENSMUST00000098492	***9330175E14Rik***	RIKEN cDNA 9330175E14 gene	2.58	1.10E-05
NM_145158	***Emilin2***	elastin microfibril interfacer 2	2.57	1.23E-06

NM_010871	***Naip6***	NLR family, apoptosis inhibitory protein 6	2.56	5.24E-07
NM_019959	***C1qtnf1***	C1q and tumor necrosis factor related protein 1	2.56	1.21E-09
NM_207244	***Cd200r4***	CD200 receptor 4	2.55	8.04E-06
NM_009349	***Inmt***	indolethylamine N-methyltransferase	2.55	1.23E-07
NM_008182	***Gsta2***	glutathione S-transferase, alpha 2 (Yc2)	2.54	3.85E-09
NM_028132	***Pgm2***	phosphoglucomutase 2	2.54	1.06E-09
NM_010433	***Hipk2***	homeodomain interacting protein kinase 2	2.53	5.44E-10
NM_001177753	***Pfkfb3***	6-phosphofructo-2-kinase/fructose-2,6-biphosphatase 3	2.53	3.24E-09
NM_031176	***Tnxb***	tenascin XB	2.53	6.95E-06
NM_011427	***Snai1***	snail homolog 1 (Drosophila)	2.53	2.16E-10
NM_021472	***Rnase4***	ribonuclease, RNase A family 4	2.52	4.04E-08
NM_144935	***BC018242***	cDNA sequence BC018242	2.52	3.14E-07
NM_001168541	***Tsku***	tsukushin	2.51	1.31E-10
NM_018811	***Abhd2***	abhydrolase domain containing 2	2.51	2.81E-11
NM_175414	***Tspan9***	tetraspanin 9	2.50	8.68E-10
NM_011075	***Abcb1b***	ATP-binding cassette, sub-family B (MDR/TAP), member 1B	2.50	3.83E-08
NM_023061	***Mcam***	melanoma cell adhesion molecule	2.50	1.77E-09
NM_013692	***Klf10***	Kruppel-like factor 10	2.50	7.94E-10
NM_030166	***Galntl2***	UDP-N-acetyl-alpha-D-galactosamine:polypeptide N-acetylgalactosaminyltransferase-like 2	2.49	1.34E-06
NM_178745	***Tmem229b***	transmembrane protein 229B	2.48	2.79E-09
NM_001113569	***Stxbp1***	syntaxin binding protein 1	2.48	3.91E-10
NM_178600	***Vkorc1***	vitamin K epoxide reductase complex, subunit 1	2.47	2.32E-07
NM_009983	***Ctsd***	cathepsin D	2.47	1.31E-10
NM_010496	***Id2***	inhibitor of DNA binding 2	2.47	1.02E-09
NM_001033209	***Xylb***	xylulokinase homolog (H. influenzae)	2.47	8.25E-09
NM_001134457	***Fam55c***	family with sequence similarity 55, member C	2.46	2.28E-07
NM_172845	***Adamts4***	a disintegrin-like and metallopeptidase (reprolysin type) with thrombospondin type 1 motif, 4	2.46	7.28E-07
NM_001111060	***Cd59a***	CD59a antigen	2.46	1.45E-07
NM_009150	***Selenbp1***	selenium binding protein 1	2.46	2.10E-05
NM_011175	***Lgmn***	legumain	2.45	5.05E-10
NM_013464	***Ahr***	aryl-hydrocarbon receptor	2.45	1.33E-10
NM_001045530	***Ccnjl***	cyclin J-like	2.44	8.07E-08
NM_001039509	***Pnkd***	paroxysmal nonkinesiogenic dyskinesia	2.43	5.02E-09
NM_153505	***Nckap1l***	NCK associated protein 1 like	2.43	2.51E-07
NM_031168	***Il6***	interleukin 6	2.43	1.14E-05
NM_026772	***Cdc42ep2***	CDC42 effector protein (Rho GTPase binding) 2	2.43	4.90E-08
NM_008885	***Pmp22***	peripheral myelin protein 22	2.42	9.80E-09
NM_013531	***Gnb4***	guanine nucleotide binding protein (G protein), beta 4	2.42	1.57E-08
NM_134437	***Il17rd***	interleukin 17 receptor D	2.42	2.32E-07
NM_001004367	***Cxxc4***	CXXC finger 4	2.42	4.70E-07
NM_013650	***S100a8***	S100 calcium binding protein A8 (calgranulin A)	2.41	1.34E-07
NM_011076	***Abcb1a***	ATP-binding cassette, sub-family B (MDR/TAP), member 1A	2.40	4.26E-07
NM_019948	***Clec4e***	C-type lectin domain family 4, member e	2.40	3.97E-06
NM_007408	***Plin2***	perilipin 2	2.40	3.96E-10
NM_010065	***Dnm1***	dynamin 1	2.38	1.07E-08
NM_172768	***Gramd1b***	GRAM domain containing 1B	2.38	6.67E-07
NM_016667	***Sntb1***	syntrophin, basic 1	2.38	3.19E-08
NM_019472	***Myo10***	myosin X	2.37	2.59E-09
NM_026470	***Spata6***	spermatogenesis associated 6	2.37	3.27E-08
NM_001033336	***Abcc4***	ATP-binding cassette, sub-family C (CFTR/MRP), member 4	2.37	2.19E-08
NM_026644	***Agpat4***	1-acylglycerol-3-phosphate O-acyltransferase 4 (lysophosphatidic acid acyltransferase, delta)	2.36	2.89E-07
NM_011526	***Tagln***	transgelin	2.36	4.10E-07
NM_023672	***Ssbp3***	single-stranded DNA binding protein 3	2.35	1.18E-10
NM_010062	***Dnase2a***	deoxyribonuclease II alpha	2.35	1.89E-09
NM_198625	***Mtss1l***	metastasis suppressor 1-like	2.35	2.59E-09
NM_007421	***Adssl1***	adenylosuccinate synthetase like 1	2.35	2.83E-06
NM_008620	***Gbp4***	guanylate binding protein 4	2.34	2.01E-07
NM_013935	***Ptpla***	protein tyrosine phosphatase-like (proline instead of catalytic arginine), member a	2.34	7.13E-07
NM_016695	***Mpp2***	membrane protein, palmitoylated 2 (MAGUK p55 subfamily member 2)	2.33	5.39E-07
NM_015744	***Enpp2***	ectonucleotide pyrophosphatase/phosphodiesterase 2	2.33	1.47E-08
NM_028133	***Egln3***	EGL nine homolog 3 (C. elegans)	2.33	8.35E-11
NM_011815	***Fyb***	FYN binding protein	2.33	8.13E-07
NM_009154	***Sema5a***	sema domain, seven thrombospondin repeats (type 1 and type 1-like), transmembrane domai	2.33	1.17E-07
NM_148926	***Zfand3***	zinc finger, AN1-type domain 3	2.33	8.72E-11
NM_201644	***Ugt1a9***	UDP glucuronosyltransferase 1 family, polypeptide A9	2.33	5.07E-09
NM_021897	***Trp53inp1***	transformation related protein 53 inducible nuclear protein 1	2.33	7.53E-10
NM_016782	***Cntnap1***	contactin associated protein-like 1	2.32	2.92E-07
NM_175445	***Rassf2***	Ras association (RalGDS/AF-6) domain family member 2	2.32	1.43E-06
NM_010513	***Igf1r***	insulin-like growth factor I receptor	2.31	4.23E-09
NM_011327	***Scp2***	sterol carrier protein 2, liver	2.31	2.53E-08
NM_001013411	***Nkain2***	Na+/K+ transporting ATPase interacting 2	2.31	8.81E-08
NM_026938	***Tmem160***	transmembrane protein 160	2.30	5.07E-10
NM_023168	***Grina***	glutamate receptor, ionotropic, N-methyl D-aspartate-associated protein 1 (glutamate binding)	2.30	2.31E-10
NM_007885	***Slc26a2***	solute carrier family 26 (sulfate transporter), member 2	2.30	8.43E-08

NM_001037987	***Edil3***	EGF-like repeats and discoidin I-like domains 3	2.30	8.44E-06
NM_008594	***Mfge8***	milk fat globule-EGF factor 8 protein	2.29	1.34E-10
NM_011125	***Pltp***	phospholipid transfer protein	2.29	9.22E-09
NM_019802	***Ggcx***	gamma-glutamyl carboxylase	2.29	1.13E-07
NM_153577	***AI428936***	expressed sequence AI428936	2.29	3.44E-09
NM_007609	***Casp4***	caspase 4, apoptosis-related cysteine peptidase	2.28	1.59E-08
NM_013671	***Sod2***	superoxide dismutase 2, mitochondrial	2.28	3.89E-10
NM_009242	***Sparc***	secreted acidic cysteine rich glycoprotein	2.28	1.55E-10
NM_001003911	***Adamts7***	a disintegrin-like and metallopeptidase (reprolysin type) with thrombospondin type 1 motif, 7	2.28	1.70E-09
NM_001033435	***Gm885***	predicted gene 885	2.28	1.28E-06
NM_009676	***Aox1***	aldehyde oxidase 1	2.28	1.07E-09
NM_001081116	***Arhgef17***	Rho guanine nucleotide exchange factor (GEF) 17	2.27	4.78E-09
NM_009994	***Cyp1b1***	cytochrome P450, family 1, subfamily b, polypeptide 1	2.27	8.72E-07
NM_027289	***Nt5dc2***	5'-nucleotidase domain containing 2	2.27	8.06E-10
NM_001081185	***Flnc***	filamin C, gamma	2.27	6.37E-09
NM_028331	***C1qtnf6***	C1q and tumor necrosis factor related protein 6	2.27	1.74E-07
NM_019783	***Lepre1***	leprecan 1	2.26	3.34E-08
NM_207648	***H2-Q6***	histocompatibility 2, Q region locus 6	2.25	3.19E-09
NM_178589	***Tnfrsf21***	tumor necrosis factor receptor superfamily, member 21	2.25	9.86E-10
NM_027249	***Tlcd2***	TLC domain containing 2	2.24	2.72E-08
NM_080448	***Srgap3***	SLIT-ROBO Rho GTPase activating protein 3	2.24	7.42E-09
NM_013770	***Slc25a10***	solute carrier family 25 (mitochondrial carrier, dicarboxylate transporter), member 10	2.24	7.53E-10
NM_145586	***Tmem159***	transmembrane protein 159	2.24	1.63E-07
NM_001110204	***Acvr1***	activin A receptor, type 1	2.24	1.81E-10
NM_020486	***Bcam***	basal cell adhesion molecule	2.24	7.72E-07
NM_029472	***Gstt4***	glutathione S-transferase, theta 4	2.23	1.80E-06
NM_027135	***Sec24d***	Sec24 related gene family, member D (S. cerevisiae)	2.23	1.42E-09
NM_024477	***Ttc28***	tetratricopeptide repeat domain 28	2.23	1.42E-07
NM_180956	***Ndrg3***	N-myc downstream regulated gene 3	2.23	2.43E-09
NM_019414	***Selenbp2***	selenium binding protein 2	2.23	1.28E-07
NM_019550	***Ptbp2***	polypyrimidine tract binding protein 2	2.22	1.77E-09
NM_001160378	***Fam46a***	family with sequence similarity 46, member A	2.22	2.17E-06
NM_173006	***Pon3***	paraoxonase 3	2.22	4.10E-10
NM_011338	***Ccl9***	chemokine (C-C motif) ligand 9	2.22	9.41E-08
NM_009883	***Cebpb***	CCAAT/enhancer binding protein (C/EBP), beta	2.22	6.10E-09
NM_133237	***Apcdd1***	adenomatosis polyposis coli down-regulated 1	2.22	2.26E-07
NM_153679	***Cpt1c***	carnitine palmitoyltransferase 1c	2.22	1.35E-07
NM_001033393	***Tmem104***	transmembrane protein 104	2.22	1.28E-07
NM_080638	***Mvp***	major vault protein	2.21	3.59E-08
NM_016769	***Smad3***	MAD homolog 3 (Drosophila)	2.21	9.87E-10
NM_019873	***Fkbpl***	FK506 binding protein-like	2.21	1.80E-07
NM_080451	***Synpo2***	synaptopodin 2	2.21	3.53E-07
NM_009034	***Rbp2***	retinol binding protein 2, cellular	2.21	3.54E-07
NM_009906	***Tpp1***	tripeptidyl peptidase I	2.21	6.41E-10
NM_015786	***Hist1h1c***	histone cluster 1, H1c	2.20	8.72E-08
NM_053113	***Ear11***	eosinophil-associated, ribonuclease A family, member 11	2.20	7.13E-05
NM_175259	***Shisa4***	shisa homolog 4 (Xenopus laevis)	2.20	2.73E-09
NM_010726	***Phyh***	phytanoyl-CoA hydroxylase	2.20	2.17E-10
NM_001081300	***Tshz1***	teashirt zinc finger family member 1	2.20	2.16E-07
NM_178665	***Lpp***	LIM domain containing preferred translocation partner in lipoma	2.19	8.53E-09
NM_019840	***Pde4b***	phosphodiesterase 4B, cAMP specific	2.19	4.55E-08
NM_013515	***Stom***	stomatin	2.19	1.17E-10
NM_011609	***Tnfrsf1a***	tumor necrosis factor receptor superfamily, member 1a	2.19	5.53E-09
NM_138665	***Sardh***	sarcosine dehydrogenase	2.18	9.07E-08
NM_172475	***Frmd4a***	FERM domain containing 4A	2.17	1.95E-08
NM_011920	***Abcg2***	ATP-binding cassette, sub-family G (WHITE), member 2	2.17	4.75E-07
NM_010851	***Myd88***	myeloid differentiation primary response gene 88	2.17	6.20E-10
NM_001033293	***Uap1l1***	UDP-N-acteylglucosamine pyrophosphorylase 1-like 1	2.17	3.80E-08
NM_177353	***Slc9a7***	solute carrier family 9 (sodium/hydrogen exchanger), member 7	2.17	1.30E-07
NM_001077499	***Scn8a***	sodium channel, voltage-gated, type VIII, alpha	2.17	2.04E-07
NM_033314	***Slco2a1***	solute carrier organic anion transporter family, member 2a1	2.17	1.30E-10
NM_023247	***Ndufaf3***	NADH dehydrogenase (ubiquinone) 1 alpha subcomplex, assembly factor 3	2.17	3.79E-08
NM_001143689	***H2-gs10***	MHC class I like protein GS10	2.17	2.74E-10
NM_027995	***Paqr7***	progestin and adipoQ receptor family member VII	2.17	1.52E-08
NM_009642	***Agtrap***	angiotensin II, type I receptor-associated protein	2.17	5.63E-09
NM_029282	***Tti1***	Tel2 interacting protein 1 homolog (S. pombe)	2.16	1.06E-09
NM_173440	***Nrip1***	nuclear receptor interacting protein 1	2.16	3.89E-10
NM_130869	***Nobox***	NOBOX oogenesis homeobox	2.16	7.74E-06
NM_018769	***Dfna5***	deafness, autosomal dominant 5 (human)	2.15	1.60E-07
NM_008175	***Grn***	granulin	2.15	2.25E-10
NM_011060	***Padi3***	peptidyl arginine deiminase, type III	2.15	3.36E-08
NM_008368	***Il2rb***	interleukin 2 receptor, beta chain	2.15	2.03E-07
NM_008094	***Gba***	glucosidase, beta, acid	2.14	7.64E-10
NM_177260	***Tmem154***	transmembrane protein 154	2.14	4.02E-06

NM_026125	***Fam132a***	family with sequence similarity 132, member A	2.14	5.44E-10
NM_026037	***Mboat2***	membrane bound O-acyltransferase domain containing 2	2.14	6.33E-05
NM_013587	***Lrpap1***	low density lipoprotein receptor-related protein associated protein 1	2.14	2.68E-09
NM_010724	***Psmb8***	proteasome (prosome, macropain) subunit, beta type 8 (large multifunctional peptidase 7)	2.14	3.20E-08
NM_011056	***Pde4d***	phosphodiesterase 4D, cAMP specific	2.14	6.48E-07
NM_007909	***Efna2***	ephrin A2	2.14	3.46E-07
NM_008549	***Man2a1***	mannosidase 2, alpha 1	2.14	1.47E-08
NM_025912	***2010011I20Rik***	RIKEN cDNA 2010011I20 gene	2.13	6.17E-09
NM_012006	***Acot1***	acyl-CoA thioesterase 1	2.13	6.95E-06
NM_133871	***Ifi44***	interferon-induced protein 44	2.13	5.68E-07
NM_001081052	***Nhs***	Nance-Horan syndrome (human)	2.13	2.52E-08
NM_001141921	***Lrfn1***	leucine rich repeat and fibronectin type III domain containing 1	2.13	2.00E-06
AF536196	***D630002G06Rik***	RIKEN cDNA D630002G06 gene	2.12	1.67E-05
NM_172903	***Man2a2***	mannosidase 2, alpha 2	2.12	5.53E-09
NM_016981	***Slc9a1***	solute carrier family 9 (sodium/hydrogen exchanger), member 1	2.12	3.30E-09
NM_027238	***Ttc39b***	tetratricopeptide repeat domain 39B	2.12	9.81E-09
NM_013641	***Ptger1***	prostaglandin E receptor 1 (subtype EP1)	2.12	7.55E-07
NM_177025	***Cobll1***	Cobl-like 1	2.12	3.03E-10
NM_023124	***H2-Q8***	histocompatibility 2, Q region locus 8	2.11	2.01E-07
NM_026991	***Sat2***	spermidine/spermine N1-acetyl transferase 2	2.11	6.94E-06
NM_010728	***Lox***	lysyl oxidase	2.11	3.46E-07
NM_001172055	***Bdh2***	3-hydroxybutyrate dehydrogenase, type 2	2.11	7.32E-07
NM_175475	***Cyp26b1***	cytochrome P450, family 26, subfamily b, polypeptide 1	2.10	3.45E-06
NM_025877	***Slc25a23***	solute carrier family 25 (mitochondrial carrier; phosphate carrier), member 23	2.10	9.01E-06
NM_144899	***Adamtsl4***	ADAMTS-like 4	2.10	1.48E-07
NM_010795	***Mgat3***	mannoside acetylglucosaminyltransferase 3	2.10	1.38E-06
NM_023154	***Ethe1***	ethylmalonic encephalopathy 1	2.10	4.55E-09
NM_001113545	***Lima1***	LIM domain and actin binding 1	2.10	9.60E-08
NM_032006	***Mmp1a***	matrix metallopeptidase 1a (interstitial collagenase)	2.10	1.47E-05
NM_018827	***Crlf1***	cytokine receptor-like factor 1	2.10	3.39E-07
NM_007387	***Acp2***	acid phosphatase 2, lysosomal	2.09	1.31E-08
NM_009712	***Arsb***	arylsulfatase B	2.09	1.67E-08
NM_175324	***Acad11***	acyl-Coenzyme A dehydrogenase family, member 11	2.09	1.00E-06
NM_009117	***Saa1***	serum amyloid A 1	2.09	1.53E-05
NM_177364	***Sh3pxd2b***	SH3 and PX domains 2B	2.09	2.83E-09
NM_176912	***Gpr77***	G protein-coupled receptor 77	2.09	1.47E-05
NM_010582	***Itih2***	inter-alpha trypsin inhibitor, heavy chain 2	2.08	7.57E-06
NM_001039156	***Triobp***	TRIO and F-actin binding protein	2.08	7.47E-10
NM_177725	***Lrrc8a***	leucine rich repeat containing 8A	2.08	3.07E-08
NM_008131	***Glul***	glutamate-ammonia ligase (glutamine synthetase)	2.08	3.71E-07
NM_178607	***Rnf24***	ring finger protein 24	2.08	3.14E-09
NM_009981	***Pcyt1a***	phosphate cytidylyltransferase 1, choline, alpha isoform	2.07	1.26E-06
NM_001033167	***Slc22a23***	solute carrier family 22, member 23	2.07	8.29E-09
NM_008561	***Mc3r***	melanocortin 3 receptor	2.07	3.04E-08
NM_010135	***Enah***	enabled homolog (Drosophila)	2.07	3.81E-09
NM_001163664	***Tnnt3***	troponin T3, skeletal, fast	2.07	5.15E-08
NM_199307	***Ece1***	endothelin converting enzyme 1	2.07	8.29E-09
NM_198294	***Tanc1***	tetratricopeptide repeat, ankyrin repeat and coiled-coil containing 1	2.06	1.43E-09
NM_011270	***Rhd***	Rh blood group, D antigen	2.06	2.98E-06
NM_021606	***Nek6***	NIMA (never in mitosis gene a)-related expressed kinase 6	2.06	3.04E-09
NM_153122	***Oplah***	5-oxoprolinase (ATP-hydrolysing)	2.06	6.43E-08
NM_001111058	***Cd33***	CD33 antigen	2.06	8.30E-08
ENSMUST00000162022	***Glis3***	GLIS family zinc finger 3	2.05	8.98E-08
NM_010234	***Fos***	FBJ osteosarcoma oncogene	2.05	1.17E-08
NM_026178	***Mmd***	monocyte to macrophage differentiation-associated	2.05	2.86E-10
NM_021394	***Zbp1***	Z-DNA binding protein 1	2.05	5.32E-09
NM_001005341	***Ypel2***	yippee-like 2 (Drosophila)	2.05	2.00E-05
NM_026153	***Ankrd33b***	ankyrin repeat domain 33B	2.05	2.02E-06
NM_001177648	***Fnbp1***	formin binding protein 1	2.05	3.51E-08
NM_028766	***Tmem43***	transmembrane protein 43	2.04	7.39E-09
NM_173388	***Slc43a2***	solute carrier family 43, member 2	2.04	4.55E-08
NM_010417	***Heph***	hephaestin	2.04	5.85E-07
NM_172371	***Slc16a13***	solute carrier family 16 (monocarboxylic acid transporters), member 13	2.04	1.92E-08
NM_023598	***Arid5b***	AT rich interactive domain 5B (MRF1-like)	2.03	5.33E-08
NM_019743	***Rybp***	RING1 and YY1 binding protein	2.03	1.37E-06
NM_174995	***Mgst2***	microsomal glutathione S-transferase 2	2.03	4.09E-07
NM_026353	***Slc48a1***	solute carrier family 48 (heme transporter), member 1	2.03	6.50E-10
NM_011671	***Ucp2***	uncoupling protein 2 (mitochondrial, proton carrier)	2.03	2.94E-10
NM_153807	***Acsf2***	acyl-CoA synthetase family member 2	2.03	2.36E-06
NM_010497	***Idh1***	isocitrate dehydrogenase 1 (NADP+), soluble	2.03	2.92E-07
NM_028608	***Glipr1***	GLI pathogenesis-related 1 (glioma)	2.03	3.31E-07
NM_133754	***Fblim1***	filamin binding LIM protein 1	2.02	4.06E-09
NM_001161790	***Mefv***	Mediterranean fever	2.02	1.54E-05
NM_172589	***Lhfpl2***	lipoma HMGIC fusion partner-like 2	2.02	1.20E-07

NM_133200	***P2ry14***	purinergic receptor P2Y, G-protein coupled, 14	2.02	8.61E-06
NM_013509	***Eno2***	enolase 2, gamma neuronal	2.02	5.90E-07
NM_144812	***Tnrc6b***	trinucleotide repeat containing 6b	2.02	1.23E-07
NM_013571	***Ksr1***	kinase suppressor of ras 1	2.02	4.07E-08
NM_027990	***Lypd6b***	LY6/PLAUR domain containing 6B	2.01	1.20E-07
NM_016687	***Sfrp4***	secreted frizzled-related protein 4	2.01	3.93E-06
NM_001114328	***Ccpg1***	cell cycle progression 1	2.01	4.80E-07
NM_201368	***Xkr8***	X Kell blood group precursor related family member 8 homolog	2.01	1.01E-06
NM_001039485	***Fam38b***	family with sequence similarity 38, member B	2.01	4.16E-05
NM_011782	***Adamts5***	a disintegrin-like and metallopeptidase (reprolysin type) with thrombospondin type 1 motif, 5 (a	2.01	2.08E-05
NM_013799	***Ate1***	arginyltransferase 1	2.01	7.17E-09
NM_182993	***Slc17a7***	solute carrier family 17 (sodium-dependent inorganic phosphate cotransporter), member 7	2.00	4.20E-07
NM_010809	***Mmp3***	matrix metallopeptidase 3	2.00	3.15E-09
NM_031159	***Apobec1***	apolipoprotein B mRNA editing enzyme, catalytic polypeptide 1	2.00	2.00E-07
NM_001039386	***Nelf***	nasal embryonic LHRH factor	2.00	8.76E-09
NM_008804	***Pde9a***	phosphodiesterase 9A	2.00	7.96E-07
NM_018884	***Pdzrn3***	PDZ domain containing RING finger 3	2.00	5.82E-09
NM_008696	***Map4k4***	mitogen-activated protein kinase kinase kinase kinase 4	2.00	7.01E-10
NM_011599	***Tle1***	transducin-like enhancer of split 1, homolog of Drosophila E(spl)	2.00	6.94E-07
NM_013599	***Mmp9***	matrix metallopeptidase 9	2.00	1.55E-09
Downregulated				
NM_001142963	***Gm10778***	predicted gene 10778	-2.00	1.83E-07
BC055110	***4921507P07Rik***	RIKEN cDNA 4921507P07 gene	-2.00	5.18E-06
NM_021788	***Sap30***	sin3 associated polypeptide	-2.00	2.88E-08
AK144596	***G730007D18Rik***	RIKEN cDNA G730007D18 gene	-2.00	2.07E-05
NM_026565	***Apool***	apolipoprotein O-like	-2.00	6.52E-06
NR_000004	***Snord35b***	small nucleolar RNA, C/D box 35B	-2.00	7.34E-05
NR_027866	***5730408K05Rik***	RIKEN cDNA 5730408K05 gene	-2.01	6.32E-07
NM_025486	***Tmem208***	transmembrane protein 208	-2.01	4.43E-07
NM_011638	***Tfrc***	transferrin receptor	-2.01	1.80E-09
BC065392	***4930522L14Rik***	RIKEN cDNA 4930522L14 gene	-2.01	1.98E-05
NM_001093752	***Srsf11***	serine/arginine-rich splicing factor 11	-2.01	1.82E-08
NM_011576	***Tfpi***	tissue factor pathway inhibitor	-2.01	2.12E-07
NM_008132	***Glrp1***	glutamine repeat protein 1	-2.01	2.90E-09
NM_001033472	***A830080D01Rik***	RIKEN cDNA A830080D01 gene	-2.01	4.49E-07
NM_011130	***Polb***	polymerase (DNA directed), beta	-2.01	2.60E-06
NM_146215	***Ftsjd1***	FtsJ methyltransferase domain containing 1	-2.01	1.37E-07
NM_025317	***Mrpl54***	mitochondrial ribosomal protein L54	-2.01	9.78E-07
NM_028725	***Sdr42e1***	short chain dehydrogenase/reductase family 42E, member 1	-2.02	1.28E-06
NM_181325	***Slc25a15***	solute carrier family 25 (mitochondrial carrier ornithine transporter), member 15	-2.02	6.63E-07
NM_172587	***Cdc14b***	CDC14 cell division cycle 14 homolog B (S. cerevisiae)	-2.02	3.99E-08
NM_011597	***Tjp2***	tight junction protein 2	-2.02	7.94E-09
NM_001195632	***Arhgap32***	Rho GTPase activating protein 32	-2.02	1.19E-08
NR_028428	***2610005L07Rik***	cadherin 11 pseudogene	-2.02	1.20E-07
NM_008857	***Prkci***	protein kinase C, iota	-2.02	3.36E-09
NM_010498	***Ids***	iduronate 2-sulfatase	-2.02	2.18E-08
NM_177806	***Prpf39***	PRP39 pre-mRNA processing factor 39 homolog (yeast)	-2.02	2.27E-05
NM_133921	***Nfxl1***	nuclear transcription factor, X-box binding-like 1	-2.02	8.41E-08
NR_027924	***2010204K13Rik***	RIKEN cDNA 2010204K13 gene	-2.02	3.25E-08
NM_178404	***Zc3h6***	zinc finger CCCH type containing 6	-2.02	3.48E-06
NM_010439	***Hmgb1***	high mobility group box 1	-2.02	0.0005203
NM_027828	***Fam110c***	family with sequence similarity 110, member C	-2.03	3.02E-07
NM_018777	***Cldn6***	claudin 6	-2.03	4.29E-06
NR_027799	***Tbrg3***	transforming growth factor beta regulated gene 3	-2.03	5.21E-05
NM_153319	***Amot***	angiomotin	-2.03	1.50E-07
NM_008188	***Thumpd3***	THUMP domain containing 3	-2.04	1.44E-06
NR_004418	***Rnu73b***	U73B small nuclear RNA	-2.04	0.0001422
NM_026497	***Nudt12***	nudix (nucleoside diphosphate linked moiety X)-type motif 12	-2.04	4.02E-07
NM_025798	***Hint3***	histidine triad nucleotide binding protein 3	-2.04	3.11E-07
NM_001012667	***AI316807***	expressed sequence AI316807	-2.04	6.02E-09
NM_133914	***Rasa4***	RAS p21 protein activator 4	-2.04	6.09E-09
NM_026995	***Carkd***	carbohydrate kinase domain containing	-2.04	4.62E-09
NM_172407	***Cdkn2aip***	CDKN2A interacting protein	-2.04	4.55E-10
NM_144519	***Zfp639***	zinc finger protein 639	-2.04	1.89E-07
NM_009261	***Strbp***	spermatid perinuclear RNA binding protein	-2.04	3.92E-08
NM_001081316	***Dsel***	dermatan sulfate epimerase-like	-2.05	2.43E-07
NM_175439	***Mars2***	methionine-tRNA synthetase 2 (mitochondrial)	-2.05	1.26E-07
NM_008756	***Ocln***	occludin	-2.05	1.43E-06
NM_019668	***Ube2a***	ubiquitin-conjugating enzyme E2A, RAD6 homolog (S. cerevisiae)	-2.05	7.13E-07
NM_001039373	***Mtcp1***	mature T-cell proliferation 1	-2.05	4.73E-07
NM_198020	***Trmt1***	TRM1 tRNA methyltransferase 1 homolog (S. cerevisiae)	-2.05	4.52E-09
NM_001163564	***Naf1***	nuclear assembly factor 1 homolog (S. cerevisiae)	-2.05	5.25E-08
NM_011988	***Slc27a3***	solute carrier family 27 (fatty acid transporter), member 3	-2.05	3.12E-07
NM_175193	***Golim4***	golgi integral membrane protein 4	-2.05	1.44E-06

BC147845	***Uprt***	uracil phosphoribosyltransferase (FUR1) homolog (S. cerevisiae)	-2.05	5.38E-09
NM_011580	***Thbs1***	thrombospondin 1	-2.05	9.18E-10
BC076612	***3110043O21Rik***	RIKEN cDNA 3110043O21 gene	-2.05	1.83E-07
NM_153161	***BC037034***	cDNA sequence BC037034	-2.06	7.91E-07
BC150999	***Fam178a***	family with sequence similarity 178, member A	-2.06	1.42E-07
NM_080634	***Hps3***	Hermansky-Pudlak syndrome 3 homolog (human)	-2.06	6.09E-07
NM_028230	***Shmt2***	serine hydroxymethyltransferase 2 (mitochondrial)	-2.06	5.07E-09
NM_011972	***Poli***	polymerase (DNA directed), iota	-2.06	1.74E-07
NM_027978	***Coq2***	coenzyme Q2 homolog, prenyltransferase (yeast)	-2.06	1.48E-07
NM_010765	***Mapkapk5***	MAP kinase-activated protein kinase 5	-2.06	8.57E-10
NM_175350	***Tmem146***	transmembrane protein 146	-2.06	4.18E-07
NM_144861	***Rprd1a***	regulation of nuclear pre-mRNA domain containing 1A	-2.06	9.98E-08
NM_001039060	***Klhl15***	kelch-like 15 (Drosophila)	-2.07	3.10E-08
NM_011386	***Skil***	SKI-like	-2.07	4.79E-09
NM_027968	***Fbxo30***	F-box protein 30	-2.07	4.98E-09
NM_080636	***Hars2***	histidyl-tRNA synthetase 2, mitochondrial (putative)	-2.07	1.91E-09
NM_001098233	***Purg***	purine-rich element binding protein G	-2.07	6.15E-07
NM_026057	***Zfp422***	zinc finger protein 422	-2.07	5.81E-09
NM_133255	***Hook2***	hook homolog 2 (Drosophila)	-2.07	1.04E-08
NM_001142959	***Bcl2l15***	BCLl2-like 15	-2.07	2.75E-07
NM_012026	***Rgnef***	Rho-guanine nucleotide exchange factor	-2.07	9.04E-09
NM_008921	***Prim1***	DNA primase, p49 subunit	-2.07	1.54E-08
NM_178688	***Ablim1***	actin-binding LIM protein 1	-2.08	3.90E-08
NM_172405	***Fam175a***	family with sequence similarity 175, member A	-2.08	9.71E-06
NM_028011	***Tom1l1***	target of myb1-like 1 (chicken)	-2.08	2.15E-08
NM_026910	***Tnik***	TRAF2 and NCK interacting kinase	-2.08	5.44E-07
NM_028994	***Pck2***	phosphoenolpyruvate carboxykinase 2 (mitochondrial)	-2.08	6.37E-09
NM_029321	***Ttc32***	tetratricopeptide repeat domain 32	-2.08	3.37E-06
NM_001013816	***Gm5622***	predicted gene 5622	-2.08	7.30E-05
NM_009006	***Map4k2***	mitogen-activated protein kinase kinase kinase kinase 2	-2.08	5.30E-08
NM_001164099	***Add3***	adducin 3 (gamma)	-2.08	1.40E-07
NM_027853	***Mettl7b***	methyltransferase like 7B	-2.08	1.21E-06
NM_001122759	***Pde7a***	phosphodiesterase 7A	-2.08	3.24E-08
NM_175353	***Exoc6***	exocyst complex component 6	-2.08	1.09E-09
NM_201351	***Cybasc3***	cytochrome b, ascorbate dependent 3	-2.08	1.30E-05
NR_028566	***Snord118***	small nucleolar RNA, C/D box 118	-2.08	0.0203528
NM_175369	***Ccdc122***	coiled-coil domain containing 122	-2.08	7.19E-06
NM_181730	***Polr1d***	polymerase (RNA) I polypeptide D	-2.09	8.47E-09
NM_008784	***Igbp1***	immunoglobulin (CD79A) binding protein 1	-2.09	7.61E-09
NR_033443	***Sept6***	septin 6	-2.09	1.68E-09
NM_011643	***Trpc1***	transient receptor potential cation channel, subfamily C, member 1	-2.09	2.09E-05
NM_001001932	***Eea1***	early endosome antigen 1	-2.09	1.74E-06
BC150968	***Pten***	phosphatase and tensin homolog	-2.09	4.06E-05
NM_172051	***Tmcc3***	transmembrane and coiled coil domains 3	-2.09	1.61E-07
NM_019773	***Rab9***	RAB9, member RAS oncogene family	-2.10	2.51E-06
NM_133900	***Psph***	phosphoserine phosphatase	-2.10	6.31E-08
NM_010553	***Il18rap***	interleukin 18 receptor accessory protein	-2.10	2.35E-07
NM_028394	***Hspb11***	heat shock protein family B (small), member 11	-2.10	5.67E-06
NM_145568	***Krcc1***	lysine-rich coiled-coil 1	-2.10	3.59E-06
NM_182996	***Zfp692-ps***	zinc finger protein 692, pseudogene	-2.10	3.04E-09
NM_001008501	***Zfp760***	zinc finger protein 760	-2.10	2.70E-06
NM_009214	***Sms***	spermine synthase	-2.10	3.37E-09
ENSMUST00000098707	***Gm10661***	predicted gene 10661	-2.10	2.68E-08
NM_016710	***Hmgn5***	high-mobility group nucleosome binding domain 5	-2.10	1.47E-05
NM_153780	***2610044O15Rik***	RIKEN cDNA 2610044O15 gene	-2.11	0.0058398
NM_001037841	***Cklf***	chemokine-like factor	-2.11	2.00E-07
NM_134137	***Lars***	leucyl-tRNA synthetase	-2.12	3.04E-09
NM_008638	***Mthfd2***	methylenetetrahydrofolate dehydrogenase (NAD+ dependent), methenyltetrahydrofolate cyclc	-2.12	2.11E-08
NM_019961	***Pex3***	peroxisomal biogenesis factor 3	-2.12	1.93E-09
NM_139269	***Pla2g16***	phospholipase A2, group XVI	-2.12	4.98E-06
NM_172538	***Vezt***	vezatin, adherens junctions transmembrane protein	-2.12	2.36E-08
NM_001037918	***Lipt1***	lipoyltransferase 1	-2.12	1.86E-06
NM_024214	***Tomm20***	translocase of outer mitochondrial membrane 20 homolog (yeast)	-2.12	4.25E-07
NM_008253	***Hmgb3***	high mobility group box 3	-2.12	4.63E-08
NM_177429	***Ofd1***	oral-facial-digital syndrome 1 gene homolog (human)	-2.13	8.05E-06
NM_053178	***Acsbg1***	acyl-CoA synthetase bubblegum family member 1	-2.13	5.29E-09
NM_010898	***Nf2***	neurofibromatosis 2	-2.13	1.95E-07
NM_201369	***N4bp2l2***	NEDD4 binding protein 2-like 2	-2.13	4.74E-09
NM_025949	***Rps6ka6***	ribosomal protein S6 kinase polypeptide 6	-2.13	5.98E-08
NM_031251	***Ctns***	cystinosis, nephropathic	-2.13	1.21E-07
NM_146006	***Lss***	lanosterol synthase	-2.13	5.65E-07
NM_010836	***Msx3***	homeobox, msh-like 3	-2.13	1.71E-06
NM_010848	***Myb***	myeloblastosis oncogene	-2.13	5.15E-09
NM_144804	***Depdc7***	DEP domain containing 7	-2.13	1.84E-09

NM_013563	***Il2rg***	interleukin 2 receptor, gamma chain	-2.14	6.74E-07
NM_001081001	***Brca2***	breast cancer 2	-2.14	9.20E-09
ENSMUST00000072582	***Fabp5l2***	fatty acid binding protein 5-like 2	-2.14	6.52E-07
BC052497	***2010007H12Rik***	RIKEN cDNA 2010007H12 gene	-2.15	6.64E-08
NM_145221	***Appl1***	adaptor protein, phosphotyrosine interaction, PH domain and leucine zipper containing 1	-2.15	2.06E-09
NR_002898	***Snora65***	small nucleolar RNA, H/ACA box 65	-2.15	2.09E-07
NM_183275	***1110002N22Rik***	RIKEN cDNA 1110002N22 gene	-2.15	4.11E-08
NM_029730	***Mospd2***	motile sperm domain containing 2	-2.15	3.47E-08
NM_145404	***Prmt7***	protein arginine N-methyltransferase 7	-2.15	3.19E-09
NM_019990	***Stard10***	START domain containing 10	-2.15	7.39E-09
NM_001033474	***Atxn7l3b***	ataxin 7-like 3B	-2.15	2.11E-09
ENSMUST00000089906	***2310001H12Rik***	RIKEN cDNA 2310001H12 gene	-2.15	2.23E-06
NM_023292	***Pus3***	pseudouridine synthase 3	-2.15	3.55E-09
NM_028013	***Endod1***	endonuclease domain containing 1	-2.15	1.84E-09
BC013508	***9130017N09Rik***	RIKEN cDNA 9130017N09 gene	-2.16	7.52E-07
NM_173874	***Clcn3***	chloride channel 3	-2.16	1.20E-07
NM_028448	***Cenpv***	centromere protein V	-2.16	1.27E-10
NM_153163	***Cadps2***	Ca2+-dependent activator protein for secretion 2	-2.16	1.33E-05
NM_007616	***Cav1***	caveolin 1, caveolae protein	-2.17	1.99E-06
NM_016912	***Cdkl2***	cyclin-dependent kinase-like 2 (CDC2-related kinase)	-2.17	1.24E-08
NM_001099314	***Msmp***	microseminoprotein, prostate associated	-2.17	3.62E-07
NM_013738	***Plek2***	pleckstrin 2	-2.17	6.00E-07
NM_011858	***Odz4***	odd Oz/ten-m homolog 4 (Drosophila)	-2.18	2.80E-07
NM_025539	***Nudt2***	nudix (nucleoside diphosphate linked moiety X)-type motif 2	-2.18	3.97E-06
NM_025833	***Baiap2l1***	BAI1-associated protein 2-like 1	-2.18	2.45E-08
NM_023900	***Plekhj1***	pleckstrin homology domain containing, family J member 1	-2.18	1.62E-09
NM_007677	***Psg17***	pregnancy specific glycoprotein 17	-2.19	1.44E-06
NM_011206	***Ptpn18***	protein tyrosine phosphatase, non-receptor type 18	-2.19	1.39E-06
NM_009354	***Tert***	telomerase reverse transcriptase	-2.19	2.78E-07
NM_145973	***Ell3***	elongation factor RNA polymerase II-like 3	-2.20	1.66E-05
NM_053272	***Dhcr24***	24-dehydrocholesterol reductase	-2.20	1.68E-08
NR_028550	***Snord49a***	small nucleolar RNA, C/D box 49A	-2.20	2.36E-06
NM_026887	***Ap1s2***	adaptor-related protein complex 1, sigma 2 subunit	-2.20	4.30E-05
NM_017465	***Sult2b1***	sulfotransferase family, cytosolic, 2B, member 1	-2.20	1.91E-07
BC023385	***1110059E24Rik***	RIKEN cDNA 1110059E24 gene	-2.20	6.48E-07
NM_133942	***Plekha1***	pleckstrin homology domain containing, family A (phosphoinositide binding specific) member	-2.20	4.98E-06
NM_001081056	***Xpot***	exportin, tRNA (nuclear export receptor for tRNAs)	-2.20	7.57E-10
NM_001082975	***Sdr39u1***	short chain dehydrogenase/reductase family 39U, member 1	-2.20	3.05E-09
NM_175116	***Lpar6***	lysophosphatidic acid receptor 6	-2.21	9.44E-09
NM_172771	***Dmxl2***	Dmx-like 2	-2.21	1.62E-07
NM_177784	***Klhl23***	kelch-like 23 (Drosophila)	-2.21	2.20E-08
NM_027077	***1700016C15Rik***	RIKEN cDNA 1700016C15 gene	-2.21	3.78E-06
NR_027874	***0610040B10Rik***	RIKEN cDNA 0610040B10 gene	-2.21	1.17E-06
NM_010590	***Jub***	ajuba	-2.21	2.28E-07
NM_001191004	***Lsm6***	LSM6 homolog, U6 small nuclear RNA associated (S. cerevisiae)	-2.21	1.03E-07
NM_026120	***2410127L17Rik***	RIKEN cDNA 2410127L17 gene	-2.21	1.71E-07
ENSMUST00000097783	***D1Ertd448e***	DNA segment, Chr 1, ERATO Doi 448, expressed	-2.21	3.38E-08
NM_001146351	***Ephb6***	Eph receptor B6	-2.21	1.74E-08
NM_173442	***Gcnt1***	glucosaminyl (N-acetyl) transferase 1, core 2	-2.22	1.75E-08
NM_026110	***Gcfc1***	GC-rich sequence DNA-binding factor 1	-2.22	1.46E-06
NM_026572	***Gcsh***	glycine cleavage system protein H (aminomethyl carrier)	-2.22	2.36E-08
NM_010352	***Gsg1***	germ cell-specific gene 1	-2.22	1.45E-07
NM_019812	***Sirt1***	sirtuin 1 (silent mating type information regulation 2, homolog) 1 (S. cerevisiae)	-2.22	1.64E-09
NR_033336	***Snora23***	small nucleolar RNA, H/ACA box 23	-2.22	3.27E-07
NM_001164627	***Arhgap8***	Rho GTPase activating protein 8	-2.22	3.81E-09
NM_019864	***Atr***	ataxia telangiectasia and Rad3 related	-2.22	4.53E-09
NM_028623	***Cst6***	cystatin E/M	-2.22	1.97E-06
NR_028480	***Gm12238***	predicted gene 12238	-2.23	1.17E-06
NM_028785	***Dock8***	dedicator of cytokinesis 8	-2.23	6.19E-08
NM_026004	***Nt5c3***	5'-nucleotidase, cytosolic III	-2.23	4.88E-07
NM_021438	***Fibp***	fibroblast growth factor (acidic) intracellular binding protein	-2.23	2.51E-10
NM_020293	***Cldn9***	claudin 9	-2.23	1.90E-06
NM_011135	***Cnot7***	CCR4-NOT transcription complex, subunit 7	-2.23	4.63E-11
NM_173740	***Maoa***	monoamine oxidase A	-2.24	6.04E-08
NM_028572	***Vgll3***	vestigial like 3 (Drosophila)	-2.24	5.17E-06
BC048939	***A130022J15Rik***	RIKEN cDNA A130022J15 gene	-2.24	4.90E-09
NM_027460	***Slc25a33***	solute carrier family 25, member 33	-2.24	2.21E-06
NM_007712	***Clk2***	CDC-like kinase 2	-2.24	6.20E-10
NM_177721	***Ranbp6***	RAN binding protein 6	-2.24	2.42E-07
NM_001039137	***Scoc***	short coiled-coil protein	-2.25	3.32E-05
NM_010104	***Edn1***	endothelin 1	-2.25	1.33E-06
NM_153552	***Thoc1***	THO complex 1	-2.25	3.70E-06
NR_028271	***Phxr4***	per-hexamer repeat gene 4	-2.26	5.22E-05
NM_028319	***Zfp518a***	zinc finger protein 518A	-2.26	3.93E-06

NM_019429	***Prss16***	protease, serine, 16 (thymus)	-2.26	4.65E-08
NM_175160	***Zdhhc1***	zinc finger, DHHC domain containing 1	-2.26	3.60E-09
NM_175234	***6230409E13Rik***	RIKEN cDNA 6230409E13 gene	-2.26	2.57E-08
NM_011048	***Pcsk6***	proprotein convertase subtilisin/kexin type 6	-2.26	2.09E-05
NM_001146081	***Fancb***	Fanconi anemia, complementation group B	-2.26	4.51E-08
NM_001081473	***Zxdb***	zinc finger, X-linked, duplicated B	-2.26	8.28E-10
NM_172941	***Zkscan17***	zinc finger with KRAB and SCAN domains 17	-2.27	5.42E-07
NM_026591	***Mrpl24***	mitochondrial ribosomal protein L24	-2.27	1.16E-07
NM_021350	***Chml***	choroideremia-like	-2.27	7.22E-10
NM_010663	***Krt17***	keratin 17	-2.27	1.89E-07
NM_001162485	***Arrdc1***	arrestin domain containing 1	-2.27	7.34E-10
NM_028287	***Zufsp***	zinc finger with UFM1-specific peptidase domain	-2.27	1.13E-06
NM_001110271	***Abhd14a***	abhydrolase domain containing 14A	-2.27	1.51E-09
NM_175185	***Hsdl1***	hydroxysteroid dehydrogenase like 1	-2.27	8.25E-09
NM_028478	***Rassf6***	Ras association (RalGDS/AF-6) domain family member 6	-2.27	3.96E-05
NM_008822	***Pex7***	peroxisomal biogenesis factor 7	-2.27	6.18E-07
NM_145952	***Tbc1d12***	TBC1D12: TBC1 domain family, member 12	-2.27	9.87E-08
NR_015602	***F730043M19Rik***	RIKEN cDNA F730043M19 gene	-2.28	5.76E-09
NM_001037711	***Cgn***	cingulin	-2.28	6.21E-07
NR_002905	***Snora74a***	small nucleolar RNA, H/ACA box 74A	-2.28	7.14E-05
NM_025978	***Ttc14***	tetratricopeptide repeat domain 14	-2.28	1.55E-06
NM_001081411	***Sclt1***	sodium channel and clathrin linker 1	-2.28	1.42E-06
NR_004419	***Rny1***	RNA, Y1 small cytoplasmic, Ro-associated	-2.28	0.0004151
NM_013898	***Timm8a1***	translocase of inner mitochondrial membrane 8 homolog a1 (yeast)	-2.28	3.15E-07
NM_001081020	***Adamts6***	a disintegrin-like and metallopeptidase (reprolysin type) with thrombospondin type 1 motif, 6	-2.29	9.32E-08
BC064022	***2610005L07Rik***	cadherin 11 pseudogene	-2.29	4.52E-08
NM_029495	***Epsti1***	epithelial stromal interaction 1 (breast)	-2.29	3.95E-08
NM_016900	***Cav2***	caveolin 2	-2.29	1.59E-09
NM_145099	***Trpv3***	transient receptor potential cation channel, subfamily V, member 3	-2.29	6.05E-07
NM_009753	***Bicd1***	bicaudal D homolog 1 (Drosophila)	-2.30	3.43E-08
NM_025349	***Lsm7***	LSM7 homolog, U6 small nuclear RNA associated (S. cerevisiae)	-2.30	3.17E-08
NR_028275	***Snord14e***	small nucleolar RNA, C/D box 14E	-2.30	1.34E-08
NM_001001184	***Ccdc111***	coiled-coil domain containing 111	-2.31	1.40E-07
NM_011375	***St3gal5***	ST3 beta-galactoside alpha-2,3-sialyltransferase 5	-2.31	2.46E-08
NM_016707	***Bcl11a***	B-cell CLL/lymphoma 11A (zinc finger protein)	-2.31	3.00E-07
NM_027617	***Spata1***	spermatogenesis associated 1	-2.32	8.55E-07
NM_026362	***5033414D02Rik***	RIKEN cDNA 5033414D02 gene	-2.32	3.65E-08
NM_025511	***Fam36a***	family with sequence similarity 36, member A	-2.32	1.97E-07
NM_011177	***Klk6***	kallikrein related-peptidase 6	-2.32	7.91E-08
NM_197992	***Pcgf1***	polycomb group ring finger 1	-2.32	1.10E-05
NM_001195025	***Nuak2***	NUAK family, SNF1-like kinase, 2	-2.32	8.12E-08
NM_172696	***Inadl***	InaD-like (Drosophila)	-2.32	2.13E-09
NM_001024703	***Mctp2***	multiple C2 domains, transmembrane 2	-2.32	1.96E-06
NM_001110017	***Dzip3***	DAZ interacting protein 3, zinc finger	-2.33	5.14E-09
NM_133974	***Cdcp1***	CUB domain containing protein 1	-2.33	1.80E-09
NM_025950	***Cdc37l1***	cell division cycle 37 homolog (S. cerevisiae)-like 1	-2.33	5.28E-08
NM_008824	***Pfkfb1***	6-phosphofructo-2-kinase/fructose-2,6-biphosphatase 1	-2.33	1.01E-08
NM_028234	***Rbm33***	RNA binding motif protein 33	-2.34	2.28E-06
NM_027654	***Pcgf6***	polycomb group ring finger 6	-2.34	6.23E-09
NM_001081413	***Unc13b***	unc-13 homolog B (C. elegans)	-2.34	3.77E-07
NM_134065	***Epdr1***	ependymin related protein 1 (zebrafish)	-2.35	3.39E-06
NM_016866	***Stk39***	serine/threonine kinase 39, STE20/SPS1 homolog (yeast)	-2.35	1.09E-09
NM_133358	***Zfp617***	zinc finger protein 617	-2.35	1.51E-05
NR_028479	***Snora20***	small nucleolar RNA, H/ACA box 20	-2.35	9.88E-05
NR_028564	***Snord95***	small nucleolar RNA, C/D box 95	-2.35	2.35E-08
BC028765	***0610010O12Rik***	RIKEN cDNA 0610010O12 gene	-2.35	1.61E-09
NM_172473	***Hace1***	HECT domain and ankyrin repeat containing, E3 ubiquitin protein ligase 1	-2.35	1.78E-10
ENSMUST00000068250	***Gm9958***	predicted gene 9958	-2.36	2.33E-05
NM_001033172	***Rab11fip2***	RAB11 family interacting protein 2 (class I)	-2.36	5.59E-11
NM_147155	***Tagap1***	T-cell activation GTPase activating protein 1	-2.36	7.53E-08
NM_178395	***Zdhhc2***	zinc finger, DHHC domain containing 2	-2.36	7.39E-09
NM_001024606	***Pdp2***	pyruvate dehyrogenase phosphatase catalytic subunit 2	-2.36	8.46E-07
NM_007825	***Cyp7b1***	cytochrome P450, family 7, subfamily b, polypeptide 1	-2.36	5.90E-08
NM_019745	***Pdcd10***	programmed cell death 10	-2.36	1.04E-06
NM_009951	***Igf2bp1***	insulin-like growth factor 2 mRNA binding protein 1	-2.36	7.50E-09
NR_031758	***Snora26***	small nucleolar RNA, H/ACA box 26	-2.37	4.61E-07
NM_001025307	***Stx3***	syntaxin 3	-2.37	3.88E-08
NM_145942	***Hmgcs1***	3-hydroxy-3-methylglutaryl-Coenzyme A synthase 1	-2.37	4.92E-10
NM_023792	***Pank1***	pantothenate kinase 1	-2.37	3.19E-08
NM_019699	***Fads2***	fatty acid desaturase 2	-2.37	2.94E-10
NM_029614	***Prss23***	protease, serine, 23	-2.38	1.34E-09
NM_019552	***Abcb10***	ATP-binding cassette, sub-family B (MDR/TAP), member 10	-2.38	1.47E-07
NM_001033149	***Ttc9***	tetratricopeptide repeat domain 9	-2.38	6.78E-09
NM_009051	***Rex2***	reduced expression 2	-2.38	4.20E-09

NR_028095	***Rabggtb***	RAB geranylgeranyl transferase, b subunit	-2.39	4.85E-09
NM_013755	***Gyg***	glycogenin	-2.39	1.49E-08
NM_019738	***Nupr1***	nuclear protein 1	-2.39	3.91E-09
NM_027777	***Pex1***	peroxisomal biogenesis factor 1	-2.39	2.11E-05
NM_144911	***Rpap2***	RNA polymerase II associated protein 2	-2.39	7.97E-07
NM_019679	***Fmnl1***	formin-like 1	-2.40	6.02E-10
NM_009706	***Arhgap5***	Rho GTPase activating protein 5	-2.41	3.15E-05
NM_013494	***Cpe***	carboxypeptidase E	-2.41	1.03E-08
NM_172833	***Malt1***	mucosa associated lymphoid tissue lymphoma translocation gene 1	-2.41	1.62E-08
ENSMUST00000111864	***Gm5930***	predicted gene 5930	-2.42	5.84E-05
NM_019804	***B4galt4***	UDP-Gal:betaGlcNAc beta 1,4-galactosyltransferase, polypeptide 4	-2.42	2.08E-07
NM_025410	***Lage3***	L antigen family, member 3	-2.42	6.15E-08
NM_172532	***Aldh5a1***	aldhehyde dehydrogenase family 5, subfamily A1	-2.43	2.31E-06
NM_011172	***Prodh***	proline dehydrogenase	-2.43	2.26E-07
NM_146074	***Tfb1m***	transcription factor B1, mitochondrial	-2.44	6.30E-09
NM_028604	***Trmt11***	tRNA methyltransferase 11 homolog (S. cerevisiae)	-2.44	2.97E-07
NM_001039514	***Dhps***	deoxyhypusine synthase	-2.44	1.74E-08
NM_013906	***Adamts8***	a disintegrin-like and metallopeptidase (reprolysin type) with thrombospondin type 1 motif, 8	-2.44	1.27E-08
NM_001166581	***BC005561***	cDNA sequence BC005561	-2.44	1.07E-06
NM_145376	***Lpcat1***	lysophosphatidylcholine acyltransferase 1	-2.44	2.27E-09
NM_013490	***Chka***	choline kinase alpha	-2.44	4.23E-11
NM_007948	***Ercc1***	excision repair cross-complementing rodent repair deficiency, complementation group 1	-2.45	1.31E-10
NM_027107	***2310004N24Rik***	RIKEN cDNA 2310004N24 gene	-2.45	3.10E-05
NM_009434	***Phlda2***	pleckstrin homology-like domain, family A, member 2	-2.45	2.78E-10
NM_001122594	***Phlpp2***	PH domain and leucine rich repeat protein phosphatase 2	-2.45	1.26E-08
NM_025669	***Sfrs18***	serine/arginine-rich splicing factor 18	-2.45	2.43E-08
NM_001038015	***Gnpda2***	glucosamine-6-phosphate deaminase 2	-2.45	6.46E-10
NM_178753	***Spin4***	spindlin family, member 4	-2.45	7.50E-10
NM_010415	***Hbegf***	heparin-binding EGF-like growth factor	-2.45	4.66E-10
NM_197982	***Ddx39***	DEAD (Asp-Glu-Ala-Asp) box polypeptide 39	-2.46	8.28E-10
NM_001081047	***Cnksr1***	connector enhancer of kinase suppressor of Ras 1	-2.46	9.86E-10
NM_001109747	***Cenpw***	centromere protein W	-2.46	9.76E-07
NM_001025365	***Miip***	migration and invasion inhibitory protein	-2.46	1.21E-09
NM_001024846	***Zfp62***	zinc finger protein 62	-2.46	1.82E-05
NM_001008502	***Bbs12***	Bardet-Biedl syndrome 12 (human)	-2.46	3.11E-08
NM_025969	***1700034H14Rik***	RIKEN cDNA 1700034H14 gene	-2.47	5.66E-07
NM_027294	***Cmtm8***	CKLF-like MARVEL transmembrane domain containing 8	-2.47	2.89E-06
NM_021367	***Tslp***	thymic stromal lymphopoietin	-2.47	8.95E-09
NM_001037863	***Atp11c***	ATPase, class VI, type 11C	-2.47	1.77E-08
NM_029801	***1700021C14Rik***	RIKEN cDNA 1700021C14 gene	-2.48	1.82E-07
NM_146117	***Lrrc26***	leucine rich repeat containing 26	-2.48	1.60E-07
NM_139292	***Reep6***	receptor accessory protein 6	-2.48	3.58E-07
NM_001033261	***Zfc3h1***	zinc finger, C3H1-type containing	-2.48	1.59E-10
NM_144931	***Nae1***	NEDD8 activating enzyme E1 subunit 1	-2.49	1.61E-09
NM_153123	***Atf7ip2***	activating transcription factor 7 interacting protein 2	-2.49	1.51E-05
NM_001164630	***Mum1l1***	melanoma associated antigen (mutated) 1-like 1	-2.49	5.20E-07
NM_008979	***Ptpn22***	protein tyrosine phosphatase, non-receptor type 22 (lymphoid)	-2.49	3.37E-09
NR_028273	***Snord14a***	small nucleolar RNA, C/D box 14A	-2.49	3.70E-07
NM_013470	***Anxa3***	annexin A3	-2.50	2.06E-11
NM_029019	***Stard6***	StAR-related lipid transfer (START) domain containing 6	-2.50	1.41E-06
NM_013822	***Jag1***	jagged 1	-2.50	9.18E-10
AB010352	***AB010352***	cDNA sequence AB010352	-2.51	8.30E-05
BC019498	***1500011H22Rik***	RIKEN cDNA 1500011H22 gene	-2.51	5.67E-11
NM_153596	***Tmem17***	transmembrane protein 17	-2.51	2.99E-09
NM_015828	***Gne***	glucosamine	-2.52	1.51E-09
NM_001081191	***Eml5***	echinoderm microtubule associated protein like 5	-2.52	1.80E-09
BC075731	***Rpl12***	ribosomal protein L12	-2.52	2.20E-07
NM_007739	***Col8a1***	collagen, type VIII, alpha 1	-2.52	1.62E-10
NM_025478	***Isoc1***	isochorismatase domain containing 1	-2.53	5.24E-11
NM_030110	***Efha2***	EF-hand domain family, member A2	-2.53	3.46E-05
NM_177420	***Psat1***	phosphoserine aminotransferase 1	-2.53	3.81E-11
NM_177586	***Eif5a2***	eukaryotic translation initiation factor 5A2	-2.53	9.93E-09
NM_025564	***Magohb***	mago-nashi homolog B (Drosophila)	-2.53	3.67E-05
NM_023716	***Tubb2b***	tubulin, beta 2B	-2.53	1.25E-07
NM_172992	***Phtf2***	putative homeodomain transcription factor 2	-2.53	1.58E-08
NM_153543	***Aldh1l2***	aldehyde dehydrogenase 1 family, member L2	-2.54	3.49E-08
NM_029965	***Rnf170***	ring finger protein 170	-2.54	5.85E-08
NM_198019	***Cep78***	centrosomal protein 78	-2.54	8.15E-10
NM_029094	***Pik3cb***	phosphatidylinositol 3-kinase, catalytic, beta polypeptide	-2.54	4.40E-09
NM_027510	***3830403N18Rik***	RIKEN cDNA 3830403N18 gene	-2.55	1.27E-05
NM_008018	***Sh3pxd2a***	SH3 and PX domains 2A	-2.56	5.43E-08
NM_199195	***Bckdhb***	branched chain ketoacid dehydrogenase E1, beta polypeptide	-2.57	1.21E-09
ENSMUST00000135146	***Coq2***	coenzyme Q2 homolog, prenyltransferase (yeast)	-2.57	2.83E-08
NM_019971	***Pdgfc***	platelet-derived growth factor, C polypeptide	-2.57	5.01E-10

NM_001013379	***D10627***	cDNA sequence D10627	-2.57	1.46E-08
NM_027834	***9130008F23Rik***	RIKEN cDNA 9130008F23 gene	-2.58	1.23E-07
NM_177869	***Fam185a***	family with sequence similarity 185, member A	-2.58	6.65E-10
NM_144912	***Rad9b***	RAD9 homolog B (S. cerevisiae)	-2.59	2.92E-06
NM_016960	***Ccl20***	chemokine (C-C motif) ligand 20	-2.60	6.61E-08
NM_001033329	***Arhgef9***	CDC42 guanine nucleotide exchange factor (GEF) 9	-2.60	3.66E-07
NM_001163288	***Susd1***	sushi domain containing 1	-2.61	7.39E-09
NM_146089	***Haus1***	HAUS augmin-like complex, subunit 1	-2.61	1.54E-08
NM_027261	***Taf1d***	TATA box binding protein (Tbp)-associated factor, RNA polymerase I, D	-2.61	2.27E-07
NM_146042	***Rnf144b***	ring finger protein 144B	-2.61	6.84E-09
NR_028276	***Snord14c***	small nucleolar RNA, C/D box 14C	-2.62	3.81E-09
NR_002903	***Snord61***	small nucleolar RNA, C/D box 61	-2.62	0.0009511
NM_013657	***Sema3c***	sema domain, immunoglobulin domain (Ig), short basic domain, secreted, (semaphorin) 3C	-2.62	1.19E-06
NM_001033321	***Tmem231***	transmembrane protein 231	-2.63	1.24E-08
BC012405	***2310001H12Rik***	RIKEN cDNA 2310001H12 gene	-2.63	7.59E-09
NM_029891	***Nkrf***	NF-kappaB repressing factor	-2.63	1.24E-08
NM_026902	***Mcts1***	malignant T cell amplified sequence 1	-2.64	5.22E-07
NM_172754	***Zfp868***	zinc finger protein 868	-2.64	6.77E-09
NM_207213	***Snx25***	sorting nexin 25	-2.65	8.62E-08
NR_028079	***Snora3***	small nucleolar RNA, H/ACA box 3	-2.65	1.24E-06
NM_001081081	***Gls***	glutaminase	-2.65	2.09E-09
NM_001013745	***Zfp57***	zinc finger protein 57	-2.65	5.76E-09
NM_001025586	***Nr2c2ap***	nuclear receptor 2C2-associated protein	-2.66	2.75E-08
NM_029508	***Pcgf5***	polycomb group ring finger 5	-2.66	4.87E-11
NM_001163493	***Stard13***	StAR-related lipid transfer (START) domain containing 13	-2.67	3.30E-09
NM_172525	***Arhgap29***	Rho GTPase activating protein 29	-2.67	1.18E-07
NM_183199	***Usp44***	ubiquitin specific peptidase 44	-2.67	2.47E-07
NM_029352	***Dusp9***	dual specificity phosphatase 9	-2.67	1.25E-09
NM_173788	***Npr2***	natriuretic peptide receptor 2	-2.68	1.25E-09
NM_175089	***Nek1***	NIMA (never in mitosis gene a)-related expressed kinase 1	-2.68	2.28E-07
NM_057172	***Fubp1***	far upstream element (FUSE) binding protein 1	-2.68	4.09E-11
ENSMUST00000099268	***Gm10752***	predicted gene 10752	-2.68	1.60E-07
NM_153582	***Cmtm4***	CKLF-like MARVEL transmembrane domain containing 4	-2.69	2.35E-09
NM_026301	***Rnf125***	ring finger protein 125	-2.69	1.96E-05
NM_153060	***Spns2***	spinster homolog 2 (Drosophila)	-2.69	3.40E-10
NM_198247	***Sertad4***	SERTA domain containing 4	-2.69	2.57E-10
NM_026038	***2810055F11Rik***	RIKEN cDNA 2810055F11 gene	-2.69	5.98E-08
NM_019410	***Pfn2***	profilin 2	-2.70	3.17E-07
NR_028527	***Snord52***	small nucleolar RNA, C/D box 52	-2.70	1.99E-07
NM_172514	***Tmem71***	transmembrane protein 71	-2.71	1.72E-08
NM_010762	***Mal***	myelin and lymphocyte protein, T-cell differentiation protein	-2.72	4.96E-05
NM_009194	***Slc12a2***	solute carrier family 12, member 2	-2.72	4.05E-11
NM_009627	***Adm***	adrenomedullin	-2.72	2.39E-07
NR_028478	***Snora75***	small nucleolar RNA, H/ACA box 75	-2.73	7.25E-06
NM_001081332	***Slc9a5***	solute carrier family 9 (sodium/hydrogen exchanger), member 5	-2.73	7.52E-10
AF357367	***Rpl23a***	ribosomal protein L23a	-2.73	1.33E-06
NM_027260	***Vrk2***	vaccinia related kinase 2	-2.73	7.61E-08
NM_001163471	***Hectd2***	HECT domain containing 2	-2.74	2.53E-06
NM_013511	***Epb4.1l2***	erythrocyte protein band 4.1-like 2	-2.74	2.57E-10
NM_001017427	***Rasef***	RAS and EF hand domain containing	-2.75	1.01E-06
NM_033320	***Glce***	glucuronyl C5-epimerase	-2.75	1.34E-09
NR_033168	***Snora28***	small nucleolar RNA, H/ACA box 28	-2.77	1.95E-05
NM_019807	***Acpp***	acid phosphatase, prostate	-2.77	4.86E-09
BC115525	***9230110C19Rik***	RIKEN cDNA 9230110C19 gene	-2.78	1.01E-07
NM_019698	***Aldh18a1***	aldehyde dehydrogenase 18 family, member A1	-2.78	5.73E-11
NM_023580	***Epha1***	Eph receptor A1	-2.79	3.91E-08
NM_176838	***Esrp2***	epithelial splicing regulatory protein 2	-2.79	5.34E-10
NM_009969	***Csf2***	colony stimulating factor 2 (granulocyte-macrophage)	-2.80	9.43E-08
NM_001173547	***Taz***	tafazzin	-2.80	3.98E-10
NM_180600	***Ube2q2***	ubiquitin-conjugating enzyme E2Q (putative) 2	-2.80	1.12E-10
NM_029852	***Ccdc41***	coiled-coil domain containing 41	-2.80	1.42E-08
NM_153783	***Paox***	polyamine oxidase (exo-N4-amino)	-2.81	2.31E-10
ENSMUST00000056686	***2210011C24Rik***	RIKEN cDNA 2210011C24 gene	-2.81	1.86E-08
NM_009140	***Cxcl2***	chemokine (C-X-C motif) ligand 2	-2.82	3.26E-06
NM_018802	***Syt8***	synaptotagmin VIII	-2.82	5.01E-10
NM_176993	***Mpzl3***	myelin protein zero-like 3	-2.83	2.83E-10
NM_011579	***Tgtp1***	T-cell specific GTPase 1	-2.85	1.76E-09
NM_013717	***B9d1***	B9 protein domain 1	-2.85	6.58E-09
NM_023049	***Asb2***	ankyrin repeat and SOCS box-containing 2	-2.86	1.47E-07
NM_133220	***Sgk3***	serum/glucocorticoid regulated kinase 3	-2.86	1.91E-10
NM_023224	***Cblc***	Casitas B-lineage lymphoma c	-2.87	1.17E-09
NR_002899	***Snora70***	small nucleolar RNA, H/ACA box 70	-2.87	2.01E-07
NM_201352	***Gdpd5***	glycerophosphodiester phosphodiesterase domain containing 5	-2.87	1.79E-11
NR_027821	***1810032O08Rik***	RIKEN cDNA 1810032O08 gene	-2.89	2.34E-07

NM_027419	***2810408A11Rik***	RIKEN cDNA 2810408A11 gene	-2.89	1.39E-09
NM_172839	***Ccnj***	cyclin J	-2.90	7.47E-10
BC056635	***5730469M10Rik***	RIKEN cDNA 5730469M10 gene	-2.90	1.53E-11
NM_020030	***Raet1d***	retinoic acid early transcript delta	-2.90	7.97E-07
NR_028574	***Snhg8***	small nucleolar RNA host gene 8	-2.90	1.02E-05
NM_001033210	***Pls1***	plastin 1 (I-isoform)	-2.92	6.20E-10
NM_007400	***Adam12***	a disintegrin and metallopeptidase domain 12 (meltrin alpha)	-2.92	2.23E-09
NM_008357	***Il15***	interleukin 15	-2.93	1.38E-08
NM_029653	***Dapk1***	death associated protein kinase 1	-2.94	6.52E-08
NM_028567	***1700094D03Rik***	RIKEN cDNA 1700094D03 gene	-2.94	5.76E-09
NR_028563	***Snord96a***	small nucleolar RNA, C/D box 96A	-2.94	3.51E-06
NM_009728	***Atp10a***	ATPase, class V, type 10A	-2.95	8.54E-11
NM_172865	***Manea***	mannosidase, endo-alpha	-2.95	8.51E-12
NM_133774	***Stard4***	StAR-related lipid transfer (START) domain containing 4	-2.95	1.45E-08
NM_001024617	***Inpp4b***	inositol polyphosphate-4-phosphatase, type II	-2.95	4.74E-11
NM_178793	***Ccbe1***	collagen and calcium binding EGF domains 1	-2.96	3.43E-12
NM_001081344	***Stxbp5***	syntaxin binding protein 5 (tomosyn)	-2.97	1.77E-09
NR_030779	***Ankrd10***	ankyrin repeat domain 10	-2.97	3.90E-10
NM_007936	***Epha4***	Eph receptor A4	-2.98	2.11E-09
NM_009402	***Pglyrp1***	peptidoglycan recognition protein 1	-2.99	6.29E-08
NM_001164311	***Loxl4***	lysyl oxidase-like 4	-3.00	3.73E-10
NM_025343	***Rmnd1***	required for meiotic nuclear division 1 homolog (S. cerevisiae)	-3.00	1.43E-11
NM_172711	***Guf1***	GUF1 GTPase homolog (S. cerevisiae)	-3.01	2.38E-09
NR_002891	***Gm5512***	required for meiotic nuclear division 1 pseudogene	-3.01	2.09E-11
NM_133992	***Pan2***	PAN2 polyA specific ribonuclease subunit homolog (S. cerevisiae)	-3.05	1.40E-11
NM_016917	***Slc40a1***	solute carrier family 40 (iron-regulated transporter), member 1	-3.05	1.21E-07
NR_015483	***2610203C20Rik***	RIKEN cDNA 2610203C20 gene	-3.05	2.76E-09
NM_001099632	***Rnf39***	ring finger protein 39	-3.06	3.59E-11
NM_010241	***Aktip***	thymoma viral proto-oncogene 1 interacting protein	-3.08	3.07E-12
NM_145595	***Cbr4***	carbonyl reductase 4	-3.08	8.30E-10
NM_053122	***Immp2l***	IMP2 inner mitochondrial membrane peptidase-like (S. cerevisiae)	-3.09	1.13E-08
NM_025512	***Zfand1***	zinc finger, AN1-type domain 1	-3.09	1.66E-08
NR_015505	***6720401G13Rik***	RIKEN cDNA 6720401G13 gene	-3.09	7.25E-07
NM_028770	***Krt80***	keratin 80	-3.09	9.46E-12
ENSMUST00000079237	***Zfp125***	zinc finger protein 125	-3.11	9.82E-05
NM_015760	***Nox4***	NADPH oxidase 4	-3.12	6.07E-09
NM_172411	***2310007B03Rik***	RIKEN cDNA 2310007B03 gene	-3.15	2.01E-08
BC032970	***2810026P18Rik***	RIKEN cDNA 2810026P18 gene	-3.15	3.59E-09
NM_013912	***Apln***	apelin	-3.15	3.32E-10
NM_172524	***Nipal4***	NIPA-like domain containing 4	-3.17	4.50E-10
NM_175937	***Cpeb2***	cytoplasmic polyadenylation element binding protein 2	-3.17	6.62E-10
NR_002840	***Gas5***	growth arrest specific 5	-3.18	2.48E-07
NR_028572	***Snora43***	small nucleolar RNA, H/ACA box 43	-3.19	1.03E-08
NM_139232	***Fgd4***	FYVE, RhoGEF and PH domain containing 4	-3.20	8.01E-09
NM_026673	***Apoo***	apolipoprotein O	-3.21	4.52E-08
NM_027860	***0610010F05Rik***	RIKEN cDNA 0610010F05 gene	-3.21	1.83E-08
NM_010680	***Lama3***	laminin, alpha 3	-3.22	1.48E-08
NM_007562	***Bnc1***	basonuclin 1	-3.23	1.81E-10
NM_008655	***Gadd45b***	growth arrest and DNA-damage-inducible 45 beta	-3.25	1.14E-10
NM_016718	***Ninj2***	ninjurin 2	-3.26	7.61E-09
NM_053115	***Acox2***	acyl-Coenzyme A oxidase 2, branched chain	-3.26	1.39E-09
NM_212447	***Marveld3***	MARVEL (membrane-associating) domain containing 3	-3.26	1.77E-10
NM_030697	***Kank3***	KN motif and ankyrin repeat domains 3	-3.26	1.69E-11
NM_025705	***Dcbld1***	discoidin, CUB and LCCL domain containing 1	-3.31	6.04E-10
NM_211138	***Pcyt1b***	phosphate cytidylyltransferase 1, choline, beta isoform	-3.31	2.04E-10
NR_001579	***Terc***	telomerase RNA component	-3.33	1.50E-10
NM_029662	***Mfsd2a***	major facilitator superfamily domain containing 2A	-3.33	1.49E-11
NM_181323	***Cwh43***	cell wall biogenesis 43 C-terminal homolog (S. cerevisiae)	-3.33	1.67E-09
NM_174857	***Mamdc2***	MAM domain containing 2	-3.36	6.30E-09
NM_007963	***Mecom***	MDS1 and EVI1 complex locus	-3.37	1.27E-09
NM_023158	***Cxcl16***	chemokine (C-X-C motif) ligand 16	-3.38	4.02E-11
NR_028078	***Snora21***	small nucleolar RNA, H/ACA box 21	-3.38	1.34E-06
ENSMUST00000099340	***5830416P10Rik***	RIKEN cDNA 5830416P10 gene	-3.39	7.00E-10
NM_001001488	***Atp8b1***	ATPase, class I, type 8B, member 1	-3.39	1.59E-08
NM_133214	***BC017612***	cDNA sequence BC017612	-3.43	1.38E-08
NM_126166	***Tlr3***	toll-like receptor 3	-3.46	7.25E-11
NM_027168	***Hddc2***	HD domain containing 2	-3.49	4.05E-11
NM_009397	***Tnfaip3***	tumor necrosis factor, alpha-induced protein 3	-3.50	1.03E-11
NM_145360	***Idi1***	isopentenyl-diphosphate delta isomerase	-3.53	8.76E-09
NM_019686	***Cib2***	calcium and integrin binding family member 2	-3.54	4.44E-11
NM_018747	***Akap7***	A kinase (PRKA) anchor protein 7	-3.56	7.74E-11
NM_033608	***Igsf9***	immunoglobulin superfamily, member 9	-3.58	1.52E-10
NM_016851	***Irf6***	interferon regulatory factor 6	-3.58	3.20E-12
AY223547	***Pnet-ps***	prenatal ethanol induced mRNA, pseudogene	-3.58	8.74E-08

NM_010631	***Kifc3***	kinesin family member C3	-3.61	5.62E-11
NM_026604	***Fam135a***	family with sequence similarity 135, member A	-3.64	8.75E-10
NM_008940	***Klk8***	kallikrein related-peptidase 8	-3.65	4.74E-12
NM_026163	***Pkp2***	plakophilin 2	-3.65	1.90E-10
NM_001164201	***Lass3***	LAG1 homolog, ceramide synthase 3	-3.65	1.95E-10
NM_001100449	***Taf4b***	TAF4B RNA polymerase II, TATA box binding protein (TBP)-associated factor	-3.67	1.32E-09
NM_201600	***Myo5b***	myosin VB	-3.68	1.66E-10
NM_026788	***Mthfd2l***	methylenetetrahydrofolate dehydrogenase (NADP+ dependent) 2-like	-3.69	5.02E-05
NM_001177351	***AW112010***	expressed sequence AW112010	-3.70	1.59E-08
NM_031252	***Il23a***	interleukin 23, alpha subunit p19	-3.70	2.96E-12
NM_001039698	***Rhox4g***	reproductive homeobox 4G	-3.72	9.21E-09
NM_007974	***F2rl1***	coagulation factor II (thrombin) receptor-like 1	-3.73	4.23E-11
NM_201236	***Rhox4e***	reproductive homeobox 4E	-3.73	1.49E-08
NM_020010	***Cyp51***	cytochrome P450, family 51	-3.74	3.81E-09
NM_018857	***Msln***	mesothelin	-3.74	9.62E-13
NM_001002897	***Atg9b***	ATG9 autophagy related 9 homolog B (S. cerevisiae)	-3.77	2.64E-08
NM_001114312	***4930506M07Rik***	RIKEN cDNA 4930506M07 gene	-3.78	3.33E-10
NM_023113	***Aspa***	aspartoacylase	-3.80	6.14E-09
NM_009323	***Tbx15***	T-box 15	-3.80	1.47E-11
NM_144795	***Pycr1***	pyrroline-5-carboxylate reductase 1	-3.83	6.77E-10
NM_198657	***Gm5148***	predicted gene 5148	-3.84	3.27E-08
NM_001081079	***Ogfrl1***	opioid growth factor receptor-like 1	-3.86	2.40E-09
NM_001145162	***Ube2ql1***	ubiquitin-conjugating enzyme E2Q family-like 1	-3.86	2.67E-10
NM_001039696	***Rhox4f***	reproductive homeobox 4F	-3.87	2.13E-09
NM_027756	***Mfap3l***	microfibrillar-associated protein 3-like	-3.87	1.40E-07
NM_001081005	***1500012F01Rik***	RIKEN cDNA 1500012F01 gene	-3.87	2.64E-11
NM_028002	***Dus4l***	dihydrouridine synthase 4-like (S. cerevisiae)	-3.89	2.18E-10
NM_178407	***Arap2***	ArfGAP with RhoGAP domain, ankyrin repeat and PH domain 2	-3.91	6.18E-10
NM_026770	***Cgref1***	cell growth regulator with EF hand domain 1	-3.91	1.47E-10
NM_009601	***Chrnb1***	cholinergic receptor, nicotinic, beta polypeptide 1 (muscle)	-3.94	2.13E-11
NM_025921	***2610002M06Rik***	RIKEN cDNA 2610002M06 gene	-3.95	7.43E-11
NM_029413	***Morc4***	microrchidia 4	-4.00	1.32E-12
NM_008330	***Ifi47***	interferon gamma inducible protein 47	-4.01	8.28E-10
NM_010945	***Nsmaf***	neutral sphingomyelinase (N-SMase) activation associated factor	-4.03	2.82E-11
NM_133731	***Prss22***	protease, serine, 22	-4.03	3.10E-09
NM_170779	***Wwc1***	WW, C2 and coiled-coil domain containing 1	-4.06	3.98E-13
NM_178381	***Ano9***	anoctamin 9	-4.06	1.71E-09
NR_028552	***Snord58b***	small nucleolar RNA, C/D box 58B	-4.06	2.38E-06
NM_028924	***Tc2n***	tandem C2 domains, nuclear	-4.06	1.33E-08
NM_001033498	***Gramd2***	GRAM domain containing 2	-4.06	7.95E-10
NM_010281	***Ggh***	gamma-glutamyl hydrolase	-4.09	1.31E-10
NR_002900	***Snora69***	small nucleolar RNA, H/ACA box 69	-4.10	4.88E-09
NM_024204	***Ankrd22***	ankyrin repeat domain 22	-4.10	1.41E-08
NM_008635	***Mtap7***	microtubule-associated protein 7	-4.11	2.28E-07
NM_001113460	***Tec***	tec protein tyrosine kinase	-4.12	3.81E-09
NM_173014	***Lpcat2***	lysophosphatidylcholine acyltransferase 2	-4.14	1.91E-11
NM_025303	***Stau2***	staufen (RNA binding protein) homolog 2 (Drosophila)	-4.16	8.57E-12
NM_008396	***Itga2***	integrin alpha 2	-4.18	1.49E-11
NM_001007460	***Zdhhc23***	zinc finger, DHHC domain containing 23	-4.22	2.36E-10
NM_021300	***Rhox4b***	reproductive homeobox 4B	-4.25	9.72E-09
NM_011176	***St14***	suppression of tumorigenicity 14 (colon carcinoma)	-4.26	4.34E-12
NM_011600	***Tle4***	transducin-like enhancer of split 4, homolog of Drosophila E(spl)	-4.27	6.65E-10
NM_138750	***Prom2***	prominin 2	-4.29	2.07E-09
NM_025468	***Sec11c***	SEC11 homolog C (S. cerevisiae)	-4.29	2.40E-12
NM_007557	***Bmp7***	bone morphogenetic protein 7	-4.33	1.06E-11
NM_001038999	***Atp8a1***	ATPase, aminophospholipid transporter (APLT), class I, type 8A, member 1	-4.34	6.74E-10
NM_023842	***Dsp***	desmoplakin	-4.36	4.08E-12
NM_025436	***Sc4mol***	sterol-C4-methyl oxidase-like	-4.37	4.16E-12
NM_025519	***Chmp4c***	chromatin modifying protein 4C	-4.41	2.57E-10
NM_021436	***Tmeff1***	transmembrane protein with EGF-like and two follistatin-like domains 1	-4.44	8.77E-12
NM_207623	***Rnf138***	ring finger protein 138	-4.45	8.95E-09
NM_009075	***Rpia***	ribose 5-phosphate isomerase A	-4.48	3.43E-12
NM_173866	***Gpt2***	glutamic pyruvate transaminase (alanine aminotransferase) 2	-4.52	1.53E-08
NM_011057	***Pdgfb***	platelet derived growth factor, B polypeptide	-4.52	3.98E-12
NM_138653	***Bspry***	B-box and SPRY domain containing	-4.53	2.61E-10
NM_001100458	***Fam169a***	family with sequence similarity 169, member A	-4.55	4.91E-10
AK145170	***G930009F23Rik***	RIKEN cDNA G930009F23 gene	-4.55	2.05E-12
NM_031880	***Tnk1***	tyrosine kinase, non-receptor, 1	-4.58	1.03E-12
ENSMUST00000029268	***1810062G17Rik***	RIKEN cDNA 1810062G17 gene	-4.59	3.48E-09
NM_028372	***Mblac2***	metallo-beta-lactamase domain containing 2	-4.60	5.04E-10
NM_021409	***Pard6b***	par-6 (partitioning defective 6) homolog beta (C. elegans)	-4.62	3.43E-12
NM_053087	***Epgn***	epithelial mitogen	-4.64	6.37E-09
NM_178676	***Entpd3***	ectonucleoside triphosphate diphosphohydrolase 3	-4.65	9.72E-10
NR_028549	***Snord37***	small nucleolar RNA, C/D box 37	-4.68	4.88E-07

NM_010634	***Fabp5***	fatty acid binding protein 5, epidermal	-4.69	7.64E-11
NM_008126	***Gjb3***	gap junction protein, beta 3	-4.71	8.54E-13
NR_004445	***Snord22***	small nucleolar RNA, C/D box 22	-4.71	5.99E-09
NM_001038602	***Marveld2***	MARVEL (membrane-associating) domain containing 2	-4.73	2.96E-11
NM_010164	***Eya1***	eyes absent 1 homolog (Drosophila)	-4.74	1.03E-12
NM_018815	***Nup210***	nucleoporin 210	-4.74	1.43E-11
NM_173434	***9930111J21Rik2***	RIKEN cDNA 9930111J21 gene 2	-4.83	1.85E-08
BC120577	***Fam83b***	family with sequence similarity 83, member B	-4.83	1.72E-09
NM_029909	***C330018D20Rik***	RIKEN cDNA C330018D20 gene	-4.86	4.10E-07
NM_029031	***Shpk***	sedoheptulokinase	-4.87	2.41E-11
NM_133712	***Klk10***	kallikrein related-peptidase 10	-4.89	2.50E-12
NM_001039688	***Rhox4a***	reproductive homeobox 4A	-4.91	4.90E-09
NM_001039689	***Rhox4c***	reproductive homeobox 4C	-4.92	4.11E-09
NM_153546	***Mboat1***	membrane bound O-acyltransferase domain containing 1	-4.94	2.28E-12
NM_001004363	***Nuak1***	NUAK family, SNF1-like kinase, 1	-4.94	3.12E-11
AF357393	***Rps12***	ribosomal protein S12	-4.99	4.36E-09
NM_173870	***Mgat4a***	mannoside acetylglucosaminyltransferase 4, isoenzyme A	-5.12	3.39E-09
NM_008862	***Pkia***	protein kinase inhibitor, alpha	-5.15	1.63E-08
NM_008471	***Krt19***	keratin 19	-5.18	1.18E-11
NM_007914	***Ehf***	ets homologous factor	-5.18	2.52E-08
NM_013468	***Ankrd1***	ankyrin repeat domain 1 (cardiac muscle)	-5.26	2.81E-11
NM_001159564	***Itgb6***	integrin beta 6	-5.34	1.67E-10
NM_133832	***Rdh10***	retinol dehydrogenase 10 (all-trans)	-5.43	3.59E-13
NM_008813	***Enpp1***	ectonucleotide pyrophosphatase/phosphodiesterase 1	-5.44	1.06E-11
NM_019538	***Plac1***	placental specific protein 1	-5.46	1.17E-10
NM_029821	***1190003J15Rik***	RIKEN cDNA 1190003J15 gene	-5.52	7.35E-12
NM_022995	***Pmepa1***	prostate transmembrane protein, androgen induced 1	-5.57	1.80E-13
NM_023663	***Ripk4***	receptor-interacting serine-threonine kinase 4	-5.58	6.13E-11
NM_026517	***Rpl22l1***	ribosomal protein L22 like 1	-5.61	4.53E-08
NM_009910	***Cxcr3***	chemokine (C-X-C motif) receptor 3	-5.62	7.49E-11
NM_029000	***Gvin1***	GTPase, very large interferon inducible 1	-5.63	1.71E-08
AK172683	***Gm8995***	predicted gene 8995	-5.64	5.01E-11
NM_001081162	***Slc4a11***	solute carrier family 4, sodium bicarbonate transporter-like, member 11	-5.75	6.13E-12
NM_027571	***P2ry12***	purinergic receptor P2Y, G-protein coupled 12	-5.88	8.06E-09
NM_008009	***Fgfbp1***	fibroblast growth factor binding protein 1	-5.91	2.83E-09
NM_022032	***Perp***	PERP, TP53 apoptosis effector	-5.93	5.06E-14
NM_026931	***1810011O10Rik***	RIKEN cDNA 1810011O10 gene	-5.94	2.91E-10
NM_023716	***Tubb2b***	tubulin, beta 2B	-5.94	6.27E-11
NM_001111119	***Ccnb1ip1***	cyclin B1 interacting protein 1	-5.94	9.28E-12
NM_016958	***Krt14***	keratin 14	-5.98	8.77E-12
NM_011845	***Mid2***	midline 2	-6.08	2.61E-11
NM_001033325	***A630095E13Rik***	RIKEN cDNA A630095E13 gene	-6.12	6.82E-10
NM_212444	***Gyk***	glycerol kinase	-6.15	6.51E-10
NM_153526	***Insig1***	insulin induced gene 1	-6.17	1.00E-12
NM_017379	***Tuba8***	tubulin, alpha 8	-6.25	4.27E-10
NM_025961	***Gatm***	glycine amidinotransferase (L-arginine:glycine amidinotransferase)	-6.26	1.88E-13
NM_019737	***B4galt6***	UDP-Gal:betaGlcNAc beta 1,4-galactosyltransferase, polypeptide 6	-6.42	6.51E-10
NM_013560	***Hspb1***	heat shock protein 1	-6.58	1.78E-11
NM_001110300	***Ap1m2***	adaptor protein complex AP-1, mu 2 subunit	-6.66	3.72E-10
NM_008484	***Lamb3***	laminin, beta 3	-6.81	2.61E-11
NM_001161548	***Tmem184a***	transmembrane protein 184a	-7.05	3.98E-13
NM_009985	***Ctsw***	cathepsin W	-7.16	1.48E-11
NM_012009	***Sh2d1b1***	SH2 domain protein 1B1	-7.19	1.75E-09
NM_026172	***Decr1***	2,4-dienoyl CoA reductase 1, mitochondrial	-7.24	6.84E-10
NM_145523	***Gca***	grancalcin	-7.56	4.22E-11
NM_010153	***Erbb3***	v-erb-b2 erythroblastic leukemia viral oncogene homolog 3 (avian)	-7.67	6.23E-10
NM_198884	***B4galnt3***	beta-1,4-N-acetyl-galactosaminyl transferase 3	-7.80	4.50E-10
NM_018754	***Sfn***	stratifin	-7.95	1.24E-11
NR_030719	***Gm8979***	very large inducible GTPase 1 pseudogene	-8.12	5.00E-08
NM_011267	***Rgs16***	regulator of G-protein signaling 16	-8.21	1.89E-12
BC132471	***Gm129***	predicted gene 129	-8.47	2.19E-13
NM_011725	***Xlr***	X-linked lymphocyte-regulated complex	-8.60	5.90E-08
NM_026496	***Grhl2***	grainyhead-like 2 (Drosophila)	-9.11	6.19E-13
NM_178936	***Tmem56***	transmembrane protein 56	-9.63	3.17E-12
NM_007883	***Dsg2***	desmoglein 2	-9.74	1.65E-11
NM_009527	***Wnt7a***	wingless-related MMTV integration site 7A	-9.83	9.64E-14
NM_008380	***Inhba***	inhibin beta-A	-9.84	9.64E-14
NM_133681	***Tspan1***	tetraspanin 1	-9.84	9.77E-13
NM_001093754	***Dennd2d***	DENN/MADD domain containing 2D	-9.93	8.32E-10
NM_010217	***Ctgf***	connective tissue growth factor	-9.97	1.63E-14
NM_198014	***Slain1***	SLAIN motif family, member 1	-10.25	3.07E-12
NM_010346	***Grb7***	growth factor receptor bound protein 7	-10.32	1.03E-13
NM_010554	***Il1a***	interleukin 1 alpha	-10.63	2.49E-13
NM_172752	***Sorbs2***	sorbin and SH3 domain containing 2	-10.69	8.93E-14

NM_010720	***Lipg***	lipase, endothelial	-11.19	3.71E-13
NM_021509	***Moxd1***	monooxygenase, DBH-like 1	-11.53	3.59E-13
NM_008728	***Npr3***	natriuretic peptide receptor 3	-11.66	9.63E-12
NM_010483	***Htr5b***	5-hydroxytryptamine (serotonin) receptor 5B	-11.78	3.13E-12
NM_008590	***Mest***	mesoderm specific transcript	-12.19	4.74E-12
NM_009829	***Ccnd2***	cyclin D2	-12.50	4.27E-13
NM_001130174	***Tnnt2***	troponin T2, cardiac	-12.51	4.63E-14
NM_053095	***Il24***	interleukin 24	-12.85	9.64E-14
NM_001163136	***Macc1***	metastasis associated in colon cancer 1	-13.16	6.45E-13
NM_145078	***2610305D13Rik***	RIKEN cDNA 2610305D13 gene	-13.16	9.23E-11
NM_016887	***Cldn7***	claudin 7	-13.76	8.77E-12
NM_008485	***Lamc2***	laminin, gamma 2	-14.13	6.28E-13
NM_009864	***Cdh1***	cadherin 1	-14.35	5.06E-14
NM_013512	***Epb4.1l4a***	erythrocyte protein band 4.1-like 4a	-14.44	3.98E-13
NM_181820	***Tmc4***	transmembrane channel-like gene family 4	-15.82	6.29E-15
BC038020	***Fam111a***	family with sequence similarity 111, member A	-16.22	1.73E-12
ENSMUST00000025390	***2310002L13Rik***	RIKEN cDNA 2310002L13 gene	-17.59	7.63E-13
NM_008532	***Epcam***	epithelial cell adhesion molecule	-18.20	1.35E-15
NM_009902	***Cldn3***	claudin 3	-19.97	1.32E-12
NM_007962	***Mpzl2***	myelin protein zero-like 2	-20.95	1.47E-14
NM_133664	***Lad1***	ladinin	-21.07	1.46E-13
NM_020047	***Tacstd2***	tumor-associated calcium signal transducer 2	-21.18	4.56E-12
NM_008430	***Kcnk1***	potassium channel, subfamily K, member 1	-23.10	9.62E-13
NM_145394	***Slc44a3***	solute carrier family 44, member 3	-24.54	1.04E-12
NM_198029	***Fermt1***	fermitin family homolog 1 (Drosophila)	-25.11	4.70E-13
NM_009257	***Serpinb5***	serine (or cysteine) peptidase inhibitor, clade B, member 5	-27.39	4.93E-12
NM_008879	***Lcp1***	lymphocyte cytosolic protein 1	-27.70	1.05E-12
NM_016899	***Rab25***	RAB25, member RAS oncogene family	-39.84	3.07E-14
NM_194055	***Esrp1***	epithelial splicing regulatory protein 1	-44.13	8.31E-13
NM_025452	***Tmem54***	transmembrane protein 54	-44.20	5.05E-14
NM_172880	***Tmprss11e***	transmembrane protease, serine 11e	-50.26	4.13E-15
NM_033073	***Krt7***	keratin 7	-51.07	7.16E-16
NM_009903	***Cldn4***	claudin 4	-57.19	2.61E-13
NM_021540	***Rnf130***	ring finger protein 130	-57.59	1.33E-15
NM_178920	***Mal2***	mal, T-cell differentiation protein 2	-66.67	7.16E-16

Supplementary materials and methods

Cell culture. The 4T1 murine breast cancer cell line was kindly provided by Dr Fred R. Miller (Michigan Cancer Foundation, Detroit, MI, USA). 4T1 cells were cultured in high glucose DMEM supplemented with 10% heat-inactivated FBS, 1% penicillin–streptomycin (P/S, from Life Technologies – Invitrogen) and 1% Non-Essential Amino Acid (Gibco). The D2A1 and D2.0R murine breast cancer cell lines were kindly provided by Dr Jonathan Sleeman (Medical Faculty at Heidelberg University, Mannheim, Germany). D2A1and D2.0R cells were cultured in high glucose DMEM supplemented with 10% heat-inactivated FBS, 1% P/S. The human breast cancer cell lines MDA-MB-231 and MDA-MB-468 were purchased form ATCC and cultured in DMEM supplemented with 10% heat-inactivated FBS, 1% P/S. To test the effect of chemotherapy drugs on cells, $5x10^5$ were cultured overnight in 6 well plates in culture medium and the day after, we added the drugs at indicated concentrations (Figure 4). Recombinant mouse IFN-β (Biolegend) was used at 50 ng/ml. IFNAR1 blocking antibody (clone MAR1-5A3, from BioXcell) and IgG control (clone MOPC-21, from BioXcell) were used at a concentration of 20 μg/ml for 24h.

Antibodies. In general, 10^6 cells were stained in round bottom 96 well plates. Surface antibodies were diluted with staining buffer (1% FBS, 1mM EDTA, and 0.02% NaN3 in PBS) and added to cells at 20 µl per sample. For intracellular stains cells were permeabilized using Biolegend's kit, according to the manufacturer's protocol. The following anti-mouse antibodies were used following manufacturer's instructions: anti-CD16/CD32 Fc blocking antibody (BD Biosciences), CD11b-PeCy7 (clone M1/70, Biolegend), Ly6C-FITC (clone HK1.4, Biolegend), Gr1-Pacific Blue (clone RB6-8C5, Biolegend), Ly6G-APC (clone 1A8, Bio Legend), CD45 PE (clone 30-F11, BD

Biosciences), CD11c APC-Cy7 (clone N418, eBiosciences, ThermoFischer Scientific), CD4-FITC (clone RM4-5, Biolegend), CD8-PE (clone 53-6.7, eBioscience), B220-APC (clone RA3-6B2, eBiosciences), CD49b Pacific blue (Clone DX5, eBiosciences), FasL (CD178)-PeCy7 (clone MFL3, eBioscience), Granzyme B-eFluor450 (clone NGZB, eBioscience) and IFN-γ FITC (clone 4S.B3 , eBioscience). FITC-Ki67 antibody (Ab27619) was purchased from Abcam.

***In vitro* cell growth assay**. Cells were collected and seeded in tissue culture 96-well-plates (Costar) at 1,000 cells/well. Cells were grown in complete medium for 24, 36, 72 and 96 hours. At each time point cells were washed once with PBS, then fixed with 4% PFA and stained with 0.5% crystal violet solution for 0.5 hours. The stained cells were gently washed with deionized water to remove the extra dye, and air-dried overnight at room temperature. After resolving the dye with crystal violet eluting buffer (70% ethanol and 1% acetic acid), cell viability was assessed by reading the absorbance at 595 nm wavelength in a multiwell plate reader (Modulus II microplate reader, Turner Biosystems). Results were analyzed by Prism (Graph pad software, Inc., La Jolla, CA, US) expressed as mean values of optical density (OD) of octuplet determinations ± SEM.

Cell proliferation assay. Cells were collected and prepared in a proper concentration (10^6 cells per ml of complete medium). They were incubated with 10 µM of Green CMFDA (Life Technologies – Invitrogen) for 45 min in 37°C, then washed to remove the excess of dye and put in culture. Dye fluorescence intensity was detected by flow cytometry analysis 24 and 72 hours later.

In vitro cytotoxic assay. Tumor cells were plated at a concentration of 1000 cell/well into 96-wells plate. The following day, a series of concentrations of the different drugs were supplemented to the culture medium. Untreated control cells were kept in normal culture medium. Cell viability of each well was assessed with crystal violet staining 48 hours after treatment, as described above. Results were analyzed by Prism software by a non-linear regression analysis and expressed as relative cell viability compared with non-treated control. The 50% or 85% maximum inhibition concentrations (IC50, IC85) were used to determine the drug-resistant ability of treated cells.

Cell cycle analysis. For Ki67 immune-fluorescent staining, 2500 cells per well were plated in Nunc Lab-Tek Chamber slide (ThermoFisher) and cultured overnight in complete medium. The cells were washed once with PBS and then fixed with 4% PFA at RT for 1 hour. After washing with PBS, cells were permeabilized with 0.3% Trition X-100 (Sigma Aldrich) in PBS (PBST) for 1 hour. The unspecific staining was blocked with 3% BSA (Sigma Aldrich) in PBST. The FITC conjugated Ki67 antibody (1:1000) was diluted in blocking buffer and incubated at 4º C overnight. Thereafter, the cells were washed with PBST and counterstained with 300 nM DAPI (Life Technology) and mounted with VECTASHIELD mounting medium (H-1000, Vector Laboratories). Image were taken with Leica TCS SP5 and the cell numbers were counted manually. Results were analyzed by Prism. Click-iT Edu Alexa Fluor 488 Flow Cytometry Assay Kit (Life Technologies) were used to assess the cell proliferation status by flow cytometry. The cells were incubated with 10 µM EdU in complete medium for 1 hour, and staining was performed according to manufacturer’s instructions. The DNA content were measured by Propidium iodide (PI) staining (Life Technologies). Data acquisition was performed using FACSCalibur flow cytometer from BD Bioscience and data analyzed by FlowJo v10.0.7.

Apoptosis analysis. Cell apoptosis detection was performed using a PE-Annexin V Apoptosis Detection Kit (559763, BD Bioscience) according to manufacturer's instructions. For detection of caspase activation, we used also the CellEvent™ Caspase-3/7 Green Flow Cytometry Assay Kit (Life Technologies - Invitrogen C10427). Data were acquired using the FACSCalibur flow cytometer from BD Bioscience and data analyzed by FlowJo v10.0.7.

Type I IFN measurement by ELISA. $5x10^5$ tumor cells were cultured overnight in 6 well plates and then treated or not for 24h. Supernatants were collected and stored at -80°C. For measurement of type I IFN in serum, 50 µM of serum were used. IFN-α and IFN-β ELISA was performed according to manufacturer's instructions (R&D Systems).

Real-time (RT) qPCR and primers. Changes in mRNA expression levels were determined by semi-quantitative real-time qPCR. RNA samples were obtained from adherent cells using RNeasy kit from QIAGEN according to manufacturer's instructions. From each sample, 1 µg RNA was retro-transcribed using SuperScript II Reverse Transcriptase kit (Life Technologies – Invitrogen), according to manufacturer's instructions. The reactions were performed in a StepOnePlus™ thermocycler (Applied Biosystems. Life Technologies – Invitrogen) using the KapaSYBR® FAST SYBR Green Master Mix (Kapa Biosystems). Each reaction was performed in triplicate and values were normalized to murine 36B4 housekeeping gene. The comparative C_t method was used to calculate the difference of gene expression between samples. The following murine-specific primers (Microsynth AG) were used:

36B4 forward, 5’-GTGTGTCTGCAGATCGGGTAC-3’, reverse, 5’-CAGATGGATCAGCCAGGAAG-3’ ; IRF-7 forward, 5’-TGATCCGCATAAGGTGTACG-3’, reverse, 5’-AGCATTGCTGAGGCTCACTT-3’ ; IRF-9 forward, 5’-TTCCTGGAGCATCAACTTCC-3’, reverse 5’-CAGTCTAGGCTGTGCACCTG-3’ ; STAT1 forward, 5’-AAGATTTTGGAAAATGCCCA-3’, reverse, 5’-TGCACATGACTTGATCCTTCA-3’ ; STAT2 forward, 5’-CGCTTGGAGAATTGGAAGTT-3’, reverse, 5’-GCTGTCAAGGTTCTGCAACA-3’; IFNAR forward, 5’-TTGGGTGCTACCCTCAGC-3’, reverse, 5’-GCTGCTCCACTTTAGGGTGT-3’.

Lentiviral constructs and gene silencing by shRNA. Gene silencing sequences by short hairpin RNA (shRNA) of IRF7 (TRCN0000077288, TRCN0000077289, TRCN0000077290, TRCN0000077292) and non-silencing sequence (NS-shRNA) in pLKO.1-puro lentiviral vectors were purchased from Sigma-Aldrich. Lentivirus production, infection and selection with puromycin (5 µg/ml) were performed as previously described [1]. Gene knocking down efficiency were vilified by RT-qPCR, the most effective one (TRCN0000077289) were used for further experiment.

Microarray hybridization. Experiments were performed as previously described [1]. Briefly, triplicates wells of 4T1 and MR20 or DR500 cells grown in culture were used for RNA extraction using RNeasy kit (QIAGEN). Probe synthesis and GeneChip Mouse Gene Exon 1.0 ST Array (Affymetrix Ltd) hybridization were performed at the Genomic Technologies Facility (GTF, Lausanne, Switzerland) at UNIL (Lausanne).

Microarray data analysis. *Statistical analysis.* Microarray analyses were carried out with R, a free software environment available at http://www.r-project.org/. After quantification of

gene expression with robust multi-array normalization [2] using the BioConductor package Affy, (http://www.bioconductor.org/) significance of differential gene expression was determined by computing moderated t-statistics and false discovery rates with the limma package [3]. Annotation was based on the genome version NCBI Build 36 (Feb. 2006). The obtained p-values were corrected for multiple testing by calculating estimated false discovery rates (FDR) using the method of Benjamini-Hochberg. The transcriptional factors enrichment analysis was then performed by using MetaCore from Thomson Reuters (version 6.11) by filter with thresholds 1.5 and p-value 0.01. Heatmaps were produced by color-coding gene-wise standardized log gene expression levels (mean zero standard deviation one). Probe-sets were shown hierarchically clustered by similarity based on Euclidean distance and the ward aggregation algorithm. The data have been deposited in GEO (GSE100973). The interferon alpha gene signature was taken from MSigDB [4] and the interferon heatmap was drawn using the R package heatmap.

Expansion of relapsed tumor cells from MR20 bearing mice. To obtain the MR20 tumor cells that relapse from dormancy *in vivo*, relapsed tumors were processed as described for Flow cytometry analysis. After enzymatic treatment and filtering through a 100 µm and a 70 µm sterile nylon gauzes, tumor suspensions were cultured in 15 cm plate.

T cell depletion. *In vivo* depletion of CD4- and CD8-specific T cells was performed as descripted previously [5]. Briefly, 0.5 mg antibodies per mouse were injected intraperitoneally for 3 consecutive days. 6 days after the first injection, depletion efficiency was examined by flow cytometry from blood samples before tumor implantation. To maintain the depletion condition, same quantity of antibodies was injected twice per week.

Anti-CD4 (clone GK1.5), anti-CD8 (clone 2.43) antibodies and Rat IgG control (clone LTF-2) were purchased from BioXCell.

Flow cytometry analysis of blood samples. Peripheral blood leukocytes for phenotype analysis were obtained from the lateral tail vein or by terminal bleeding at days 7, 23 and 30 post-cancer cell injection. After red blood cells lysis with BD FACS lysing solution (BD Biosciences), leukocytes were washed stained with mentioned fluorescent antibodies.

Flow cytometry analysis on tissue samples. Mice were sacrificed at different time points (day 7, 15 and 30 post-cancer cell injection), after blood collection, mice were euthanized and tumors are dissected. Tumors were cut in small pieces with scissors, washed, and digested in serum free medium supplemented with Collagenase I (Worthington Biochemical Corporation), and DNAse I (Roche). The mixture was incubated at 37 °C for 45 minutes on a shaking platform. Subsequently, serum-supplemented medium was added to neutralize the enzymatic reaction and the tissue suspensions were filtered through a 100 µm and a 70 µm sterile nylon gauzes. Upon centrifugation (5 minutes at 1400 rpm), pellets were recovered and red blood cells lysed with ACK buffer. The staining procedure and the flow cytometry acquisition were the same as outlined above for blood. Data acquisition was performed using the FACSCalibur (BD Biosciences) or MACSQuant flow cytometer from MiltenyiBiotec and data analyzed by FlowJo v10.0.7.

Tissue morphology. Tumors and lungs were harvested at the end of the experiment, fixed in formalin and embedded in paraffin. 5 µm thick serial sections were cut from the tissue blocks. 3-4 sections that with 100 µm distance were stained with hematoxylin and eosin (H&E) and used to assess tumor morphology and quantify lung metastasis. Slides

were scanned by Nanozoomer (Hamamatsu Photonics) and metastasis are counted manually using NDP.viewer2 software (Hamamatsu Photonics).

Patients, clinical study and data analysis. The prospective multicentric TOP trial enrolled 149 ER-negative breast cancer patients between January 2003 and June 2008 that were treated with anthracycline monotherapy [6]. The present retrospective evaluation of the samples from this study was approved by the central ethics committees of the Institute Jules Bordet (20042017, CE2688). Blood samples were taken before therapy, at the end of the cycle one, and before surgery and were available for 51 patients.

Chi-squared tests were performed using chisq.test function, from package stats version 3.3.3, with default parameters. Kaplan-Meier curves were computed within R using the package survcomp version 1.24.0. Hazard ratios were computed by fitting a univariate proportional hazards regression model using package survival version 2.40-1.

Gene expression data for TOP study were retrieved from GEO database under the id GSE16446. Analysis were performed within R version 3.3.3. Each immune signature [7-9] was computed as a weighted average of the gene expressions included in the signature. Weights were set to -1 or +1 according to the sign of the original coefficients of the published signature. Signatures were scaled to insure reliable comparisons with the rescale function, from the package genefu version 2.6.0, applied with default parameters. This ensure that quantiles at 2.5% and 97.5% equal -1 and +1 respectively in each signature. Correlation matrix was built by computing Pearson coefficients for each pair of variables, using cor function from the package stats version 3.3.3. Only correlations having a p-value<0.05 were kept.

References to Materials and Methods

1. Monnier Y, Farmer P, Bieler G, Imaizumi N, Sengstag T, Alghisi GC *et al*. CYR61 and alphaVbeta5 integrin cooperate to promote invasion and metastasis of tumors growing in preirradiated stroma. Cancer Res 2008; 68: 7323-7331.

2. Irizarry RA, Hobbs B, Collin F, Beazer-Barclay YD, Antonellis KJ, Scherf U *et al*. Exploration, normalization, and summaries of high density oligonucleotide array probe level data. Biostatistics 2003; 4: 249-264.

3. Smyth GK. Linear models and empirical bayes methods for assessing differential expression in microarray experiments. Stat Appl Genet Mol Biol 2004; 3: Article3.

4. Liberzon A. A description of the Molecular Signatures Database (MSigDB) Web site. Methods Mol Biol 2014; 1150: 153-160.

5. Kruisbeek AM. In vivo depletion of CD4- and CD8-specific T cells. Curr Protoc Immunol 2001; Chapter 4: Unit 4 1.

6. Desmedt C, Di Leo A, de Azambuja E, Larsimont D, Haibe-Kains B, Selleslags J *et al*. Multifactorial approach to predicting resistance to anthracyclines. J Clin Oncol 2011; 29: 1578-1586.

7. Desmedt C, Haibe-Kains B, Wirapati P, Buyse M, Larsimont D, Bontempi G *et al*. Biological processes associated with breast cancer clinical outcome depend on the molecular subtypes. Clin Cancer Res 2008; 14: 5158-5165.

8. Teschendorff AE, Miremadi A, Pinder SE, Ellis IO, Caldas C. An immune response gene expression module identifies a good prognosis subtype in estrogen receptor negative breast cancer. Genome Biol 2007; 8: R157.

9. Perez EA, Thompson EA, Ballman KV, Anderson SK, Asmann YW, Kalari KR *et al*. Genomic analysis reveals that immune function genes are strongly linked to clinical outcome in the North Central Cancer Treatment Group n9831 Adjuvant Trastuzumab Trial. J Clin Oncol 2015; 33: 701-708.
